# Transcriptome Analysis of *Streptococcus mutans* Quorum Sensing-Mediated Persisters Reveals an Enrichment in Genes Related to Stress Defense Mechanisms

**DOI:** 10.3390/genes14101887

**Published:** 2023-09-28

**Authors:** Delphine Dufour, Haowen Li, Siew-Ging Gong, Céline M. Lévesque

**Affiliations:** Faculty of Dentistry, University of Toronto, Toronto, ON M5G 1G6, Canada; delphine.dufour@dentistry.utoronto.ca (D.D.); haow.li@mail.utoronto.ca (H.L.); sg.gong@dentistry.utoronto.ca (S.-G.G.)

**Keywords:** antibiotic persisters, streptococci, quorum sensing, defense systems, RNA sequencing

## Abstract

Persisters are a small fraction of growth-arrested phenotypic variants that can survive lethal concentrations of antibiotics but are able to resume growth once antibiotics are stopped. Their formation can be a stochastic process or one triggered by environmental cues. In the human pathogen *Streptococcus mutans*, the canonical peptide-based quorum-sensing system is an inducible DNA repair system that is pivotal for bacterial survival. Previous work has shown that the CSP-signaling peptide is a stress-signaling alarmone that promotes the formation of stress-induced persisters. In this study, we exposed *S. mutans* to the CSP pheromone to mimic DNA damage conditions and isolated the antibiotic persisters by treating the cultures with ofloxacin. A transcriptome analysis was then performed to evaluate the differential gene expression between the normal stationary-phase cells and the persisters. RNA sequencing revealed that triggered persistence was associated with the upregulation of genes related to several stress defense mechanisms, notably, multidrug efflux pumps, the arginine deaminase pathway, and the Opu/Opc system. In addition, we showed that inactivation of the VicK kinase of the YycFG essential two-component regulatory system abolished the formation of triggered persisters via the CSP pheromone. These data contribute to the understanding of the triggered persistence phenotype and may suggest new therapeutic strategies for treating persistent streptococcal infections.

## 1. Introduction

Most bacterial populations, if not all, generate persister cells that are highly tolerant to bactericidal antibiotics. Persisters are defined as phenotypic variants that can survive, through growth arrest, exposure to lethal concentrations of drugs [1]. Although they constitute a small subpopulation of cells within a genetically homogeneous bacterial population, they play a considerable role in the recalcitrance and relapse of infections, as they can survive an antibiotic treatment and re-establish a growing bacterial population upon the removal of the drug [2]. Moreover, persisters have also been associated with the accelerating evolution of antibiotic resistance, as they constitute a reservoir of viable cells available for resistance-conferring mutations and/or acquisition of resistance genes from the environment [3]. Two types of antibiotic persisters have been described: the spontaneously occurring persisters (spontaneous persistence) that are formed stochastically and the stress-induced persisters (triggered persistence) generated upon a stress signal. Spontaneous persistence occurs at a very low rate and when the bacterial cells are in steady-state exponential growth. This type of persistence is less common than triggered persistence. In contrast, many environmental stress conditions have been shown to contribute to the triggered persistence phenotype, such as nutrition starvation; oxidative, acid, and osmotic stresses; and extracellular signaling [1,4].

Over the past 20 years, the bulk of persister studies have focused on the triggers and pathways of persister formation. It has been proposed that persister formation is a general bacterial stress response and a core survival strategy in bacteria. In fact, most of the molecular pathways that have been identified as being involved in persister formation are stress response pathways, such as the stringent response with its key mediator guanosine tetra- and pentaphosphate (known as alarmone (p)ppGpp) activated upon exposure to nutrient limitation and the DNA damage inducible SOS response [4,5,6]. These stress response pathways lead to transcriptional rearrangements favoring the development of persisters. For example, the alarmone (p)ppGpp produced during nutrient stress causes the downregulation of genes responsible for growth and the activation of genes involved in amino acid synthesis and nutrient uptake. The role of (p)ppGpp in the formation of antibiotic persisters is particularly well-documented in *E. coli* and *Pseudomonas aeruginosa* [7,8]. The SOS response is activated upon exposure to various stress factors causing DNA damage, resulting in the upregulation of the SOS regulon. Bacteria encounter reactive oxygen species in abundance during interaction with a host, culminating in DNA damage [2]. More importantly, the SOS response may accelerate the emergence of antibiotic resistance where damage and/or error-prone repair result in mutations, which may select for antibiotic-resistant cells [9]. Therefore, elucidating the link between the SOS response and triggered persistence is important to the understanding of emergence of heritable antibiotic resistance.

In organisms lacking a classical SOS response, such as oral streptococci [10], the CSP-ComDE quorum-sensing system has been proposed to act as a general stress response [11,12,13]. The system is composed of the competence-stimulating peptide (CSP) pheromone, a small linear peptide encoded by the *comC* gene, and the ComDE two-component system [14]. The CSP pheromone is exported extracellularly through a dedicated ATP-binding cassette (ABC) transporter, where it accumulates. Upon reaching a threshold level, CSP interacts with the ComD membrane kinase, initiating a phosphorylation cascade leading to the activation of the cytoplasmic ComE regulator [15]. In the human oral pathogen *Streptococcus mutans*, the CSP pheromone is stimulated by several stress conditions (e.g., heat, acidic pH, oxidative stress, amino acid starvation) [16]. The CSP pheromone, thus, acts as a stress-signaling alarmone, triggering changes in gene expression to control different cellular functions, such as adaptation to acidic conditions, bacteriocin production for interspecies competition, biofilm development, and DNA repair and recombination [13]. Of particular interest, *S. mutans* has also been shown to use its CSP pheromone to increase the number of antibiotic persisters, and this triggered persistence is abolished in quorum sensing-deficient mutants [17,18].

In our previous study investigating the molecular mechanisms involved in triggered persistence in *S. mutans*, we isolated antibiotic persisters derived from normal cells treated with exogenous CSP pheromone to mimic cells exposed to DNA damage conditions [19]. We identified a DNA damage-inducible gene, *pep299*, that was activated in the persister subpopulation while being repressed in the actively growing normal bacterial population. These findings supported the notion that *S. mutans* persisters remained metabolically active and displayed an altered transcription profile with activation of some components of the bacterial stress response. These recent results prompted us to investigate in this current study the differential gene expression in triggered persisters using the CSP pheromone as a stress-inducible factor. Transcriptome sequencing (RNA-seq) was performed to evaluate the transcriptome of the *S. mutans* persisters isolated following treatment with ofloxacin antibiotic compared with that of the untreated parental strain, and differential gene expression was analyzed. We also assessed the roles of two genes activated in the persister population, VicK histidine kinase and a RelE-like toxin, for their role in the triggered persistence phenotype.

## 2. Materials and Methods

### 2.1. Streptococcal Strains and Growth Conditions

*S. mutans* UA159 strain (ATCC 700610) was used in this work. Todd–Hewitt broth (Thermo Fisher Scientific Inc., Mississauga, ON, Canada) supplemented with 0.3% (*w*/*v*) yeast extract (THYE) was used for bacterial cultivation. *S. mutans* cells were cultivated at 37 °C with 5% CO_2_. A nonpolar allelic replacement technique was used to generate the deletion mutants in the UA159 wild-type (WT) strain [20]. For the construction of the vector for toxin overexpression, the full-length coding region of the *relE40* gene was PCR-amplified using UA159 genomic DNA and a PCR product cloned under the control of the constitutive promoter P_23_ into the *E. coli Streptococcus* shuttle plasmid pIB166 [21] harboring a chloramphenicol resistance cassette. The construct was then transferred into the UA159 WT strain via natural transformation [22].

### 2.2. Preparation of Bacterial Inoculum for Persister Cell Collection

A standardized procedure for inoculum preparation was designed based on the protocol developed by Kaldalu et al. [23]. Briefly, *S. mutans* cultivated on the surface of THYE agar was used to inoculate 3 mL of THYE broth. Following overnight incubation, the culture was diluted (1:20) in fresh liquid THYE and grown until an optical density (OD) of 0.5 at 600 nm was reached. Sterile glycerol was added at a final concentration of 15% (*v*/*v*) to the culture for storage at −80 °C [19].

### 2.3. Persister Cell Collection for RNA Sequencing

Overnight cultures (~20 h) prepared in THYE were diluted (1:100) in 200 mL of fresh THYE liquid broth and incubated for 2 h at 37 °C with 5% CO_2_ in the presence of 50 ng/mL of CSP pheromone (Shanghai Royobiotech, Ltd., Shanghai, China). The cultures were then treated with 20 µg/mL of ofloxacin, corresponding to 10 times [24] the minimum inhibitory concentration (MIC) for 22 h at 37 °C. Importantly, ofloxacin was shown to kill normal-growing cells and nongrowing stationary-phase cells, leaving intact persisters [25]. The cultures were washed with sterile phosphate-buffered saline (PBS) and the isolated persisters (~2 × 10^6^ CFU) were collected via centrifugation for RNA extraction. A control of normal stationary-phase cells induced with the CSP pheromone was prepared as described above but without the ofloxacin treatment.

### 2.4. RNA Extraction and RNA Sequencing

Bacterial cells (persisters and normal cells) were resuspended in ice-cold RNAwiz™ solution (Thermo Fisher Scientific Inc.). Following cell disruption, total RNA was extracted using a RiboPure-Bacteria purification kit (Thermo Fisher Scientific Inc.) according to the manufacturer’s recommendations. RNA was DNase-treated with RQ1 DNase (Promega, Madison, WI, USA) and purified using an RNeasy MinElute Cleanup kit (Qiagen, Toronto, ON, Canada). RNA quality was evaluated using an Agilent 2100 Bioanalyzer (Agilent Technologies, Mississauga, ON, Canada), and all RNAs used for downstream experiments were determined to have RNA integrity numbers (RIN) of 7.0 and above. Library preparation was performed using an Illumina Stranded Total RNA Prep Ligation with Ribo-Zero Plus kit (Illumina, San Diego, CA, USA) with 100 ng of RNA. Enriched libraries were pooled and sequenced on a MiSeq instrument (Illumina) according to the manufacturer’s recommendations for paired-end 75 base-pair reads.

### 2.5. RNA Sequencing Data Analysis

Illumina bcl2fastq Conversion software V2.20 was used for demultiplexing and to convert the base call files generated by the Illumina sequencing into FASTAQ files. Quality of the raw data was assessed with FastQC (v0.11.9). Removal of sequencing adapters and low-quality ends was performed using Trim Galore (v0.5.0), and the QC checks were repeated with FastQC to ensure that high-quality data were retained. The reads were then aligned to the *S. mutans* UA159 complete genome (NC_004350.2) [26] downloaded from NCBI using STAR aligner (v2.6.0c). RSeQC package (v2.6.2) was used to calculate read distribution, positional read duplication, and gene body coverage, as well as for confirmation of strandedness. The htseq-count script (v0.6.1p2) was executed in the intersection_nonempty mode to quantify the processed reads aligning to a set of nonredundant features. DESeq2 (v1.22.2) was used to perform a principal component analysis and visualize the sample clustering. Two-condition differential expression was performed with the R package edgeR (v3.28.1) and DESeq2 (v1.22.2). Genes were considered differentially expressed when the Benjamini–Hochberg multiple-testing-adjusted *p*-value was less than 0.05 (adj *p*-value ≤ 0.05). Significantly differentially expressed protein-coding genes were classified into clusters of orthologous groups of proteins (COGs) using the MicroScope platform (v3.16.1).

### 2.6. Gene Expression Analysis via RT-qPCR

For the analysis of *comC* gene expression in stationary-phase persisters, overnight cultures were diluted (1:100) in fresh THYE broth and incubated at 37 °C for ~10 h. Stationary-phase cultures were then exposed to 20 µg/mL of ofloxacin for 24 h at 37 °C. Cells were harvested via centrifugation and washed with sterile PBS before being processed for total RNA extraction using a RiboPure-Bacteria purification kit (Thermo Fisher Scientific Inc.), as described previously [19]. Quantitative reverse-transcription PCR (RT-qPCR) analysis was performed using Forget-Me-Not EvaGreen qPCR Master Mix (Biotium, Fremont, CA, USA) and a CFX96 real-time PCR detection system (Bio-Rad, Mississauga, ON, Canada). Data analysis was performed using relative quantification normalized against unit mass. For the analysis of *relE40* gene expression, overnight cultures were diluted (1:100) in fresh THYE broth and incubated until mid-log phase. Cultures were then exposed for 2 h at 37 °C to the following DNA damage conditions: quinolone (2 µg/mL), mitomycin C (0.5 µg/mL), hydrogen peroxide (0.5 mM), and pH 5.0. Cells were then harvested via centrifugation and washed with PBS before being processed for total RNA extraction [19]. RT-qPCR analysis was performed as described above. Primers used for RT-qPCR are listed in Table S1.

### 2.7. Persister Assay

Overnight cultures (~20 h) were diluted in 5 mL of THYE liquid broth supplemented with 10× MIC of ofloxacin antibiotic and incubated at 37 °C to kill all nonpersisters. At the indicated time points, culture samples withdrawn were washed with PBS, serially diluted, and plated on THYE agar plates. The percentage of persisters was determined via colony-forming unit (CFU) counts after 48 h of incubation at 37 °C. All assays were carried out in triplicate, and the data were analyzed using the unpaired *t*-test with statistical difference defined as *p* < 0.05.

### 2.8. Toxin Expression in E. coli

To generate an inducible expression construct, the open-reading frame of the relE40 toxin gene was cloned upstream from the His_6_ sequence under the control of the *ara*BAD promoter into the pBAD202/D-TOPO vector (Thermo Fisher Scientific Inc.), following the manufacturer’s recommendations. The recombinant plasmid was transferred into LMG1994 cells to express the His_6_-tagged recombinant toxin. Overnight culture was diluted (1:100) in Luria–Bertani (LB) broth containing kanamycin at 50 µg/mL and grown to an OD_600_ of 0.5. The culture was divided into two equal parts: one was grown in the presence of 0.2% (*w*/*v*) arabinose (for induction of *ara*BAD promoter), while the other one was grown in the presence of 0.2% (*w*/*v*) glucose (uninduced control). Aliquots of cultures were removed at 1, 2, 3, and 4 h postinduction, serially diluted, and plated on LB-kanamycin agar for CFU determination [27].

## 3. Results

### 3.1. Persisters Are Metabolically Active Cells, and Their Population Is Enriched at Stationary Phase

**Figure 1:**
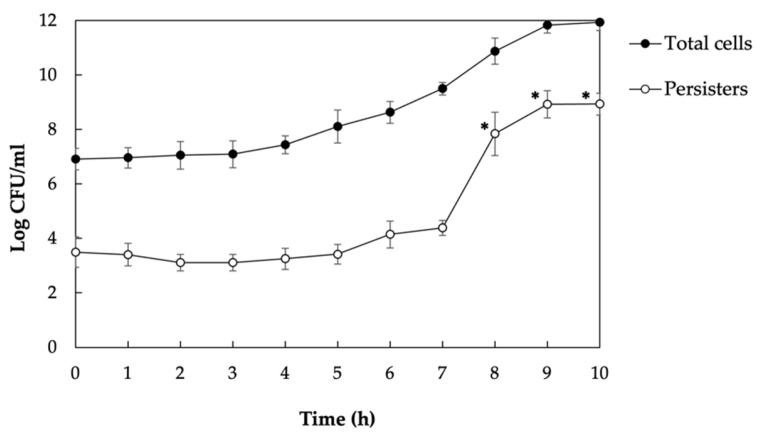
Formation of *S. mutans* persisters. Overnight precultures of UA159 WT strain were diluted (1:100) in fresh broth and incubated at 37 °C. Aliquots of cultures were harvested hourly and treated with ofloxacin antibiotic for 24 h at 37 °C. Samples were washed in PBS, serially diluted, and plated on THYE agar plates. Viable cell enumeration (CFU/mL) via plate counting was used to determine the total number of cells (untreated) and the number of persisters harvested following antibiotic treatment. Data are the averages of three independent experiments ± SD. * denotes statistical significance difference in the number of antibiotic persisters vs. t = 0.

We first performed a growth kinetic to quantify the amount of *S. mutans* persisters. Aliquots of *S. mutans* cultures were harvested hourly and treated with 10× the MIC of ofloxacin antibiotic to kill all nonpersister cells. The use of a high concentration of bactericidal antibiotic is necessary to prevent the development of resistant mutants that can be observed when an intermediate concentration slightly above the MIC is used [28]. Control experiments were also performed to confirm the absence of spontaneous antibiotic-resistant mutants. The results showed that stationary-phase cultures survived bactericidal drug treatments better than the growing cultures (Figure 1). In fact, persister levels did not increase in exponentially growing cultures, but their levels increased significantly when cells entered the stationary phase. Prolonged antibiotic treatments (48–72 h) did not affect the killing rate, confirming that *S. mutans* persisters can sustain longer antibiotic treatments without being antibiotic-resistant (Figure S1).

Recent studies in *S. mutans* and pneumococcus suggested that the CSP pheromone may serve as a probe for sensing environmental cues such as stationary-phase stress conditions [15]. We first conducted RT-qPCR to measure the expression of the CSP-encoding gene (*comC*) in the persister population vs. normal stationary-phase cells. Interestingly, the *comC* gene was found activated in the persisters (+4.77 ± 1.27) vs. normal cells. As expected, *gyrA* encoding the DNA gyrase subunit A (enzyme essential for DNA replication) was not expressed in the persister population [19]. These results confirmed that *S. mutans* persisters are metabolically active cells but in a nondividing state. We next inactivated the CSP-encoding gene in the UA159 WT strain, and the mutant was assayed for formation of antibiotic persisters at the stationary phase. Our results showed that the level of persisters was strongly reduced (−152.37 ± 32.36) in the ΔcomC mutant vs. WT. In line with previous findings [17], our results suggested that *S. mutans* may use its CSP pheromone to prepare for a ‘stress response’ state via formation of persisters in order to survive.

### 3.2. Isolation and Transcriptome Analysis of Persisters

**Figure 2:**
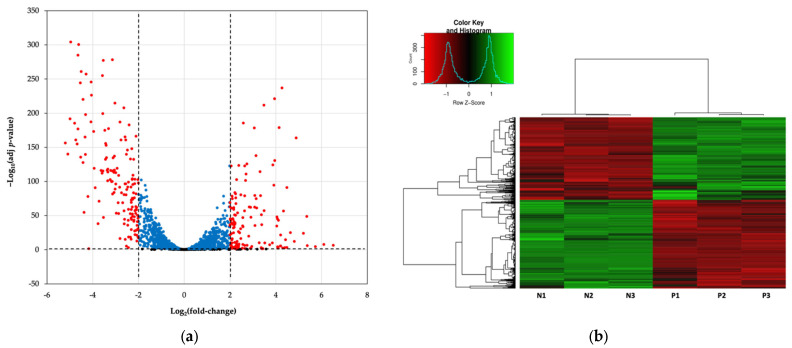
Overview of RNA-seq. (**a**) Volcano plot displaying differentially expressed genes (DEGs) between CSP-induced persisters (‘persisters’) vs. CSP-induced normal cells (‘normal cells’) in the stationary phase. Volcano plot shows the distribution of significance [–log_10_(adj *p*-value)] vs. fold change [log_2_(fold-change)] for all genes. The vertical lines indicate a log_2_(fold-change) cutoff ± 2. The horizontal line represents the statistical significance threshold of adjusted *p*-value ≤ 0.05. Red dots: *p*-value and log_2_(fold-change); blue dots: *p*-value; black dots: nonsignificant. (**b**) Hierarchical-clustering heat map showing the 1431 significant DEGs with adj *p*-value ≤ 0.05. Heat map shows the clustering of three technical replicates from two samples (N: normal cells; P: persisters). The green and red colors represent upregulation and downregulation, respectively.

To shed light on the molecular mechanism of persister formation via the CSP pheromone, the regulation of gene expression in the presence of CSP was explored via RNA-seq using the UA159 WT strain. RNA-seq was performed to evaluate the differences between the gene expression of CSP-induced stationary-phase cells (‘normal cells’) and CSP-induced stationary persisters (‘persisters’). The volcano plot displays the global transcriptional response of persisters (Figure 2a). The comparison between normal cells and persisters made it possible to identify a total of 1431 differentially expressed genes (DEGs; adj *p*-value ≤ 0.05). These are represented in the heat map shown in Figure 2b.

**Figure 3:**
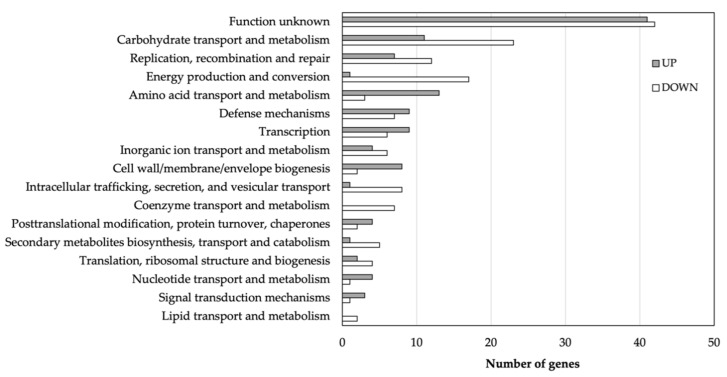
COG functional analysis of the identified proteins. A total of 118 upregulated and 148 downregulated genes were identified in the persister population.

In comparison to normal cells, a total of 302 DEGs (*p*-value ≤ 0.05 and log_2_FC ± 2) were identified in the persister population. From these DEGs, 141 were found upregulated and 161 downregulated. A clusters of orthologous gene (COGs) distribution analysis was performed using the MicroScope platform [29]. A total of 266 DEGs showing significant homology to those in the COG database were functionally grouped into 17 categories (Figure 3).

In persisters vs. normal cells, genes encoding hypothetical proteins were highly represented in the list of up- and downregulated genes, accounting for ~30% of the total number of DEGs. The downregulated DEGs were enriched in three biological categories, including replication, recombination, and repair (e.g., *dprA*, *topA*, *radC*); energy production; and carbohydrate transport and metabolism (e.g., *citDEF*, *msm* locus, PTS loci). The most significant upregulated biological processes in the persisters were related to amino acid transport and metabolism, defense mechanisms, and cell wall biogenesis. A subset of DEGs was selected and used for RT-qPCR analysis for validation of the RNA-seq data (Table S1). The RT-qPCR results obtained were consistent with the expression trends observed in the RNA-seq. The entire list of the upregulated and downregulated DEGs can be found in Tables S2 and S3, respectively. An Excel file with all the RNA-seq data can be found in Table S4.

### 3.3. Genes Related to Metabolism of Galactose/Lactose

Overall, most genes related to energy production and carbohydrate transport were found to be downregulated in the persister state. However, one locus harboring genes coding for the tagatose pathway for the metabolism of lactose/galactose was strongly upregulated. These genes included the lactose-specific PTS transporter (*lacF*, *lacE*), a phospho-β-galactosidase (*lacG*), and the enzymes of the tagatose-6-phosphate pathway (*lacAB*, *lacC*, *lacD*). Interestingly, the enzymes (*galK*, *galT*, *galE*) involved in the Leloir pathway for the catabolism of galactose-6-phosphate were all found downregulated in the persisters.

### 3.4. Genes Related to Stress Defense Mechanisms

Several genes encoding for proteins involved in stress defense mechanisms were differentially transcribed between the persister population and normal cells (Table 1).

Of particular interest were two genes encoding peptide toxin/antimicrobial (SMU.40, SMU.299). SMU.40 encodes a RelE-like toxin and was the most upregulated DEG in the persister population, with a log_2_FC of +6.10. RelE toxin is usually part of a RelE–RelB toxin–antitoxin gene pair [30]. Examination of the DNA sequence surrounding SMU.40 did not exhibit similarity with RelB antitoxin or any putative antitoxins. These results suggested that SMU.40 may be a divergent or orphan toxin. SMU.299 encodes a small di-glycine signature peptide [31]. Recent work by our group showed that SMU.299 was activated when cells were exposed to DNA damage conditions, and its expression promoted the formation of antibiotic persisters [19]. The fact that SMU.299 was detected in our RNA-seq analysis supports our previous results, suggesting that Pep299 is at the core of the triggered persistence phenotype in *S. mutans* [19].

Interestingly, the persistence state induced the differential transcription of two genes related to biofilm formation, *gbpC* and *spaP*, encoding a glucan-binding protein and cell surface antigen I/II, respectively. GbpC and AgI/II are two cell surface-anchored proteins that were shown to be critical to *S. mutans* adherence and biofilm formation [32]. None of the other cell surface-anchored proteins harboring an LPxTG motif for sortase-mediated cell wall anchoring [33] were differentially expressed in the persister population.

Our results showed that three genes encoding multidrug efflux pumps were among the upregulated DEGs in the persisters. These genes included SMU.1286, SMU.2109, and SMU.71. A BLAST search analysis revealed that both SMU.1286 and SMU.2109 genes encoded putative permeases of the major facilitator superfamily, while SMU.71 shared high similarity to the MATE (multidrug and toxic compound extrusion) family [34], the most recently categorized family of multidrug efflux transporters.

The arginine deiminase (AgDI) pathway (classified in amino acid transport and metabolism in the COG database) encoded by the operon *aguBDAC* was highly upregulated (log_2_FC > 4) in the persister population. The AgDI pathway allows the hydrolysis of agmatine into putrescine, NH_3_, CO_2_, and ATP and is a response to acid and starvation conditions in lactic acid bacteria [10]. In *S. mutans*, several environmental stress conditions were shown to induce the expression of *aguD*, implying that AgDI may be part of the general stress response of this species [35].

Few oxidative stress survival genes were found differentially transcribed in the persister population. In particular, *sodA* (superoxide dismutase), *tpx* (thiol peroxidase), *nox* (H_2_O-forming NADH oxidase), and SMU.1297 encoding a 3′-phosphoadenosine phosphatase involved in superoxide stress tolerance were found significantly upregulated in the persister state. Interestingly, the main transcription factors (SpxA1, SpxA2, Rex, PerR) involved in *S. mutans* oxidative stress adaptation [36] were not among the DEGs. In fact, only the metalloregulatory protein SloR was found significantly regulated in the persisters, with a log_2_FC of −2.58.

Previous work suggested a possible crosstalk between the oxidative and osmotic stress responses in *S. mutans* [37]. The UA159 genome encodes two regions with homology to the Opu/Opc family of ABC transporters involved in the response to hyperosmotic conditions. The Opu/Opc systems play a key role by importing osmoprotectants [32]. We observed that both systems encoded in UA159 were upregulated in the persisters. The first locus (*opuABC*, *opuAA*) coded for a proline/glycine betaine transport system, while the second locus contained four genes (*opcA*, *opuBB*, *opcC*, *opuCD*) encoding a glycine betaine/carnithine/choline ABC transporter [38]. Although *opuCD* had a log_2_FC of less than 2.0 (log_2_FC = 1.32) probably due to transcription distance, the gene was most likely upregulated.

As global transcriptional regulators regulate a variety of cellular processes including stress survival, we analyzed the RNA-seq for potential regulatory factors. Of interest was the tricistronic operon *vicRKX* encoding a response regulator (VicR), its cognate membrane kinase (VicK), and a putative hydrolase (VicX). In *S. mutans*, the VicRK system is considered essential and a key regulator of acid and oxidative stress responses, competence development, and biofilm formation [39]. Other genes with increased expression profiles in the persister population included NusA, which is important for the coordination of cellular responses to DNA damage [40]. Two genes (*mutY*, *smx*) encoding proteins of the BER pathway for DNA repair [41] were also among the upregulated DEGs in the persisters. Among the three Clp ATPase subunit-encoding genes identified in the genome of UA159, only *clpE* was found activated in the persister population.

### 3.5. Inactivation of VicK Abolished the Triggered Persistence Phenotype

**Figure 4:**
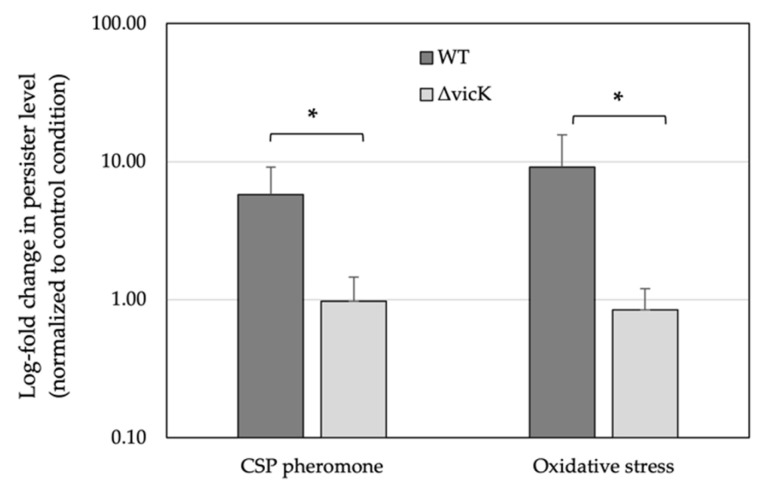
Formation of persisters by UA159 WT strain and its ∆vicK null mutant. Overnight cultures were diluted (1:100) in fresh broth and subjected to the CSP pheromone (50 ng/mL) or hydrogen peroxide (0.5 mM) for 2 h at 37 °C before being treated with ofloxacin for 22 h at 37 °C. CFU determination was used to determine the persister level (see Materials and Methods for details). The data are the averages of six independent experiments ± standard error. * denotes statistically significant difference compared to WT under the same conditions.

In order to verify if the VicRKX system was involved in the triggered persistence phenotype, we constructed a *vicK* knockout strain (∆vicK), as *vicR* was reported to be essential for cell viability in *S. mutans* and several other related pathogens [42]. We first confirmed that, during the stationary phase of growth, the number of cells in the WT and ∆vicK null mutant reached the same level (~10^9^ CFU/mL). We next exposed both strains to the CSP pheromone and determined the level of persister formation following treatment with ofloxacin. Compared with the WT strain, ΔvicK had an ~6-fold reduction in the persister levels (Figure 4). In light of these findings, we next investigated the effect of hydrogen peroxide to mimic oxidative stress on persister formation. As expected, the level of persisters was drastically reduced (~10-fold) in the ΔvicK null mutant.

### 3.6. Overexpression of relE40 Promotes the Formation of S. mutans Persisters

**Figure 5:**
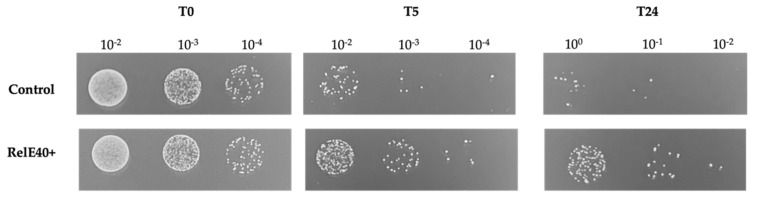
Formation of persisters in *S. mutans* cells overexpressing *relE40*. Cultures were treated with ofloxacin to kill all nonpersisters. Aliquots of cells were removed at the introduction of the antibiotic (T0) and after 5 h (T5) and 24 h (T24) of antibiotic treatment. Cells were washed with PBS, serially diluted (10^0^; 10^−1^; 10^−2^; 10^−3^; 10^−4^), and spot-plated on THYE agar. Pictures shown are representative of three independent experiments. Control: *S. mutans* WT harboring an empty pIB166 plasmid. RelE40+: WT harboring pIB166-*relE40*.

Since SMU.40 was the DEG with the highest expression in the persister state, we performed additional experiments to further characterize the impact of the gene in the persister formation. A bioinformatic analysis showed that SMU.40 encoded a putative toxin of the RelE/ParE family. A sequence comparison of the putative RelE toxin of *S. mutans* with the RelE2 toxin conserved in *Streptococcus pneumoniae*/*E. coli* showed that the first 36 amino acids were absent in *S. mutans*. This prompted us to investigate the functionality of the putative toxin by cloning the open-reading frame upstream of the inducible arabinose promoter *ara*BAD into the pBAD202/D-TOPO vector for a toxicity assay. The overproduction of SMU.40 in *E. coli* led to cell growth arrest (Figure S2). These results suggested that, although SMU.40 encoded a N-terminally truncated RelE-like peptide (named *relE40* gene hereafter), it retained its growth-inhibitory activity in *E. coli*. Therefore, we next tested the direct impact of the *relE40* gene in the development of persisters using *S. mutans* WT cells overexpressing the gene under the control of a constitutive promoter. Our results showed that the level of persisters was statistically significantly higher (greater than 10-fold) in the RelE40+ strain when compared to the WT control harboring the empty plasmid (Figure 5).

We next investigated the expression profile of *relE40* under DNA damage stress conditions known to favor the triggered persistence phenotype in *S. mutans*. As shown in Table 2, the *relE40* gene was found upregulated under all tested stress conditions. These findings suggested that, under unfavorable conditions, *S. mutans* may modulate the expression of *relE40* encoding a toxic peptide, leading to a higher persister cell frequency.

## 4. Discussion

Antibiotic treatment failure has largely focused on antibiotic resistance [3]. Increasing evidence suggests that bacterial persisters play a considerable role, as they are extremely tolerant to all antibiotics to which they are genetically susceptible. Antibiotic persisters are transient phenotypic variants of regular cells that have the ability to resume growth upon removal of the antibiotic or when the antibiotic levels fall below the lethal dose [1]. The vast majority of antibiotics display poor efficacy against persisters, as they generally exist in a growth-arrested state. While persisters constitute a small fraction of the antibiotic-susceptible population, environmental stress conditions positively influence the number of persisters, and their levels can reach up to 1% in biofilms [43]. Quorum-sensing systems are communication networks that enable bacteria to sense and respond to environmental conditions through the exchange of signaling molecules [44]. Quorum sensing was linked to persister formation in *S. mutans* (CSP), *P. aeruginosa* (HSL, PCN, 2-AA), and *Staphylococcus aureus* (Agr) [45]. Although not considered a true quorum-sensing molecule, the aromatic secondary metabolite indole produced by over 85 bacterial species was also shown to affect persister formation [45]. However, whether indole promotes or represses the formation of persisters is still under debate. The importance of cell–cell signaling in bacteria has exploded over the past decade; therefore, we may discover that many more signaling molecules are involved in persister formation. The first report of quorum sensing-mediated persistence in Gram-positive bacteria was documented in *S. mutans* [17], the main etiologic agent dominating the dental caries dysbiotic ecosystem [46]. *S. mutans* is also considered a model organism for Gram-positive pathogens that share a host-associated lifestyle [47]. In *S. mutans*, there is an interconnection between a cell’s ability to regulate bacteriocin production and the triggered persistence phenotype, as both processes are activated by the CSP pheromone [15]. As *Streptococcus pneumoniae* encodes a locus with high homology to the *S. mutans comDE* (named *blpRH*) [48] that is also involved in the regulation of pneumococcal bacteriocins, we can speculate that the BlpS pheromone, the peptide activating BlpRH-dependent genes, may participate in the formation of stress-mediated persisters in pneumococci.

Numerous studies have focused on understanding the different pathways and mechanisms involved in the formation of bacterial persisters. Despite the diverse genes being studied (e.g., toxin–antitoxin modules, RelA, RpoS, etc.), there is unlikely to be a single pathway that explains persister formation. Several studies are now being undertaken to determine the gene expression profiling of antibiotic persisters. In *S. mutans*, the persister cell phenotype is dominated by persisters generated upon a stress signal (previously called type-I persistence) [1]. Inspired by previous work conducted using isolated total RNA from persister populations (e.g., *E. coli*, *Mycobacterium tuberculosis*) obtained through exposure to high concentrations of antibiotics that eliminate nonpersisters, we sought to determine the global transcriptional changes in *S. mutans* persisters. Since several stress conditions have been shown to positively influence the formation of persisters in *S. mutans* [17], we incubated the cultures with exogenous CSP pheromone to mimic the harsh but nutrient-rich oral cavity. The transcriptomic analysis was performed to identify DEGs in the stationary-phase population of persisters vs. normal cells. The results revealed that several genes were differentially regulated in the persister population. We focused our analysis on the upregulated DEGs, as these are likely involved in persister formation and/or maintenance [49]. Consistent with the general trend observed in other transcriptomic studies of persister-enriched samples, the phenomenon of triggered persistence in *S. mutans* seemed multifactorial, with different mechanisms playing roles such as proteolytic degradation (e.g., ClpE), membrane stabilization (e.g., AgDI), and efflux pumps. In fact, our findings pointed toward a complex network that transduces stress responses via the canonical quorum-sensing pathway to yield antibiotic persisters. For example, cells sense environmental perturbations through two-component signal transduction systems (e.g., CSP, VicRK), osmotic sensors, thiol sensing, etc. Downstream signaling then causes activation of cellular strategies to cope with stresses (e.g., osmotic pressure, toxic reactive oxygen species, thiol stress) and accumulation of stress-related molecules that are linked to increased persister formation, such as Pep299 and RelE40.

Interestingly, genes encoding multidrug efflux pumps were among the significantly upregulated DEGs. SMU.2109 (NorA-like) and SMU.1286 (PmrA-like) showed significant similarity (over 45%) to the NorA multidrug resistance pump of *S. aureus* and PmrA of *S. pneumoniae*, respectively. Studies conducted on *E. coli* demonstrated that, although persisters were in a growth-arrested mode, they used active efflux to pump antibiotics out of the cell to survive antibiotic treatment [50]. More recently, work conducted using *Streptococcus pyogenes* persisters in a biofilm-like environment also showed efflux activity in persisters [51]. To the best of our knowledge, this is the first study reporting the upregulation of multidrug efflux pumps in quorum sensing-mediated persisters. Further work is required to test whether the upregulation of *S. mutans* efflux pumps correlates positively with the frequency of antibiotic persisters. We are currently exploring this possibility.

Over the years, only a few genes without any role in stress response have been found upregulated in triggered persisters. Such genes include the flagellar genes (basal body, export, stator) in *E. coli*. Using a transposon insertion library, authors showed that a deficiency in motility significantly diminished persisters tolerant to the aminoglycoside gentamicin [52]. Of particular interest to *S. mutans*, we identified two genes, *gpbC* and *spaP*, that were significantly upregulated in the persister state. Both GbpC and SpaP (also known as PAc and AgI/II, respectively) are surface-anchored protein-mediating adhesions, and they play an important role in the initiation of biofilm formation in *S. mutans* [53]. Persisters exhibiting multidrug tolerance are at the core of biofilm infections. Although more studies are required to elucidate their importance in persister formation, the fact that biofilm-specific genes were found activated may have disastrous consequences, as the development of a biofilm provides functional benefits to the persisters by ensuring protection from elimination by the immune system [54].

A potential limitation of our study is the possible effect of antibiotic-dependent switches. Future studies using different classes of antibiotics are required to determine whether they give rise to the same persister transcription profile as the one obtained with ofloxacin. Second, the fact that several genes encoding hypothetical proteins were found activated in the DEGs led us to question whether these hypothetical genes are conserved among persisters in other species. Criteria need to be defined to prioritize genes to be experimentally studied. To conclude, our work confirmed that the canonical quorum-sensing system of *S. mutans* actively upregulates a subset of stress defense genes to maintain a population of antibiotic persisters and, most likely, tolerance to environmental stresses. The finding that the quorum-sensing CSP pheromone is a ‘persister-inducible factor’ suggests that quorum-sensing inhibitors or quenching enzymes could be an attractive strategy for the development of novel antipersister drugs. Moreover, the fact that quorum-sensing systems are also key factors that promote the development of biofilms for several pathogens makes the strategy of quorum inhibition even more attractive. Interestingly, the canonical quorum-sensing system of *S. mutans* is found in all seven streptococcal groups (mutans, pyogenic, salivarius, bovis, suis, mitis, anginosus) [48]. Although further work is required to confirm their role in triggered persistence, the high prevalence of the system in all streptococcal groups strongly supports the idea that quorum sensing-mediated persistence is a widespread mechanism. Finally, since most bacterial infections are associated with mixed-species biofilms, it is of critical importance to examine the role of quorum-sensing signaling molecules in more complex biofilm communities.

## Figures and Tables

**Table 1:** Stress defense mechanism-related genes upregulated in persisters.

Biological Process	Gene	Functional Protein	Log_2_FC
Peptide toxin/antimicrobial	SMU.40	RelE-like toxin	6.10
	SMU.299	Pep299	2.48
Biofilm	SMU.1396	GbpC	5.14
	SMU.610	SpaP	2.05
Efflux pump	SMU.1286	Multidrug efflux pump	3.09
	SMU.2109	Multidrug efflux pump	2.46
	SMU.71	Multidrug efflux pump	2.42
Acid/starvation stress	SMU.262	AguB; putrescine carbamoyl transferase	4.21
	SMU.263	AguD; agmatine/putrescine antiporter	4.64
	SMU.264	AguA; agmatine deiminase	4.43
	SMU.265	AguC; carbamate kinase	4.81
Oxidative stress	SMU.1297	3′-phosphoadenosine phosphatase	3.33
	SMU.924	Tpx; thiol peroxidase	2.55
	SMU.629	SodA; superoxide dismutase	2.10
	SMU.1117	Nox; H_2_O-forming NADH oxidase	2.00
Osmoprotectant	SMU.1062	Proline/glycine betaine permease	3.48
	SMU.1063	ATP-binding protein	4.15
	SMU.2116	ATP-binding protein	3.31
	SMU.2117	Membrane permease	2.91
	SMU.2118	Substrate-binding protein	2.16
	SMU.2119	Membrane permease	1.32
Global stress response	SMU.418	NusA; transcription factor	2.74
	SMU.1515	VicX; hydrolase	2.21
	SMU.1516	VicK; histidine kinase	2.00
	SMU.1517	VicR; response regulator	2.13
	SMU.1649	Smx; exonuclease	2.70
	SMU.1865	MutY; DNA glycosylase/lyase	2.00
	SMU.562	ClpE; Clp ATPase	2.58

**Table 2:** Gene expression of *relE40* by RT-qPCR.

Stress Condition	Fold-Change (Stress/No Stress)
Quinolone	19.49 (±6.68)
Mitomycin C	3.64 (±0.65)
Hydrogen peroxide	2.07 (±0.31)
THYE broth at pH 5	3.54 (±0.52)

## Data Availability

Not applicable.

## Supplementary Materials

The following supporting information can be downloaded at: https://www.mdpi.com/article/10.3390/genes14101887/s1, Table S1: Primer pairs used for RT-qPCR; Table S2: DEGs significantly upregulated (log_2_FC ≥ 2) in the persister population; Table S3: DEGs significantly downregulated (log_2_FC ≤ −2) in the persister population; Table S4: Complete RNA-seq data; Figure S1: Levels of *S. mutans* persisters following treatment with ofloxacin up to 72 h; Figure S2: Expression of SMU.40 gene (*relE40*) in *E. coli*.
