# Supplementary material for: Transcriptome Analysis of Streptococcus mutans Quorum Sensing-Mediated Persisters Reveals an Enrichment in Genes Related to Stress Defense Mechanisms
Source: Genes (Basel). 2023 Sep 28;14(10):1887. doi: 10.3390/genes14101887 (PMC10606796; doi:10.3390/genes14101887)
Supplement: Supplementary file 1 [file genes-14-01887-s001.zip › genes-2617115-supplementary.pdf]

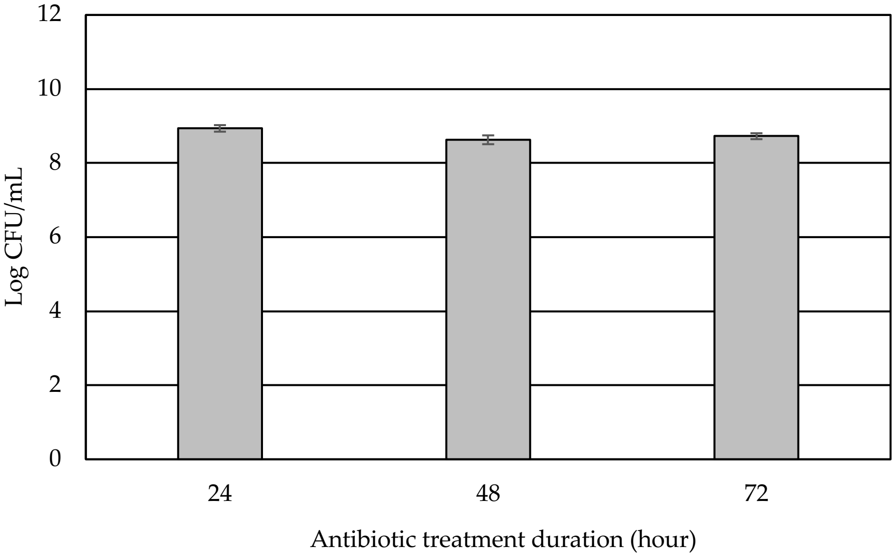

Figure S1. Levels of *S. mutans* persisters following treatment with ofloxacin up to 72 h. Stationary grown *S. mutans* UA159 wild-type cells were treated with 20 μg/mL of ofloxacin antibiotic for 24 h, 48 h, and 72 h at 37°C. Collected samples were washed once in phosphate-buffered saline, serially diluted, and plated on THYE agar plates. Viable cell enumeration (CFU/mL) by plate counting was used to determine the number of persisters. Bars presented are the averages and standard deviations of the results from three independent experiments.

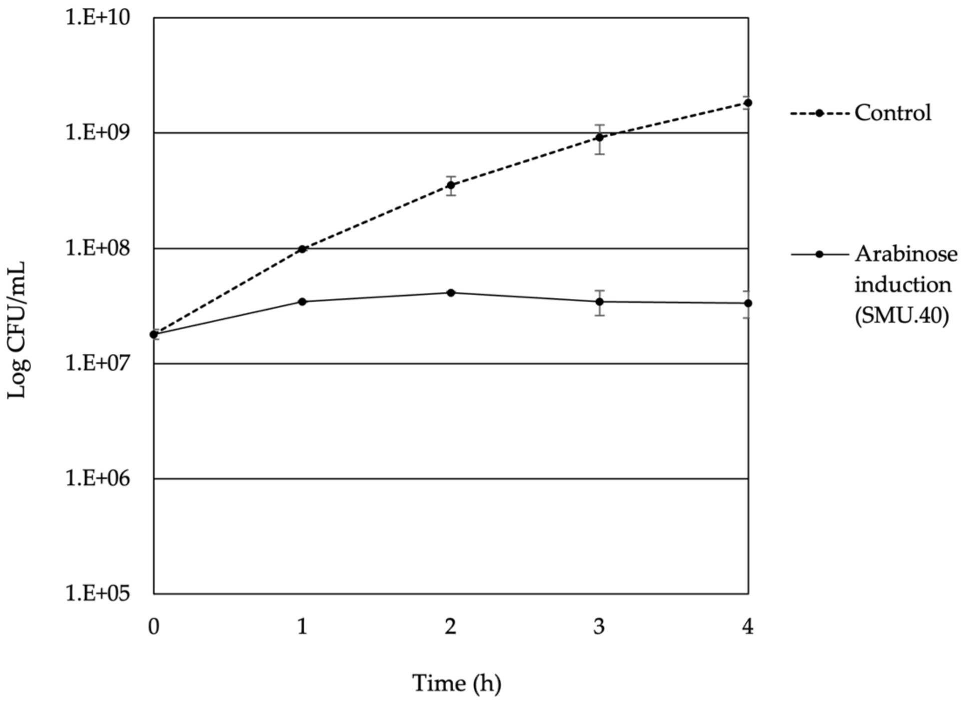

Figure S2. Expression of SMU.40 gene (*relE40*) in *E. coli*. Cells of LMG194 containing pBAD-relE40 were grown to mid-log phase (OD_{600} of 0.5), at which time 0.2% (*w*/*v*) arabinose (induction) and 0.2% (*w*/*v*) glucose (control) were added. After induction, appropriate dilutions were plated on LB agar supplemented with kanamycin (50 μg/mL) for determination of the number of colony forming units (CFU) per ml. The curves presented are the averages and standard deviations of the results from three independent cultures.

Table S1. Primer pairs used for RT-qPCR.

Gene ID	Forward primer (5′ – 3′)	Reverse primer (5′ – 3′)
SMU.22	AGCAACAGAAGCACAACCATCAG	CCACCATTACCCCAGTAGTTTCC
SMU.40	GACAATACAAGACAGCAAAGC	GCTACAATAATCAGCCCCTC
SMU.124	TTCTATACTGGCGACGTCTG	ATTGTTTGCCAACTTCCGTC
SMU.299	ACGATGGAGCTAATGGCTAT	AAGCGTAAGCGGCAAAACTT
SMU.423	TTGAGGGTGGTGGTATGATTAG	CCAACGACTGGGAGAGTAACTG
SMU.574	GCTCTCCCTATTGCTCACGCTATC	CCTAAGCCAACACCGATTTCACTC
SMU.610	TTTGCCGATGAAACGACCAC	TACTCGCACTCCCTTGAGCCTC
SMU.704	CAGTTCCGCCAAGATAAGAAG	AGACAAACAGCTAATGCAGCC
SMU.825	ATGGTCACGTCCAGTGCAAG	TCGTCTTTGCCCAAGGATAC
SMU.921	GCCATGGTGTTGAACATCTG	AATGAGATTACTGGTCACAGAT
SMU.1004	TTACTGATGACATGGTGGCC	GCTTGAATAGTTGCAGCTGC
SMU.1114	ATTGTTGCTCGGGCTCTTCCA	ATGCGGCTTGTCAGGAGTAACC
SMU.1246	CGCTGAAGGAGGAGATTTTC	CGACGACTCGTTTGATTAAGG
SMU.1396	CGCATTTCTCCTTTTCTTGCCG	ACAACTCCTGATGAACCAACGC
SMU.1519	GACAGGTTCTTCTTTTACTTG	GGTCCTTAGTTGAAGCATTGG
SMU.1700	AGTTCCACCAAGAGCCAAACC	AGATGCAGGGAATTACGACCG
SMU.1877	CATCAAAGAAGCCAAAGACG	CCATCACCAATGACAGTGCC
SMU.1915	GACTTTAAAGAAATTAAGACTG	AAGCTTGTGTAAAACTTCTGT
SMU.2061	GCGAAAGCTCCTCAAGCAAC	AACTTTGGCATCTCCTGTAGC
SMU.2147	CACCTGATTCTTTTTGAGCG	CGGAACCAACTGAGACCAAG

Table S2. DEGs significantly up-regulated ($log_2FC \geq 2$) in the persister population. *S. mutans* gene locus tag as in GenBank (UA159 accession no. AE014133).

**Gene ID**	**Log_2FC**	**Product**
SMU.40	6.10	Conserved hypothetical protein
SMU.1072c	5.38	Putative acetyltransferase
SMU.1827	5.21	Putative biotin biosynthesis protein
SMU.1396	5.14	Glucan-binding protein C, GpcC
SMU.1489	4.90	Conserved hypothetical protein
SMU.265	4.81	Putative carbamate kinase
SMU.263	4.64	Putative amino acid antiporter
SMU.1488c	4.49	Conserved hypothetical protein
SMU.264	4.43	Conserved hypothetical protein
SMU.42	4.34	Conserved hypothetical protein
SMU.124	4.27	Putative transcriptional regulator (MarR family)
SMU.262	4.21	Putative ornithine carbamoyltransferase
SMU.1063	4.15	Putative ABC transporter, ATP-binding protein, proline/glycine betaine transport system
SMU.1494	4.12	Tagatose-6-phosphate kinase
SMU.1884c	4.08	Hypothetical protein
SMU.1491	4.08	PTS system, lactose-specific enzyme IIBC
SMU.1490	4.05	6-phospho-beta-galactosidase
SMU.706c	4.04	Conserved hypothetical protein
SMU.1493	3.95	Tagatose-1,6-bisphosphate aldolase
SMU.125	3.95	Conserved hypothetical protein
SMU.1492	3.92	PTS system, lactose-specific enzyme IIA
SMU.1495	3.88	Galactose-6-phosphate isomerase, subunit LacB
SMU.1149	3.85	Putative transporter, trans-membrane domain bacteriocin immunity protein
SMU.1150	3.52	Hypothetical protein
SMU.260	3.49	Conserved hypothetical protein
SMU.1062	3.49	Putative ABC transporter, proline/glycine betaine permease protein
SMU.1357	3.44	Putative transposase fragment
SMU.1284c	3.39	Conserved hypothetical protein
SMU.1496	3.37	Galactose-6-phosphate isomerase, subunit LacA
SMU.531	3.37	Putative chorismate mutase
SMU.1297	3.33	3′-phosphoadenosine phosphatase
SMU.2116	3.31	Putative osmoprotectant amino acid ABC transporter, ATP-binding protein
SMU.1148	3.28	Putative transporter, ATP-binding protein; bacteriocin immunity protein
SMU.1420	3.17	Putative oxidoreductase
SMU.2133c	3.15	Putative membrane protein
SMU.1286c	3.09	Putative permease; multidrug efflux protein

SMU.417	3.08	Conserved hypothetical protein
SMU.1419	3.08	Putative transcriptional regulator
SMU.668c	3.06	Ribonucleotide reductase, large subunit
SMU.658	2.95	Conserved hypothetical protein
SMU.2146c	2.92	Hypothetical protein
SMU.2117	2.91	Putative osmoprotectant ABC transporter; permease protein
SMU.1437	2.84	Putative UDP-N-acetylglucosamine 2-epimerase
SMU.1175	2.82	Putative sodium/amino acid (alanine) symporter
SMU.1237c	2.75	Hypothetical protein
SMU.418	2.74	Putative transcription factor NusA
SMU.1649	2.70	Putative exodeoxyribonuclease III
SMU.2147c	2.68	Conserved hypothetical protein
SMU.1553c	2.65	Hypothetical protein
SMU.667	2.65	Putative ribonucleotide reductase, small subunit
SMU.561c	2.65	Putative hydrolase (MutT family)
SMU.953c	2.64	Putative transcriptional regulator/aminotransferase
SMU.562	2.58	ATP-dependent protease ClpE
SMU.921	2.57	Putative transcriptional regulator
SMU.1436c	2.56	Hypothetical protein
SMU.924	2.55	Thiol peroxidase
SMU.1259	2.54	Conserved hypothetical protein; possible restriction endonuclease
SMU.299c	2.48	Putative bacteriocin peptide precursor
SMU.1028	2.47	Putative hydrolase or acyltransferase
SMU.2109	2.46	Putative MDR permease, possible multidrug efflux pump
SMU.71	2.42	Putative cation efflux pump (multidrug resistance protein)
SMU.1036	2.40	Hypothetical protein
SMU.956	2.39	Putative Clp-like ATP-dependent protease, ATP-binding subunit
SMU.1969c	2.38	Putative transcriptional regulator
SMU.419	2.37	Conserved hypothetical protein
SMU.1795c	2.33	Conserved hypothetical protein
SMU.1554c	2.32	Hypothetical protein
SMU.29	2.31	Putative phosphoribosylaminoimidazole-succinocarboxamide synthase SAICAR synthetase
SMU.63c	2.29	Conserved hypothetical protein
SMU.216c	2.29	Hypothetical protein
SMU.1280c	2.26	Conserved hypothetical protein
SMU.922	2.26	Putative ABC transporter, ATP-binding protein
SMU.1915	2.26	Competence-stimulating peptide, precursor
SMU.1519	2.25	Putative amino acid ABC transporter, ATP-binding protein
SMU.2072c	2.25	Conserved hypothetical protein; possible acetyltransferase
SMU.611	2.25	Putative ATP-dependent RNA helicase, DEAD-box family
SMU.1621c	2.25	Conserved hypothetical protein
SMU.420	2.23	Putative ribosomal protein
SMU.279	2.22	Hypothetical protein

SMU.1515	2.22	Conserved hypothetical protein CovX (VicX)
SMU.638	2.22	Putative 16S pseudouridylate synthase
SMU.1236c	2.21	Conserved hypothetical protein
SMU.1790c	2.20	Putative transcriptional regulator
SMU.28	2.19	Putative ATP-binding protein
SMU.270	2.19	Putative PTS system, membrane component; possible ribulose-monophosphate PTS pathway enzyme IIC
SMU.704c	2.19	Putative autolysin, amidase
SMU.524	2.19	Putative ABC transporter, ATP-binding protein
SMU.1791c	2.18	Conserved hypothetical protein
SMU.426	2.17	Copper-transporting ATPase; P-type ATPase
SMU.2118	2.16	Putative ABC transporter; osmoprotectant-binding protein, glycine betaine/carnitine/choline ABC transporter
SMU.211c	2.16	Hypothetical protein
SMU.1027	2.15	Putative transcriptional regulator
SMU.1434c	2.13	Putative glycosyltransferase
SMU.1517	2.13	Putative response regulator CovR (VicR-like protein)
SMU.1761c	2.12	Conserved hypothetical protein
SMU.290	2.11	Conserved hypothetical protein
SMU.657	2.11	Putative MutG
SMU.629	2.10	Putative manganese-type superoxide dismutase, Fe/Mn-SOD
SMU.1438c	2.09	Putative Zn-dependent protease
SMU.1794c	2.09	Conserved hypothetical protein
SMU.407	2.09	Conserved hypothetical protein
SMU.401c	2.08	Conserved hypothetical protein
SMU.1753c	2.08	Conserved hypothetical protein
SMU.532	2.06	Putative anthranilate synthase, alpha subunit
SMU.1035	2.06	Putative ABC transporter, ATP-binding protein
SMU.48	2.06	Putative phosphoribosylamine-glycine ligase; phosphoribosyl glycinamide synthetase (GARS)
SMU.278	2.05	Hypothetical protein
SMU.610	2.05	Cell surface antigen I/II
SMU.1435c	2.04	Hypothetical protein
SMU.383c	2.04	Conserved hypothetical protein, putative reductase
SMU.703c	2.03	Conserved hypothetical protein, possible membrane protein
SMU.1657c	2.03	Putative nitrogen regulatory protein PII
SMU.1432c	2.01	Putative endoglucanase precursor
SMU.1703c	2.01	Conserved hypothetical protein
SMU.1865	2.00	Putative A/G-specific DNA glycosylase
SMU.427	2.00	Putative copper chaperone
SMU.1117	2.00	H_2O-forming NADH oxidase
SMU.1516	2.00	Putative histidine kinase CovS (VicK-like protein)

Table S3. DEGs significantly down-regulated ($log_2FC \leq -2$) in the persister population. *S. mutans* gene locus tag as in GenBank (UA159 accession no. AE014133).

Gene ID	Log_2FC	Product
SMU.1001	–5.20	Putative DNA processing Smf protein
SMU.625	–5.09	Putative competence protein
SMU.1956c	–4.99	Hypothetical protein
SMU.1960c	–4.96	Putative PTS system, mannose-specific IIB component
SMU.836	–4.94	Hypothetical protein
SMU.1983	–4.79	Putative competence protein ComYD
SMU.1967	–4.74	Putative single-stranded DNA -binding protein
SMU.1961c	–4.68	Putative PTS system, sugar-specific enzyme IIA component
SMU.1906c	–4.65	Hypothetical protein
SMU.1984	–4.64	Putative competence protein ComYC
SMU.1905c	–4.63	Putative bacteriocin secretion protein
SMU.1908c	–4.61	Hypothetical protein
SMU.1958c	–4.57	Putative PTS system, mannose-specific IIC component
SMU.1909c	–4.57	Hypothetical protein
SMU.1957	–4.54	Putative PTS system, mannose-specific IID component
SMU.1981c	–4.53	Conserved hypothetical protein
SMU.626	–4.50	Putative competence protein
SMU.1902c	–4.42	Hypothetical protein
SMU.1980c	–4.42	Conserved hypothetical protein
SMU.877	–4.39	Alpha-galactosidase
SMU.1982c	–4.38	Conserved hypothetical protein
SMU.1979c	–4.34	Conserved hypothetical protein
SMU.1985	–4.32	Putative ABC transporter ComYB; probably part of the DNA transport machinery
SMU.1402c	–4.31	Conserved hypothetical protein
SMU.1904c	–4.29	Hypothetical protein
SMU.1910c	–4.29	Hypothetical protein
SMU.539c	–4.20	Signal peptidase type IV
SMU.1347c	–4.17	Conserved hypothetical protein; possible permease
SMU.837	–4.07	Putative reductase
SMU.1404c	–4.07	Conserved hypothetical protein
SMU.1913c	–4.05	Putative immunity protein, BlpL-like
SMU.1405c	–4.00	Conserved hypothetical protein
SMU.769	–3.95	Conserved hypothetical protein
SMU.1987	–3.94	Putative ABC transporter, ATP-binding protein ComYA; late competence gene
SMU.113	–3.89	Putative fructose-1-phosphate kinase
SMU.878	–3.88	Multiple sugar-binding ABC transporter, sugar-binding protein precursor MsmE
SMU.1403c	–3.77	Conserved hypothetical protein
SMU.115	–3.72	Putative PTS system, fructose-specific IIA component
SMU.498	–3.64	Putative late competence protein

SMU.499	–3.59	Putative late competence protein
SMU.879	–3.57	Multiple sugar-binding ABC transporter, permease protein MsmF
SMU.1334	–3.56	Putative phosphopantetheinyl transferase
SMU.151	–3.55	Hypothetical protein
SMU.1568	–3.54	Putative maltose/maltodextrin ABC transporter, sugar-binding protein MalX
SMU.114	–3.47	Putative PTS system, fructose-specific IIBC component
SMU.1978	–3.44	Putative acetate kinase
SMU.1336	–3.41	Conserved hypothetical protein PksD, involved in polyketide synthesis
SMU.1002	–3.39	Putative DNA topoisomerase I
SMU.506	–3.37	Putative type II restriction endonuclease
SMU.116	–3.34	Tagatose 1,6-aldolase
SMU.500	–3.33	Putative ribosome-associated protein
SMU.772	–3.31	Glucan-binding protein D with lipase activity; BglB-like protein
SMU.505	–3.23	Putative adenine-specific DNA methylase
SMU.1055	–3.21	Putative DNA repair protein RadC
SMU.1365c	–3.18	Hypothetical protein; possible permease
SMU.503c	–3.15	Hypothetical protein
SMU.609	–3.14	Putative 40K cell wall protein precursor
SMU.152	–3.14	Hypothetical protein
SMU.1337c	–3.13	Putative alpha/beta superfamily hydrolase
SMU.1340	–3.11	Putative surfactin synthetase
SMU.881	–3.09	Sucrose phosphorylase, GtfA
SMU.1003	–3.08	Putative glucose-inhibited division protein
SMU.1338c	–3.07	Putative permease; possible multidrug-efflux transporter
SMU.1339	–3.06	Putative bacitracin synthetase
SMU.154	–3.05	30S ribosomal protein S15
SMU.1335c	–3.05	Putative enoyl-(acyl-carrier-protein) reductase
SMU.840c	–3.02	Hypothetical protein
SMU.1398	–2.99	Putative transcriptional regulator
SMU.984	–2.99	Hypothetical protein
SMU.1780	–2.97	Conserved hypothetical protein
SMU.575c	–2.93	Putative membrane protein
SMU.150	–2.87	Hypothetical protein
SMU.391c	–2.82	Conserved hypothetical protein
SMU.880	–2.81	Multiple sugar-binding ABC transporter, permease protein MsmG
SMU.108	–2.81	Hypothetical protein
SMU.1529	–2.76	FoF1 membrane-bound proton-translocating ATPase, gamma subunit
SMU.1042	–2.74	Conserved hypothetical protein; inner membrane protein
SMU.574c	–2.69	Putative membrane protein
SMU.1361c	–2.64	Putative transcriptional regulator (TetR family)
SMU.1534	–2.63	FoF1 membrane-bound proton-translocating ATPase, c subunit
SMU.1914c	–2.63	Hypothetical protein

SMU.238c	–2.61	Putative ABC transporter, ATP-binding protein
SMU.219	–2.58	Hypothetical protein
SMU.186	–2.58	Putative metal-dependent transcriptional regulator
SMU.1528	–2.56	FoF1 membrane-bound proton-translocating ATPase, beta subunit
SMU.1088	–2.56	Putative thiamine biosynthesis lipoprotein
SMU.1531	–2.54	FoF1 membrane-bound proton-translocating ATPase, delta subunit
SMU.886	–2.53	Galactokinase, GalK
SMU.1900	–2.53	Conserved hypothetical protein
SMU.1341c	–2.51	Putative gramicidin S synthetase
SMU.889	–2.48	Putative penicillin-binding protein, class C; fmt-like protein
SMU.1424	–2.47	Putative dihydrolipoamide dehydrogenase
SMU.986c	–2.45	Hypothetical protein
SMU.423	–2.44	Hypothetical protein
SMU.936	–2.43	Putative amino acid ABC transporter, ATP-binding protein
SMU.1533	–2.43	FoF1 membrane-bound proton-translocating ATPase, a subunit
SMU.1530	–2.41	FoF1 membrane-bound proton-translocating ATPase, alpha subunit
SMU.1090	–2.35	Conserved hypothetical protein
SMU.1575c	–2.35	Hypothetical protein
SMU.2127	–2.33	Putative succinate semialdehyde dehydrogenase
SMU.1070c	–2.32	Conserved hypothetical protein
SMU.1152c	–2.32	Conserved hypothetical protein
SMU.1160c	–2.32	Hypothetical protein
SMU.1019	–2.31	Putative citrate lyase, gamma-subunit
SMU.1532	–2.31	FoF1 membrane-bound proton-translocating ATPase, b subunit
SMU.1342	–2.31	Putative bacitracin synthetase 1; BacA
SMU.1125c	–2.31	Conserved hypothetical protein
SMU.184	–2.29	Putative ABC transporter, metal binding lipoprotein; surface adhesin precursor; saliva-binding protein; lipoprotein receptor LraI (LraI family)
SMU.888	–2.28	UDP-galactose 4-epimerase, GalE
SMU.237c	–2.27	Putative integral membrane protein
SMU.1293c	–2.27	Conserved hypothetical protein
SMU.1023	–2.27	Putative pyruvate carboxylase/oxaloacetate decarboxylase, alpha subunit
SMU.1343c	–2.26	Putative polyketide synthase
SMU.1367c	–2.26	Conserved hypothetical protein
SMU.1569	–2.25	Putative maltose/maltodextrin ABC transporter, permease protein MalF
SMU.78	–2.24	Fructan hydrolase; exp-beta-D-fructosidase; fructanase, FruA
SMU.1021	–2.24	Putative citrate lyase, alpha subunit
SMU.1126	–2.21	Putative pantothenate kinase
SMU.1564	–2.21	Putative glycogen phosphorylase
SMU.1570	–2.21	Putative maltose/maltodextrin ABC transporter, MalG permease
SMU.1124	–2.19	Putative pyrimidine-nucleoside phosphorylase

SMU.1017	–2.18	Putative oxaloacetate decarboxylase, sodium ion pump subunit
SMU.1153c	–2.18	Hypothetical protein
SMU.883	–2.17	Dextran glucosidase DexB
SMU.1016	–2.17	Putative acetyl-CoA carboxylase, biotin carboxyl carrier subunit
SMU.1067c	–2.16	Putative ABC transporter, permease protein
SMU.1344c	–2.15	Putative malonyl-CoA acyl-carrier-protein transacylase
SMU.1423	–2.15	Putative pyruvate dehydrogenase, TPP-dependent E1 component alpha-subunit
SMU.110	–2.12	Putative transcriptional regulator MutR
SMU.1022	–2.12	Conserved hypothetical protein, CitG-like protein
SMU.1089	–2.11	Conserved hypothetical protein
SMU.1843	–2.11	Sucrose-6-phosphate hydrolase
SMU.2086	–2.11	Putative competence and damage inducible protein CinA
SMU.882	–2.10	Multiple sugar-binding ABC transporter, ATP-binding protein, MsmK
SMU.352	–2.08	Putative ribulose-phosphate-3-epimerase
SMU.1562	–2.08	Putative potassium uptake protein TrkA
SMU.1725	–2.07	Putative acylphosphatase
SMU.1509	–2.06	Putative transcriptional regulator
SMU.804	–2.05	Hypothetical protein
SMU.236c	–2.04	Putative transcriptional regulator
SMU.182	–2.03	Putative ABC transporter, ATP-binding protein; possible iron and/or manganese ABC transport system
SMU.1041	–2.02	Conserved hypothetical protein; inner membrane protein
SMU.1069c	–2.02	Hypothetical protein
SMU.934	–2.02	Putative amino acid ABC transporter, permease protein
SMU.1652	–2.02	Putative methylated-DNA-protein-cysteine-S-methyltransferase
SMU.1538	–2.02	Putative glucose-1-phosphate adenylyltransferase; ADP-glucose pyrophosphorylase
SMU.1527	–2.01	FoF1 membrane-bound proton-translocating ATPase, epsilon subunit
SMU.935	–2.01	Putative amino acid ABC transporter, permease protein

Table S4. Complete RNA-seq data. CSP-induced persisters vs. CSP-induced normal stationary cells.
Log2FC: Log2(fold-change); padj: adjusted *p* -value

**Locus**	**Coordinates**	**Gene biotype**	**Log2FC**	**padj**
SMU.01	NC_004350.2:194-1552	protein_coding	0.506896414	1.5649E-05
SMU.02	NC_004350.2:1708-2844	protein_coding	0.234655414	0.048053706
SMU.05	NC_004350.2:3106-3297	protein_coding	0.094768804	0.677719422
SMU.06	NC_004350.2:3461-4576	protein_coding	1.219955399	2.14287E-17
SMU.07	NC_004350.2:4663-5232	protein_coding	0.525365857	0.002423787
SMU.08	NC_004350.2:5225-8722	protein_coding	-0.066798002	0.610544656
SMU.09	NC_004350.2:8852-9124	protein_coding	-0.688936079	0.000567312
SMU.10	NC_004350.2:9111-9482	protein_coding	0.829399102	0.009598545
SMU.11	NC_004350.2:9482-9601	protein_coding	0.653913204	0.037076806
SMU.12	NC_004350.2:9611-10846	protein_coding	-0.377248509	0.032277387
SMU.13	NC_004350.2:10843-12114	protein_coding	-0.626948684	1.43675E-06
SMU.14	NC_004350.2:12118-12660	protein_coding	1.151203326	9.5319E-16
SMU.15	NC_004350.2:12682-14652	protein_coding	1.444726037	4.84871E-62
SMU.16	NC_004350.2:15140-16555	protein_coding	1.881246307	3.223E-28
SMU_r01	NC_004350.2:16870-18426	rRNA	-1.428382196	0.61278516
SMU_t01	NC_004350.2:18486-18558	tRNA	NA	NA
SMU_r02	NC_004350.2:18815-21715	rRNA	-0.406069788	0.18656709
SMU_r03	NC_004350.2:21898-22013	rRNA	NA	NA
SMU_t02	NC_004350.2:22018-22090	tRNA	NA	NA
SMU_t03	NC_004350.2:22120-22192	tRNA	NA	NA
SMU_t04	NC_004350.2:22220-22292	tRNA	0	1
SMU_t05	NC_004350.2:22302-22383	tRNA	NA	NA
SMU_t06	NC_004350.2:22398-22470	tRNA	0	1
SMU_t07	NC_004350.2:22473-22544	tRNA	NA	NA
SMU_t08	NC_004350.2:22552-22637	tRNA	-0.997655193	0.209439724
SMU_t09	NC_004350.2:22651-22724	tRNA	2.895446292	0.014365303
SMU_t10	NC_004350.2:22778-22851	tRNA	-1.42837471	0.762676718
SMU_t11	NC_004350.2:22857-22930	tRNA	2.300468776	0.002778814
SMU_t12	NC_004350.2:22954-23027	tRNA	2.359466003	0.363739402
SMU_t13	NC_004350.2:23042-23131	tRNA	0.897665366	0.617208004
SMU_t14	NC_004350.2:23144-23217	tRNA	NA	NA
SMU_t15	NC_004350.2:23227-23299	tRNA	-0.468642642	0.786805024
SMU_t16	NC_004350.2:23306-23376	tRNA	3.391819067	0.037584987
SMU_t17	NC_004350.2:23411-23484	tRNA	NA	NA
SMU_t18	NC_004350.2:23494-23581	tRNA	4.489471836	0.000632234
SMU.20	NC_004350.2:23646-24464	protein_coding	-0.174138234	0.399001361
SMU.21	NC_004350.2:24466-24972	protein_coding	-0.667156285	0.256383213
SMU.22	NC_004350.2:25082-26377	protein_coding	0.953292123	4.49382E-27
SMU.23	NC_004350.2:26502-27470	protein_coding	0.314667525	0.028845002
SMU.24	NC_004350.2:27559-28734	protein_coding	-0.719232816	4.10732E-07
SMU.25	NC_004350.2:28724-29479	protein_coding	-1.014410645	1.54899E-14
SMU.26	NC_004350.2:29687-30685	protein_coding	-0.121672493	0.42995767
SMU.27	NC_004350.2:30687-30935	protein_coding	0.486720809	0.245851465
SMU.28	NC_004350.2:31136-31744	protein_coding	2.194602103	0.00028616
SMU.29	NC_004350.2:31867-32574	protein_coding	2.310525143	1.15025E-12
SMU.30	NC_004350.2:32584-36309	protein_coding	1.392738699	4.95234E-24
SMU.31	NC_004350.2:36331-36951	protein_coding	0.876836794	0.000394114
SMU.32	NC_004350.2:36977-38416	protein_coding	1.243331917	1.92866E-20
NA	NC_004350.2:38429-38914	protein_coding	1.029412615	3.99288E-06
SMU.34	NC_004350.2:38960-39982	protein_coding	1.227149208	2.14978E-13
SMU.35	NC_004350.2:39982-40536	protein_coding	0.959937787	1.71778E-08
SMU.36	NC_004350.2:40643-41350	protein_coding	1.096280026	1.75293E-13
SMU.37	NC_004350.2:41392-42939	protein_coding	1.376050354	1.10136E-23
SMU.38c	NC_004350.2:43122-43664	protein_coding	1.14896425	0.00017129
SMU.39	NC_004350.2:43832-44341	protein_coding	1.101871596	0.000905985

SMU.40	NC_004350.2:44518-44679	protein_coding	6.104168439	3.24398E-08
SMU.42	NC_004350.2:44831-45709	protein_coding	4.342853329	3.08473E-57
SMU.43	NC_004350.2:45781-47160	protein_coding	-0.997494565	3.67499E-08
SMU.45	NC_004350.2:47171-49570	pseudogene	-1.224871695	1.07956E-14
SMU.46	NC_004350.2:49572-50207	protein_coding	-0.894602477	3.0943E-06
SMU.47	NC_004350.2:50207-50815	protein_coding	-1.059086339	4.1841E-08
SMU.48	NC_004350.2:51039-52298	protein_coding	2.059910766	1.50414E-61
SMU.49	NC_004350.2:52358-53539	protein_coding	1.727532449	2.21644E-46
SMU.50	NC_004350.2:53850-54350	protein_coding	1.601802783	6.95179E-27
SMU.51	NC_004350.2:54337-55428	protein_coding	1.702026752	6.76209E-51
SMU.52	NC_004350.2:55428-56093	protein_coding	1.268692204	1.88248E-18
SMU.53	NC_004350.2:56109-56328	pseudogene	1.487712352	8.02469E-09
SMU.54	NC_004350.2:56331-57023	protein_coding	1.737178359	7.06394E-23
SMU.55	NC_004350.2:57125-57388	protein_coding	1.275015102	2.55254E-05
NA	NC_004350.2:57408-57633	pseudogene	1.662892732	0.01007959
SMU.58	NC_004350.2:57639-58934	protein_coding	1.862868834	3.04842E-50
SMU.59	NC_004350.2:59244-60542	protein_coding	1.027672586	1.25807E-14
SMU.60	NC_004350.2:60562-61248	protein_coding	-1.006999693	8.35671E-08
SMU.61	NC_004350.2:61631-62545	protein_coding	-0.06853813	0.692862725
SMU.63c	NC_004350.2:63079-64920	protein_coding	2.291213335	2.5372E-103
SMU.64	NC_004350.2:65256-66251	protein_coding	-0.657922935	3.25138E-07
SMU.65	NC_004350.2:66289-66714	protein_coding	-0.361999928	0.043742455
SMU.66	NC_004350.2:66779-67162	protein_coding	-0.802593982	1.75911E-09
SMU.67	NC_004350.2:67159-68949	protein_coding	-1.112633769	1.44929E-38
SMU.70	NC_004350.2:69631-71115	protein_coding	0.684590447	7.13219E-08
SMU.71	NC_004350.2:71571-72872	protein_coding	2.420568229	2.27909E-25
SMU.72	NC_004350.2:73231-73497	protein_coding	1.577650476	9.18828E-07
SMU.73	NC_004350.2:73517-74854	protein_coding	1.367637239	1.2528E-26
SMU.74	NC_004350.2:74927-75637	protein_coding	-0.039901145	0.856076894
SMU.75	NC_004350.2:75621-76370	protein_coding	0.218507275	0.072463917
SMU.76	NC_004350.2:76374-76961	protein_coding	0.135095897	0.494437927
SMU.78	NC_004350.2:77576-81847	protein_coding	-2.241070253	1.2551E-109
SMU.79	NC_004350.2:81918-83477	protein_coding	-1.457632591	4.98022E-19
SMU.80	NC_004350.2:83652-84686	protein_coding	0.650012375	2.13084E-10
SMU.81	NC_004350.2:84729-85268	protein_coding	0.490919911	1.86451E-05
SMU.82	NC_004350.2:85642-87480	protein_coding	0.219172228	0.025287386
SMU.83	NC_004350.2:88008-89141	protein_coding	0.859657339	6.32471E-19
SMU.84	NC_004350.2:89443-90192	protein_coding	0.336280915	0.005252546
SMU.85	NC_004350.2:90182-90949	protein_coding	-0.076062407	0.601265684
SMU.86	NC_004350.2:90936-91400	protein_coding	-0.434234092	0.008896365
SMU.87	NC_004350.2:91445-92008	protein_coding	-0.378306682	0.008362264
SMU.88c	NC_004350.2:92179-93036	protein_coding	-0.764015703	5.14384E-05
SMU.89c	NC_004350.2:93254-94063	protein_coding	-1.057474806	0.00077476
SMU.91	NC_004350.2:94301-95584	protein_coding	0.502878495	0.00013247
SMU.96	NC_004350.2:97086-97670	protein_coding	-0.073269066	0.73483946
SMU.97	NC_004350.2:97804-99414	protein_coding	0.829397876	5.17527E-14
SMU_t19	NC_004350.2:100332-100417	tRNA	0.863412627	0.417254855
SMU.99	NC_004350.2:100574-101455	protein_coding	-1.435450971	2.64285E-54
SMU.100	NC_004350.2:101780-102271	protein_coding	-1.080214207	3.83854E-05
SMU.101	NC_004350.2:102284-103081	protein_coding	-1.166416572	4.61905E-11
SMU.102	NC_004350.2:103071-103892	protein_coding	-1.44192889	1.74669E-28
SMU.103	NC_004350.2:103889-104305	protein_coding	-1.390718636	2.9315E-15
SMU.104	NC_004350.2:104305-106500	protein_coding	-1.613302613	1.1902E-42
SMU.105	NC_004350.2:106501-107499	protein_coding	-1.435193443	1.02811E-29
NA	NC_004350.2:107595-108316	pseudogene	1.862004168	0.003639956
SMU_t20	NC_004350.2:108558-108630	tRNA	2.906532263	0.05311045
NA	NC_004350.2:108744-108809	pseudogene	2.417322935	0.239205263
SMU.109	NC_004350.2:109582-110682	protein_coding	-1.716755212	1.29341E-20
SMU.110	NC_004350.2:110824-111684	protein_coding	-2.116147919	1.07294E-28

SMU.112c	NC_004350.2:111945-112694	protein_coding	-0.138941604	0.444714757
SMU.113	NC_004350.2:112823-113755	protein_coding	-3.889525459	2.6205E-91
SMU.114	NC_004350.2:113760-115160	protein_coding	-3.468219145	2.2187E-175
SMU.115	NC_004350.2:115175-115627	protein_coding	-3.719617768	1.16534E-71
SMU.116	NC_004350.2:115645-116634	protein_coding	-3.33651666	1.2483E-118
SMU.117c	NC_004350.2:116735-118090	protein_coding	1.018315036	7.57426E-05
SMU.118c	NC_004350.2:118705-119547	protein_coding	0.909207952	4.36096E-10
SMU.119	NC_004350.2:119558-120676	protein_coding	1.393682113	3.94587E-15
SMU.120	NC_004350.2:121103-121291	protein_coding	0.959035633	0.012615389
SMU.121	NC_004350.2:121344-122672	protein_coding	1.663208295	1.3907E-07
SMU.123	NC_004350.2:122905-127302	protein_coding	0.057268803	0.584680127
SMU.124	NC_004350.2:127526-127954	protein_coding	4.273784616	2.0742E-237
SMU.125	NC_004350.2:128033-128587	protein_coding	3.949166798	1.1E-221
SMU.127	NC_004350.2:128963-129958	protein_coding	1.46787258	1.87823E-27
SMU.128	NC_004350.2:130108-131103	protein_coding	1.320809664	1.40155E-16
SMU.129	NC_004350.2:131203-132570	protein_coding	1.030785028	2.11657E-16
SMU.130	NC_004350.2:132644-134389	protein_coding	1.026983537	3.47226E-17
SMU.131	NC_004350.2:134495-135484	protein_coding	0.86321659	3.90295E-13
SMU.132	NC_004350.2:135749-136885	protein_coding	1.341135825	1.08998E-24
SMU.133c	NC_004350.2:137008-138402	protein_coding	-0.128617379	0.66386045
SMU.134	NC_004350.2:138583-139206	protein_coding	0.579346155	0.114518575
SMU.135	NC_004350.2:139471-140364	protein_coding	-0.234556697	0.255078947
SMU.136c	NC_004350.2:140554-140907	protein_coding	-1.083443067	1.22239E-06
SMU.137	NC_004350.2:141022-142644	protein_coding	-1.027057404	1.40183E-22
SMU.138	NC_004350.2:142800-143744	protein_coding	-0.693751731	1.85616E-06
SMU.139	NC_004350.2:143865-145049	protein_coding	-0.69456944	4.87905E-08
SMU.140	NC_004350.2:145067-146428	protein_coding	-0.735525133	1.17222E-12
SMU.141	NC_004350.2:146412-147131	protein_coding	-0.688623831	3.50639E-11
SMU.143c	NC_004350.2:147295-147909	protein_coding	0.951881266	7.84913E-14
SMU.144c	NC_004350.2:147984-148631	protein_coding	1.288131997	6.51331E-18
SMU.145	NC_004350.2:148736-149920	protein_coding	1.772768953	4.25364E-16
SMU.148	NC_004350.2:150120-152789	protein_coding	-1.669951175	6.16031E-88
NA	NC_004350.2:152964-153432	pseudogene	-2.588839594	4.82974E-97
SMU.150	NC_004350.2:153813-154016	protein_coding	-2.86569488	1.35682E-53
SMU.151	NC_004350.2:154046-154261	protein_coding	-3.554245697	6.0815E-116
SMU.152	NC_004350.2:154456-154875	protein_coding	-3.140729357	6.2198E-116
NA	NC_004350.2:155527-155835	protein_coding	-3.431341938	4.9486E-156
SMU.154	NC_004350.2:156212-156481	protein_coding	-3.046461081	1.0773E-137
SMU.155	NC_004350.2:157228-159420	protein_coding	0.629741842	3.19569E-08
SMU.156	NC_004350.2:159410-160150	protein_coding	0.014431038	0.938292359
SMU.157	NC_004350.2:160161-160778	protein_coding	0.211007435	0.160577877
SMU.158	NC_004350.2:160852-162195	protein_coding	-0.229057022	0.105714708
SMU.159	NC_004350.2:162188-162607	protein_coding	-0.354742211	0.050819815
SMU.160	NC_004350.2:162786-163691	protein_coding	-0.399206919	0.005388837
SMU.161	NC_004350.2:163670-164407	protein_coding	-0.377082995	0.024620174
SMU.162c	NC_004350.2:164443-165435	protein_coding	1.358303608	2.52636E-18
SMU.163c	NC_004350.2:165624-166178	protein_coding	0.995502064	2.22778E-09
SMU.164	NC_004350.2:166491-167237	protein_coding	0.586620493	0.002054837
SMU.165	NC_004350.2:167261-168121	protein_coding	0.995856691	3.29132E-07
SMU.166	NC_004350.2:168253-168768	protein_coding	-1.096479489	7.6497E-10
SMU.167	NC_004350.2:168768-169214	protein_coding	-1.126235363	7.61958E-08
SMU.168	NC_004350.2:169211-169405	protein_coding	-1.150117519	5.08339E-08
SMU.169	NC_004350.2:169798-170244	protein_coding	1.008921451	5.24928E-16
SMU.170	NC_004350.2:170269-170661	protein_coding	1.28291396	1.39824E-32
SMU.172	NC_004350.2:170983-171228	protein_coding	-0.190701075	0.388486301
SMU.173	NC_004350.2:171222-171554	protein_coding	0.349390264	0.133404926
SMU.174c	NC_004350.2:171752-172543	protein_coding	-0.831088367	8.58015E-06
NA	NC_004350.2:172710-173063	pseudogene	2.56505891	3.20586E-08
SMU.177	NC_004350.2:173245-174465	protein_coding	1.444992434	3.51451E-20

SMU.178	NC_004350.2:174443-175291	protein_coding	0.963581314	6.03715E-08
SMU.179	NC_004350.2:175433-176035	protein_coding	-1.586893662	5.18354E-56
SMU.180	NC_004350.2:176037-178448	protein_coding	-1.706302606	6.81471E-95
SMU.181	NC_004350.2:178654-179652	protein_coding	-0.312375567	0.047774545
SMU.182	NC_004350.2:179874-180596	protein_coding	-2.027936932	2.56016E-31
SMU.183	NC_004350.2:180593-181432	protein_coding	-1.968590473	5.93379E-74
SMU.184	NC_004350.2:181479-182399	protein_coding	-2.289174897	2.0988E-148
SMU.186	NC_004350.2:182584-183237	protein_coding	-2.582699611	7.69E-166
SMU.187c	NC_004350.2:183417-184394	protein_coding	1.453271303	9.88667E-33
SMU.188c	NC_004350.2:184381-185253	protein_coding	1.776443692	2.78432E-23
SMU_r04	NC_004350.2:185742-187298	rRNA	-1.318858669	0.6965518
SMU_t21	NC_004350.2:187358-187430	tRNA	NA	NA
SMU_r05	NC_004350.2:187687-190587	rRNA	1.995433231	0.395020455
SMU_r06	NC_004350.2:190770-190885	rRNA	1.969893315	0.255078947
SMU_t22	NC_004350.2:190890-190962	tRNA	0.49520461	0.891783044
SMU_t23	NC_004350.2:190968-191038	tRNA	3.38699621	0.0160618
SMU_t24	NC_004350.2:191073-191146	tRNA	NA	NA
SMU_t25	NC_004350.2:191158-191229	tRNA	0	1
SMU_t26	NC_004350.2:191244-191333	tRNA	2.44471347	0.46629333
SMU_t27	NC_004350.2:191346-191419	tRNA	NA	NA
SMU_t28	NC_004350.2:191429-191501	tRNA	0.120768938	0.922599346
SMU_t29	NC_004350.2:191517-191597	tRNA	1.937849073	0.469737111
SMU_t30	NC_004350.2:191604-191674	tRNA	1.99724313	0.191402156
SMU_t31	NC_004350.2:191688-191760	tRNA	1.997054042	0.60175791
SMU_t32	NC_004350.2:191777-191848	tRNA	2.759071897	0.372803191
SMU_t33	NC_004350.2:191863-191946	tRNA	4.300713768	0.004633157
SMU.191c	NC_004350.2:192029-193183	protein_coding	-0.791922431	0.031754055
SMU.193c	NC_004350.2:193199-193384	protein_coding	-0.571962571	0.370291671
SMU.194c	NC_004350.2:193388-193639	protein_coding	-0.813777154	0.056590357
SMU.195c	NC_004350.2:193675-193935	protein_coding	-0.256563414	0.448447098
SMU.196c	NC_004350.2:193953-195050	protein_coding	-0.038221764	0.837270863
SMU.197c	NC_004350.2:195072-197498	protein_coding	-0.515259425	0.001190714
SMU.198c	NC_004350.2:197498-200041	protein_coding	-0.563253392	1.17703E-05
SMU.199c	NC_004350.2:200061-200441	protein_coding	-0.694443488	0.043424439
SMU.200c	NC_004350.2:200470-200697	protein_coding	0.080968517	0.751730618
SMU.201c	NC_004350.2:200710-201690	protein_coding	-0.188292867	0.292728707
SMU.202c	NC_004350.2:201706-202179	protein_coding	0.399113347	0.030688646
SMU.205c	NC_004350.2:202493-202765	protein_coding	0.601869475	0.0389054
SMU.206c	NC_004350.2:202868-203269	protein_coding	0.48491193	0.03715331
SMU.207c	NC_004350.2:203232-204464	protein_coding	-0.024735227	0.888920618
SMU.208c	NC_004350.2:204699-206423	protein_coding	0.048107213	0.736544301
SMU.209c	NC_004350.2:206439-206864	protein_coding	0.42080914	0.05760911
SMU.210c	NC_004350.2:206888-207199	protein_coding	0.373748909	0.089255778
SMU.211c	NC_004350.2:207419-207862	protein_coding	2.158987508	2.16118E-25
SMU.213c	NC_004350.2:208136-208327	protein_coding	1.051963949	3.17151E-06
SMU.214c	NC_004350.2:208355-208663	protein_coding	1.991654335	2.08356E-09
SMU.215c	NC_004350.2:208672-208815	protein_coding	1.731854182	3.45411E-05
SMU.216c	NC_004350.2:208952-209290	protein_coding	2.288752217	4.14496E-09
SMU.217c	NC_004350.2:209386-209913	protein_coding	1.512581799	2.12997E-07
SMU.218	NC_004350.2:210660-211013	protein_coding	-1.71206058	4.89192E-06
SMU.219	NC_004350.2:211020-211451	protein_coding	-2.577124717	1.37657E-15
NA	NC_004350.2:211577-211675	protein_coding	-2.46573093	2.03811E-36
SMU.220c	NC_004350.2:211770-212222	protein_coding	0.865016714	1.07106E-06
SMU.221c	NC_004350.2:212568-213197	protein_coding	0.176623331	0.243128681
SMU.222c	NC_004350.2:213510-213704	protein_coding	0.259487696	0.364006351
NA	NC_004350.2:214544-215438	pseudogene	0.387155725	0.00014944
SMU.227c	NC_004350.2:215841-216404	protein_coding	-0.058411629	0.718020653
SMU.228	NC_004350.2:216789-217154	protein_coding	0.727728616	0.000319134
SMU.229	NC_004350.2:217154-218821	protein_coding	0.358363821	0.000635529

SMU.231	NC_004350.2:218987-220690	protein_coding	0.82861192	5.39569E-10
SMU.232	NC_004350.2:220683-221165	protein_coding	0.606483819	0.000246951
SMU.233	NC_004350.2:221232-222254	protein_coding	-0.109173269	0.428454359
SMU.234	NC_004350.2:222495-223745	protein_coding	1.888275809	3.68808E-42
SMU.235	NC_004350.2:223800-224687	protein_coding	0.625694239	7.71968E-08
SMU.236c	NC_004350.2:224810-225451	protein_coding	-2.035944797	6.53187E-51
SMU.237c	NC_004350.2:225477-226700	protein_coding	-2.266334392	3.01322E-63
SMU.238c	NC_004350.2:226697-227416	protein_coding	-2.607332163	4.61652E-45
SMU.239c	NC_004350.2:227612-228202	protein_coding	0.773510258	0.098260257
SMU.241c	NC_004350.2:228505-229245	protein_coding	-0.69102392	1.94011E-05
SMU.242c	NC_004350.2:229245-230798	protein_coding	-0.651814399	1.82885E-05
SMU.243	NC_004350.2:230950-232851	protein_coding	0.45308446	0.000825651
SMU.244	NC_004350.2:232909-233754	protein_coding	0.274526805	0.163938955
SMU.245	NC_004350.2:233844-234566	protein_coding	0.560003187	0.000136038
SMU.246	NC_004350.2:234572-235732	protein_coding	0.751356704	1.45675E-10
SMU.247	NC_004350.2:235806-236576	protein_coding	0.787151049	6.34292E-08
SMU.248	NC_004350.2:236614-237876	protein_coding	0.2395582	0.028627668
SMU.249	NC_004350.2:237881-239110	protein_coding	0.214275387	0.039888497
SMU.250	NC_004350.2:239097-239531	protein_coding	0.298953884	0.107136793
SMU.251	NC_004350.2:239552-240967	protein_coding	0.047177841	0.674892486
SMU.252	NC_004350.2:241259-242032	protein_coding	-0.924547719	8.02932E-06
SMU.253	NC_004350.2:242290-243531	protein_coding	-0.425178847	0.010275068
SMU.255	NC_004350.2:243822-245471	protein_coding	-0.740883123	1.05857E-09
SMU.256	NC_004350.2:245577-246491	protein_coding	-0.23640413	0.127368608
SMU.257	NC_004350.2:246502-247533	protein_coding	-0.154132994	0.172229603
SMU.258	NC_004350.2:247543-248595	protein_coding	-0.490823679	1.31717E-06
SMU.259	NC_004350.2:248588-249514	protein_coding	-0.866902789	7.16442E-20
SMU.260	NC_004350.2:250170-250772	protein_coding	3.489294591	7.63348E-58
SMU.261c	NC_004350.2:250989-251945	protein_coding	0.722636409	0.027187942
SMU.262	NC_004350.2:252184-253203	protein_coding	4.213255166	5.41971E-22
SMU.263	NC_004350.2:253301-254659	protein_coding	4.641897739	1.30204E-25
SMU.264	NC_004350.2:254669-255778	protein_coding	4.430695246	3.63085E-15
SMU.265	NC_004350.2:255810-256760	protein_coding	4.809396574	8.28908E-13
SMU.267c	NC_004350.2:257272-259575	protein_coding	-0.315892904	0.021831674
SMU.268	NC_004350.2:259734-261023	protein_coding	-0.651678242	7.35736E-09
SMU.270	NC_004350.2:261355-262812	protein_coding	2.190982286	0.008623087
SMU.271	NC_004350.2:262880-263161	protein_coding	1.137439421	0.024817523
SMU.272	NC_004350.2:263227-263712	protein_coding	1.860473303	0.000101028
SMU.273	NC_004350.2:263751-264416	protein_coding	1.420525737	0.006094872
SMU.274	NC_004350.2:264422-265285	protein_coding	1.716524279	0.003334421
SMU.275	NC_004350.2:265288-265998	protein_coding	1.787170862	0.001851397
SMU.277	NC_004350.2:266388-266663	protein_coding	1.184818028	0.006608111
SMU.278	NC_004350.2:266877-267038	protein_coding	2.054061804	4.89307E-06
SMU.279	NC_004350.2:267523-267732	protein_coding	2.224257343	0.010805075
SMU.281	NC_004350.2:267970-268248	protein_coding	1.837842039	0.00085704
NA	NC_004350.2:268857-269042	protein_coding	1.354783184	0.001912726
SMU.283	NC_004350.2:269323-269541	protein_coding	1.04415323	0.027692004
SMU.284	NC_004350.2:269566-269973	protein_coding	0.869908651	0.03382275
SMU.286	NC_004350.2:270628-272910	protein_coding	0.404883139	0.000277144
SMU.287	NC_004350.2:272924-273826	protein_coding	0.078964164	0.543976929
SMU.289	NC_004350.2:274183-275850	protein_coding	-1.005534211	6.60629E-13
SMU.290	NC_004350.2:276429-277520	protein_coding	2.108891425	0.012591942
SMU.291	NC_004350.2:277894-279870	protein_coding	0.541104235	0.299793874
SMU.292	NC_004350.2:280430-281398	protein_coding	0.266548552	0.370551823
SMU.293	NC_004350.2:281529-282041	protein_coding	0.921980954	0.004805941
SMU.294	NC_004350.2:282116-282568	protein_coding	0.197725017	0.602754322
SMU.295	NC_004350.2:282568-282951	protein_coding	0.386785706	0.279138478
SMU.296	NC_004350.2:282969-283943	protein_coding	0.218767903	0.45181259
SMU.297	NC_004350.2:284102-286738	protein_coding	0.468223817	4.51948E-05

SMU.298	NC_004350.2:286831-287259	protein_coding	-0.063637304	0.679818246
SMU.299c	NC_004350.2:287452-287670	protein_coding	2.475305763	6.32464E-17
SMU.300	NC_004350.2:288082-289224	protein_coding	0.252489225	0.119368429
SMU.301	NC_004350.2:289224-289787	protein_coding	-0.372867549	0.012085601
SMU.302	NC_004350.2:290214-291227	protein_coding	0.388558887	0.048965433
SMU.303	NC_004350.2:291304-292080	protein_coding	-1.07993594	5.75056E-22
SMU.304	NC_004350.2:292080-292550	protein_coding	-1.204306189	1.53137E-16
SMU.305	NC_004350.2:292547-293110	protein_coding	-1.114779213	4.02279E-18
NA	NC_004350.2:293138-293235	SRP_RNA	2.372709255	0.000501113
SMU.307	NC_004350.2:293667-295016	protein_coding	-1.463828886	1.58046E-59
SMU.308	NC_004350.2:295182-295982	protein_coding	-0.745625421	1.18281E-05
SMU.309	NC_004350.2:296004-297869	protein_coding	-1.243416877	1.63486E-21
SMU.310	NC_004350.2:297869-298357	protein_coding	-1.10321237	3.75932E-08
SMU.311	NC_004350.2:298437-298979	protein_coding	-0.736173394	5.63504E-07
SMU.312	NC_004350.2:299070-300080	protein_coding	-0.762129774	1.02707E-10
SMU.313	NC_004350.2:300122-300487	protein_coding	-0.558207107	0.003166638
SMU.314	NC_004350.2:300696-300899	protein_coding	-0.537448888	0.024587186
SMU.317	NC_004350.2:301122-301820	protein_coding	-0.213060108	0.287759927
SMU.318	NC_004350.2:302577-303707	protein_coding	-0.982727253	6.04663E-16
SMU.320	NC_004350.2:304041-304577	protein_coding	0.742421807	0.004214955
SMU.321	NC_004350.2:304564-305235	protein_coding	0.686253115	0.000377333
SMU.322c	NC_004350.2:305257-306177	protein_coding	0.358708146	0.007391679
SMU.323	NC_004350.2:306179-307198	protein_coding	0.812585785	2.23853E-11
SMU.325	NC_004350.2:307522-307965	protein_coding	-0.481409943	0.028233422
SMU.326	NC_004350.2:307975-308445	protein_coding	-0.297058832	0.222089022
SMU.327	NC_004350.2:308403-309803	protein_coding	-0.052592474	0.680263438
SMU.328	NC_004350.2:309893-310387	protein_coding	0.537877021	9.37661E-06
SMU.329	NC_004350.2:310576-311289	protein_coding	-0.199718234	0.111262223
SMU.330	NC_004350.2:311576-313033	protein_coding	-0.654349149	5.3309E-09
SMU.331	NC_004350.2:313149-313673	protein_coding	-0.806350751	4.22603E-09
SMU.332	NC_004350.2:313740-314288	protein_coding	-0.862589177	5.89199E-10
SMU.333	NC_004350.2:314437-315672	protein_coding	-0.309133443	0.007083039
SMU.334	NC_004350.2:315869-317059	protein_coding	-0.941273217	3.71118E-15
SMU.335	NC_004350.2:317402-318784	protein_coding	0.424080479	0.025637357
SMU.336	NC_004350.2:318879-319238	protein_coding	0.964076733	0.000918951
SMU.337	NC_004350.2:319222-320037	protein_coding	1.055494355	7.6324E-14
SMU.338	NC_004350.2:320050-321018	protein_coding	0.777758966	1.81403E-08
SMU.339	NC_004350.2:321128-321730	protein_coding	0.719342693	6.95295E-08
SMU.340	NC_004350.2:321953-322087	protein_coding	0.25196052	0.122021561
SMU.341	NC_004350.2:322388-323164	protein_coding	1.12548146	1.59636E-12
SMU.342	NC_004350.2:323395-323967	protein_coding	1.39664151	5.73966E-22
SMU.343	NC_004350.2:324244-324765	protein_coding	1.669617758	8.60361E-17
SMU.344	NC_004350.2:324812-325123	protein_coding	1.869621065	1.48694E-20
SMU.345c	NC_004350.2:325128-325442	protein_coding	-0.19035265	0.529587965
SMU.346	NC_004350.2:325543-326157	protein_coding	1.964110666	2.65492E-17
SMU.348	NC_004350.2:326257-326703	protein_coding	1.839224382	1.96244E-24
SMU.349	NC_004350.2:326715-327590	protein_coding	1.501113133	1.06141E-26
SMU.350	NC_004350.2:327600-328004	protein_coding	1.140121229	1.29862E-10
SMU_t34	NC_004350.2:328109-328197	tRNA	1.937764073	0.066564529
SMU_t35	NC_004350.2:328360-328442	tRNA	1.324741117	0.777959254
SMU.351	NC_004350.2:328549-329421	protein_coding	0.121455762	0.3700957
SMU.352	NC_004350.2:329429-330088	protein_coding	-2.076097037	1.76831E-62
SMU.353	NC_004350.2:330081-330713	protein_coding	-1.743942839	5.00585E-42
SMU.354	NC_004350.2:330720-331994	protein_coding	-1.900127309	1.45826E-63
SMU.355	NC_004350.2:331984-332952	protein_coding	-1.697073505	1.03308E-30
SMU.356	NC_004350.2:333020-333835	protein_coding	-1.186375361	3.33373E-22
SMU.357	NC_004350.2:334095-334508	protein_coding	0.849333692	1.01412E-18
SMU.358	NC_004350.2:334526-334996	protein_coding	0.888618157	5.10697E-22
SMU.359	NC_004350.2:335165-337246	protein_coding	0.551394944	8.6214E-08

SMU.360	NC_004350.2:337541-338554	protein_coding	-1.737062814	4.43258E-77
SMU.361	NC_004350.2:338960-340156	protein_coding	-0.412480549	3.02605E-06
SMU.362	NC_004350.2:340543-341061	protein_coding	1.187735399	2.57381E-12
SMU.363	NC_004350.2:341143-341514	protein_coding	-1.153761108	1.37395E-26
SMU.364	NC_004350.2:341554-342900	protein_coding	-0.276408005	0.024432753
SMU.365	NC_004350.2:343060-347577	protein_coding	-0.231640271	0.011044376
SMU.366	NC_004350.2:347579-349015	protein_coding	-0.49239738	3.07171E-06
SMU.367	NC_004350.2:349163-349798	protein_coding	-0.120932464	0.384851073
SMU.368c	NC_004350.2:349984-351666	protein_coding	-0.066931498	0.584680127
SMU.369c	NC_004350.2:351668-351898	protein_coding	0.541955886	0.152762031
SMU.370	NC_004350.2:352360-353205	protein_coding	-0.150194454	0.724244914
SMU.371	NC_004350.2:353198-353911	protein_coding	-0.644024569	0.217603553
SMU.372	NC_004350.2:353956-355119	protein_coding	-0.487409402	0.216017453
SMU.373	NC_004350.2:355112-355855	protein_coding	0.123006604	0.766145256
SMU.374	NC_004350.2:355842-356552	protein_coding	-0.240565185	0.554364093
SMU.375	NC_004350.2:356563-357471	protein_coding	-0.42846859	0.154136761
SMU.376	NC_004350.2:357487-358725	protein_coding	-0.27244179	0.177552204
SMU.378	NC_004350.2:358920-359159	protein_coding	-0.668414847	0.258444824
SMU.381c	NC_004350.2:359781-360683	protein_coding	-0.114555139	0.390101224
SMU.382c	NC_004350.2:361058-362026	protein_coding	1.572620947	6.78245E-06
SMU.383c	NC_004350.2:362047-363093	protein_coding	2.037949238	4.4224E-09
SMU.384	NC_004350.2:363267-363725	protein_coding	-1.317094876	0.005749074
SMU.385	NC_004350.2:363736-364422	protein_coding	-0.652933481	0.006457104
SMU.386	NC_004350.2:364422-364856	protein_coding	-0.938356757	0.000211557
SMU.387	NC_004350.2:364871-365881	protein_coding	-0.904804892	9.25302E-06
SMU.388	NC_004350.2:365960-366658	protein_coding	-0.027063324	0.872394781
SMU.389	NC_004350.2:366648-366971	protein_coding	-0.258486033	0.273574096
SMU.391c	NC_004350.2:367067-367492	protein_coding	-2.822609944	3.87939E-92
SMU.392c	NC_004350.2:367656-368828	protein_coding	0.664532763	0.000671002
SMU.393	NC_004350.2:368963-369478	protein_coding	-0.309203498	0.369951145
SMU.394c	NC_004350.2:369598-369897	protein_coding	-0.611126287	0.001783874
SMU.395	NC_004350.2:370156-372432	protein_coding	-0.085607145	0.552793094
SMU.396	NC_004350.2:372547-373416	protein_coding	1.768526091	7.50385E-41
SMU.399	NC_004350.2:373588-374346	protein_coding	-1.231269462	2.22455E-13
SMU.400	NC_004350.2:374343-375293	protein_coding	-1.364807276	1.59214E-27
SMU.401c	NC_004350.2:375427-375867	protein_coding	2.078276073	5.43288E-15
SMU.402	NC_004350.2:376029-378356	protein_coding	-1.082645902	2.63602E-23
SMU.403	NC_004350.2:378579-379691	protein_coding	1.256747118	6.54255E-15
SMU.404c	NC_004350.2:379697-380020	protein_coding	-0.341192302	0.12810913
SMU.405c	NC_004350.2:380033-380440	protein_coding	-1.031733116	0.000766691
SMU.406c	NC_004350.2:380555-381367	protein_coding	0.697008235	0.001485942
SMU.407	NC_004350.2:381676-382275	protein_coding	2.09037214	1.30266E-10
SMU.408	NC_004350.2:382345-383775	protein_coding	1.33326546	3.67284E-26
SMU.409	NC_004350.2:383904-384347	protein_coding	0.634406861	0.001967957
SMU.410	NC_004350.2:384678-385898	protein_coding	1.204604166	1.64926E-19
SMU.411c	NC_004350.2:386068-386361	protein_coding	-1.29551541	2.84754E-15
SMU.412c	NC_004350.2:386358-386777	protein_coding	-1.284107195	1.0753E-07
SMU.413	NC_004350.2:386842-387567	protein_coding	0.004745395	0.981686625
SMU.414	NC_004350.2:387570-388604	protein_coding	0.074305759	0.601265684
SMU.415	NC_004350.2:388663-389445	protein_coding	-0.11364073	0.381629637
SMU.416	NC_004350.2:389455-390090	protein_coding	-0.23811439	0.185076714
SMU_t36	NC_004350.2:390169-390255	tRNA	1.548662671	0.390730866
SMU.417	NC_004350.2:390362-390853	protein_coding	3.083867319	4.35023E-80
SMU.418	NC_004350.2:390889-392082	protein_coding	2.740270049	2.4751E-126
SMU.419	NC_004350.2:392100-392396	protein_coding	2.368956449	4.22939E-27
SMU.420	NC_004350.2:392389-392691	protein_coding	2.226992494	1.19048E-39
SMU.421	NC_004350.2:392712-395462	protein_coding	1.721150489	5.77179E-79
SMU.422	NC_004350.2:395696-396046	protein_coding	1.48988263	3.6963E-26
SMU.423	NC_004350.2:396531-396761	protein_coding	-2.436633452	1.39301E-59

SMU.424	NC_004350.2:397152-397595	protein_coding	1.524160511	1.02527E-19
SMU.426	NC_004350.2:397592-399820	protein_coding	2.167570678	4.10664E-62
SMU.427	NC_004350.2:399833-400036	protein_coding	2.00083434	9.56827E-36
SMU.428	NC_004350.2:400277-401104	protein_coding	1.102171397	1.37845E-11
SMU.429c	NC_004350.2:401211-401702	protein_coding	1.162573965	0.00462378
SMU.431	NC_004350.2:402057-402923	protein_coding	1.330350062	1.25964E-08
SMU.432	NC_004350.2:402924-403781	protein_coding	0.838237383	0.000233298
SMU.433	NC_004350.2:403795-404253	protein_coding	0.451695961	0.029398735
SMU.434	NC_004350.2:404267-404665	protein_coding	0.451151109	0.080847635
SMU.435	NC_004350.2:404987-406165	protein_coding	-0.171624309	0.195867198
NA	NC_004350.2:406250-407599	protein_coding	0.87469015	0.695551195
SMU.438c	NC_004350.2:407739-412040	protein_coding	-1.893949369	2.78005E-80
SMU.439	NC_004350.2:412321-412932	protein_coding	0.373205494	0.17728829
SMU.440	NC_004350.2:413049-413465	protein_coding	0.037733	0.830946301
SMU.441	NC_004350.2:413462-413920	protein_coding	0.123843938	0.443823419
SMU.442	NC_004350.2:413913-414323	protein_coding	0.134594673	0.299982159
SMU.445	NC_004350.2:414774-415691	protein_coding	1.09098811	1.39133E-13
SMU.446	NC_004350.2:415694-417733	protein_coding	-0.212663475	0.036193902
SMU.447	NC_004350.2:417839-418093	protein_coding	-0.221531115	0.152762031
SMU.448	NC_004350.2:418217-418597	protein_coding	-1.184590838	3.23532E-21
SMU.449	NC_004350.2:419095-419895	protein_coding	0.460813082	0.005950819
SMU.450	NC_004350.2:419899-421149	protein_coding	0.066286077	0.631528412
SMU.453	NC_004350.2:421452-422402	protein_coding	0.890540414	1.53144E-08
SMU.454	NC_004350.2:422412-422735	protein_coding	0.788257648	0.001839735
SMU.455	NC_004350.2:422739-424988	protein_coding	0.369750557	0.000755624
SMU.456	NC_004350.2:424990-426009	protein_coding	0.084585329	0.598311681
SMU.457	NC_004350.2:426043-426270	protein_coding	0.220644949	0.73467152
SMU.458	NC_004350.2:426390-427733	protein_coding	0.88910224	4.07677E-11
SMU.459	NC_004350.2:427974-428795	protein_coding	-0.304734896	0.055694397
SMU.460	NC_004350.2:428818-429621	protein_coding	-0.8851677	1.08812E-08
SMU.461	NC_004350.2:429621-430364	protein_coding	-1.14502122	1.42304E-09
SMU.462	NC_004350.2:430413-430637	protein_coding	-0.035609511	0.957855277
SMU.463	NC_004350.2:430700-431614	protein_coding	1.824650401	2.43545E-35
SMU.464	NC_004350.2:432078-433538	protein_coding	0.596012242	1.97646E-07
SMU.465	NC_004350.2:433535-434359	protein_coding	-0.079198083	0.543976929
SMU.466	NC_004350.2:434472-435806	protein_coding	0.702023995	2.33704E-09
SMU.467	NC_004350.2:435922-438153	protein_coding	0.903557397	2.22123E-21
SMU.469	NC_004350.2:438146-438739	protein_coding	1.712571364	3.17682E-38
SMU.470	NC_004350.2:438819-439340	protein_coding	-0.480028981	0.110011144
SMU.471	NC_004350.2:439446-439784	protein_coding	-0.202597907	0.151854345
NA	NC_004350.2:439802-440176	RNase_P_RNA	-3.341660459	1.9124E-101
SMU.472	NC_004350.2:440234-441388	protein_coding	0.963637037	9.58895E-15
SMU.473	NC_004350.2:441404-443044	protein_coding	0.180702498	0.103372567
SMU.474	NC_004350.2:443205-443687	protein_coding	-0.200350236	0.315633178
SMU.475	NC_004350.2:443832-445439	protein_coding	1.788754969	5.08803E-58
SMU.478	NC_004350.2:445886-446518	protein_coding	0.580760672	2.47915E-06
SMU.479	NC_004350.2:446542-446859	protein_coding	0.76144333	0.000216987
SMU.480	NC_004350.2:446901-449285	protein_coding	0.739126465	4.84871E-13
SMU.481	NC_004350.2:449345-450280	protein_coding	0.408104992	0.003003632
SMU.482	NC_004350.2:450261-451583	protein_coding	0.192577015	0.171332825
SMU.483	NC_004350.2:451622-452365	protein_coding	0.365737954	0.004808695
SMU.484	NC_004350.2:452362-454212	protein_coding	-0.081499354	0.438234701
SMU.485	NC_004350.2:454653-455348	protein_coding	0.353913266	0.27889517
SMU.486	NC_004350.2:455345-456349	protein_coding	0.48313626	0.023586193
SMU.487	NC_004350.2:456342-456983	protein_coding	0.005411205	0.982733009
SMU.488	NC_004350.2:457027-458442	protein_coding	0.469062272	0.001273232
SMU.489	NC_004350.2:458426-458812	protein_coding	-0.330779958	0.200089787
SMU.490	NC_004350.2:458891-459667	protein_coding	-1.281974047	8.57877E-14
SMU.491	NC_004350.2:459798-460544	protein_coding	1.734472673	2.7539E-13

SMU.493	NC_004350.2:460793-463249	protein_coding	-0.054348291	0.736544301
SMU.494	NC_004350.2:463268-463936	protein_coding	-0.178444189	0.251210729
SMU.495	NC_004350.2:463974-465065	protein_coding	-0.556512199	3.70862E-05
SMU.496	NC_004350.2:465399-466325	protein_coding	-1.949987142	4.20172E-38
SMU.497c	NC_004350.2:466416-467045	protein_coding	-1.352208658	3.91325E-05
SMU.498	NC_004350.2:467118-468419	protein_coding	-3.635874074	1.6442E-116
SMU.499	NC_004350.2:468416-469081	protein_coding	-3.589109386	1.7022E-112
SMU.500	NC_004350.2:469160-469708	protein_coding	-3.334763862	2.7367E-114
SMU.501	NC_004350.2:469832-470926	protein_coding	0.35313007	0.068446462
SMU.502	NC_004350.2:470946-472646	protein_coding	0.252923759	0.047459576
SMU.503c	NC_004350.2:472726-473361	protein_coding	-3.146234821	1.2689E-100
SMU.504	NC_004350.2:473630-474487	protein_coding	-0.891741839	0.000377762
SMU.505	NC_004350.2:474477-475286	protein_coding	-3.232342433	2.7713E-103
SMU.506	NC_004350.2:475270-476208	protein_coding	-3.367386429	4.2272E-117
NA	NC_004350.2:476294-476476	protein_coding	-3.527818467	7.6397E-48
SMU.507	NC_004350.2:476732-477478	protein_coding	-1.050187601	2.89707E-05
SMU.508	NC_004350.2:477490-478314	protein_coding	-1.297359376	3.82934E-11
SMU.509	NC_004350.2:478383-479447	protein_coding	-0.074612741	0.807257093
SMU.510c	NC_004350.2:479522-480157	protein_coding	-1.546296507	2.69934E-50
SMU.512c	NC_004350.2:480288-480641	pseudogene	0.326719229	0.575693939
NA	NC_004350.2:480892-481095	protein_coding	-0.485339442	0.201686054
SMU.513	NC_004350.2:481136-481369	protein_coding	-0.280373969	0.477042571
SMU.514	NC_004350.2:481590-482156	protein_coding	-0.295692006	0.20972863
SMU.515	NC_004350.2:482277-484052	protein_coding	-0.314107255	0.157755027
SMU.516	NC_004350.2:484636-485175	protein_coding	1.115586758	0.000229872
SMU.517	NC_004350.2:485165-485665	protein_coding	0.826048913	6.31921E-06
SMU.518	NC_004350.2:485643-486683	protein_coding	0.538461885	0.000791727
SMU.520	NC_004350.2:486756-488087	protein_coding	-0.196605533	0.279235752
SMU.521	NC_004350.2:488084-488632	protein_coding	0.12612892	0.590671586
SMU.522	NC_004350.2:488629-489714	protein_coding	0.093488601	0.477092614
SMU.523	NC_004350.2:489714-490250	protein_coding	0.108030641	0.553302756
SMU.524	NC_004350.2:490389-492152	protein_coding	2.18831644	1.96069E-33
SMU.525	NC_004350.2:492142-493884	protein_coding	1.657340186	3.86616E-23
SMU.526c	NC_004350.2:493951-494787	protein_coding	-1.372575027	1.30204E-25
SMU.527	NC_004350.2:495084-496118	protein_coding	1.728534676	9.66954E-24
SMU.528c	NC_004350.2:496257-496538	protein_coding	-0.918280322	5.92728E-06
SMU.530c	NC_004350.2:496725-497462	protein_coding	-0.989867078	3.15584E-11
SMU.531	NC_004350.2:498149-498445	protein_coding	3.368663602	0.001645697
SMU.532	NC_004350.2:498454-499815	protein_coding	2.062567225	3.34534E-24
SMU.533	NC_004350.2:499812-500375	protein_coding	1.177583695	3.6181E-08
SMU.534	NC_004350.2:500396-501403	protein_coding	1.248524987	3.62438E-10
SMU.535	NC_004350.2:501396-502163	protein_coding	0.839776076	0.00066044
SMU.536	NC_004350.2:502150-502731	protein_coding	1.050999667	0.000419068
SMU.537	NC_004350.2:502728-503939	protein_coding	1.01380677	1.99427E-08
SMU.538	NC_004350.2:503943-504725	protein_coding	0.452557235	0.034848361
SMU.539c	NC_004350.2:504962-505618	protein_coding	-4.204981231	1.44294E-78
SMU.540	NC_004350.2:505719-506246	protein_coding	-0.423582014	0.004607756
SMU.541	NC_004350.2:506453-506659	protein_coding	0.412579757	0.347628489
SMU.542	NC_004350.2:506652-507623	protein_coding	-0.601845396	1.88531E-07
SMU.543	NC_004350.2:507633-508022	protein_coding	-0.798784918	1.06417E-08
SMU.546	NC_004350.2:508256-510100	protein_coding	0.670375868	2.49488E-11
SMU.547	NC_004350.2:510129-510383	protein_coding	0.617130186	0.001793477
SMU.548	NC_004350.2:510572-511927	protein_coding	-0.836689884	1.7952E-18
SMU.549	NC_004350.2:511930-513015	protein_coding	-1.439587663	3.37543E-45
SMU.550	NC_004350.2:513015-514139	protein_coding	-1.856633973	3.06614E-57
SMU.551	NC_004350.2:514291-515652	protein_coding	-0.56465486	1.72394E-08
SMU.552	NC_004350.2:515676-516980	protein_coding	-0.526541996	6.67179E-08
SMU.553	NC_004350.2:516983-517654	protein_coding	-0.674331711	2.85504E-06
SMU.554	NC_004350.2:517668-518243	protein_coding	-0.773016947	1.9727E-13

SMU.555	NC_004350.2:518243-518503	protein_coding	-0.976306182	9.72096E-07
SMU.556	NC_004350.2:518507-519298	protein_coding	-1.003680119	2.96147E-22
SMU.557	NC_004350.2:519309-520124	protein_coding	-0.879606568	8.05978E-17
SMU.558	NC_004350.2:520634-523426	protein_coding	-1.250755573	8.43506E-35
SMU.560c	NC_004350.2:524199-524501	protein_coding	-0.328485217	0.011560288
SMU.561c	NC_004350.2:524568-525023	protein_coding	2.646210977	1.82872E-79
SMU.562	NC_004350.2:525134-527395	protein_coding	2.584027187	4.0551E-186
SMU.563	NC_004350.2:527652-528632	protein_coding	0.499253998	0.064854801
SMU.564	NC_004350.2:528709-528939	protein_coding	-0.373427992	0.139196329
NA	NC_004350.2:529007-530355	protein_coding	1.950456055	0.615382071
SMU.567	NC_004350.2:530592-531278	protein_coding	0.516584827	0.000456418
SMU.568	NC_004350.2:531278-532012	protein_coding	0.610399816	8.27793E-05
SMU.569	NC_004350.2:532205-532681	protein_coding	-0.513279456	0.009421754
SMU.570	NC_004350.2:532678-534828	protein_coding	-0.49808422	5.97734E-07
SMU.571	NC_004350.2:534847-534993	protein_coding	-1.006991945	0.076556179
SMU.572	NC_004350.2:535113-535967	protein_coding	0.764361258	5.17726E-05
SMU.573	NC_004350.2:535964-536797	protein_coding	1.598509135	2.10037E-16
SMU.574c	NC_004350.2:537123-537854	protein_coding	-2.687560574	2.77789E-69
SMU.575c	NC_004350.2:537856-538323	protein_coding	-2.92990418	9.3994E-120
SMU.576	NC_004350.2:538499-539233	protein_coding	-1.083857722	1.43703E-08
SMU.577	NC_004350.2:539214-540956	protein_coding	-1.143301223	2.35594E-19
SMU.580	NC_004350.2:541399-542742	protein_coding	0.315112079	0.019978837
SMU.581	NC_004350.2:542717-542938	protein_coding	0.396578044	0.18049205
SMU.582	NC_004350.2:542935-543804	protein_coding	0.318902562	0.030024578
SMU.583	NC_004350.2:543797-544624	protein_coding	0.146347158	0.346483774
SMU.584	NC_004350.2:544611-545075	protein_coding	-0.29510267	0.111262223
SMU.585	NC_004350.2:545092-546750	protein_coding	-0.488059699	0.000620178
SMU.586	NC_004350.2:546972-547811	protein_coding	-0.39056354	0.002865169
SMU.587	NC_004350.2:547804-548661	protein_coding	-0.76094304	4.02343E-13
SMU.588	NC_004350.2:548636-549235	protein_coding	-0.86812786	3.28424E-12
SMU.589	NC_004350.2:549332-549607	protein_coding	-0.359553753	0.005409851
SMU.590c	NC_004350.2:549699-549860	protein_coding	0.20394075	0.722522017
SMU.591c	NC_004350.2:550027-551145	protein_coding	0.138611516	0.590304435
SMU.592c	NC_004350.2:551152-551793	protein_coding	0.677375027	0.034817646
SMU.593	NC_004350.2:552056-552532	protein_coding	0.857665203	4.65185E-06
SMU.595	NC_004350.2:552813-553748	protein_coding	1.125052944	3.74277E-07
SMU.596	NC_004350.2:553966-554658	protein_coding	-0.52407811	1.81917E-06
SMU.597	NC_004350.2:554905-556983	protein_coding	-0.906595224	1.92602E-21
SMU.598	NC_004350.2:556993-557592	protein_coding	-1.327277164	2.45343E-27
SMU.599	NC_004350.2:557804-558853	protein_coding	0.205021868	0.184404131
SMU.600c	NC_004350.2:559025-559585	protein_coding	1.146493041	1.09533E-07
SMU.602	NC_004350.2:559938-560888	protein_coding	1.202483717	2.92678E-09
SMU.603	NC_004350.2:560966-562324	protein_coding	0.358423799	0.011073809
SMU.605	NC_004350.2:562431-563263	pseudogene	0.491566576	0.02686923
SMU.606	NC_004350.2:563276-564523	protein_coding	0.12653182	0.442864879
SMU.607	NC_004350.2:564579-565262	protein_coding	0.178944556	0.359717299
SMU.608	NC_004350.2:565446-566990	protein_coding	0.636261818	1.45043E-05
SMU.609	NC_004350.2:567244-569079	protein_coding	-3.137502714	7.2667E-279
SMU.610	NC_004350.2:569317-574005	protein_coding	2.051855465	3.6425E-123
SMU.611	NC_004350.2:574295-575848	protein_coding	2.245242893	1.9286E-82
SMU.613	NC_004350.2:576338-576616	protein_coding	1.799272601	4.24346E-13
NA	NC_004350.2:576802-577071	protein_coding	1.87143729	5.38775E-10
SMU.616	NC_004350.2:577901-578149	protein_coding	0.520360827	0.043113752
SMU.618	NC_004350.2:578193-578552	protein_coding	-0.978521144	3.51266E-18
SMU.621c	NC_004350.2:579567-580586	protein_coding	-0.764180689	9.99369E-10
SMU.622c	NC_004350.2:580632-581144	protein_coding	-1.259156931	2.55587E-05
SMU.623c	NC_004350.2:581233-582168	protein_coding	-1.329891272	4.11191E-11
SMU.624	NC_004350.2:582307-583059	protein_coding	0.141270682	0.42995767
SMU.625	NC_004350.2:583200-583877	protein_coding	-5.085014382	1.8777E-140

SMU.626	NC_004350.2:583867-586101	protein_coding	-4.50468945	2.1047E-261
SMU.627	NC_004350.2:586287-586913	protein_coding	-1.854793919	1.05937E-12
SMU.628	NC_004350.2:587081-588121	protein_coding	-0.452221978	0.072029265
SMU.629	NC_004350.2:588268-588879	protein_coding	2.102462101	2.30115E-75
SMU.630	NC_004350.2:589431-590486	protein_coding	1.510694261	7.14576E-21
SMU.631	NC_004350.2:590651-591409	protein_coding	-1.078002024	7.0608E-09
SMU.632	NC_004350.2:591478-591942	protein_coding	-0.690961876	0.015681336
SMU.633	NC_004350.2:591989-593218	protein_coding	-1.743117677	2.36744E-57
SMU.634	NC_004350.2:593404-594432	protein_coding	0.904846959	2.86329E-10
SMU.635	NC_004350.2:594570-595289	protein_coding	0.838556591	2.58096E-12
SMU.636	NC_004350.2:595419-596120	protein_coding	0.846309581	1.89189E-12
SMU.638	NC_004350.2:597135-597851	protein_coding	2.217037992	5.65347E-38
SMU.639	NC_004350.2:597866-598366	protein_coding	1.875204725	5.58129E-27
SMU.640c	NC_004350.2:598456-599829	protein_coding	0.119973305	0.7271517
SMU.641	NC_004350.2:599957-600925	protein_coding	1.675448296	1.38384E-27
NA	NC_004350.2:600958-601085	pseudogene	4.109742479	0.009443181
SMU.642	NC_004350.2:601170-601451	protein_coding	1.579049681	3.89007E-10
SMU.643	NC_004350.2:601557-602450	protein_coding	1.107038068	1.33509E-10
SMU.644	NC_004350.2:602758-603696	protein_coding	-0.816551773	9.5083E-06
SMU.645	NC_004350.2:603718-605517	protein_coding	-1.307534016	2.62224E-27
SMU.646	NC_004350.2:605730-606347	protein_coding	-1.049261177	9.77143E-09
SMU.647	NC_004350.2:606397-607104	protein_coding	-0.01774886	0.930597624
SMU.648	NC_004350.2:607166-608167	protein_coding	0.461358364	0.000559115
SMU.649	NC_004350.2:608406-608906	protein_coding	1.074604497	1.40413E-08
SMU.650	NC_004350.2:609121-611739	protein_coding	0.438572165	1.7196E-05
SMU.651c	NC_004350.2:611951-612979	protein_coding	0.124225755	0.681764886
SMU.652c	NC_004350.2:612999-613748	protein_coding	0.617098072	0.099612382
SMU.653c	NC_004350.2:613742-614728	protein_coding	0.898186327	0.003870428
SMU.654	NC_004350.2:614938-615639	protein_coding	1.465685681	0.078174349
SMU.656	NC_004350.2:615641-616389	pseudogene	1.844445135	0.036832191
SMU.657	NC_004350.2:616389-617135	protein_coding	2.105339646	0.000328253
SMU.658	NC_004350.2:617511-618350	protein_coding	2.951037409	2.98869E-05
SMU.659	NC_004350.2:618390-619049	protein_coding	1.008051295	0.049945816
SMU.660	NC_004350.2:619040-620422	protein_coding	0.778376891	0.011951329
SMU.661	NC_004350.2:620728-620973	protein_coding	-1.440067433	4.55266E-06
SMU.662	NC_004350.2:621118-621807	protein_coding	-0.732150661	3.0235E-05
SMU.663	NC_004350.2:621942-622964	protein_coding	-0.408775413	0.415438118
SMU.664	NC_004350.2:623123-624316	protein_coding	0.177839065	0.667079609
SMU.665	NC_004350.2:624345-625082	protein_coding	-0.279924463	0.567423314
SMU.666	NC_004350.2:625218-626357	protein_coding	-0.753470622	0.010737394
SMU.667	NC_004350.2:626481-627440	protein_coding	2.646846919	2.7405E-123
SMU.668c	NC_004350.2:628003-630162	protein_coding	3.06345851	5.3433E-179
SMU.669c	NC_004350.2:630362-630586	protein_coding	1.967817698	6.14288E-34
SMU.670	NC_004350.2:630841-633507	protein_coding	0.938358257	6.24926E-26
SMU.671	NC_004350.2:633512-634630	protein_coding	0.497771893	8.31092E-08
SMU.672	NC_004350.2:634632-635813	protein_coding	0.073802236	0.495359424
SMU.673	NC_004350.2:636481-637278	protein_coding	0.008754033	0.957945292
SMU.674	NC_004350.2:637444-637707	protein_coding	-1.675380657	1.06091E-52
SMU.675	NC_004350.2:637712-639445	protein_coding	-1.400198487	1.75338E-44
SMU.676	NC_004350.2:639675-641102	protein_coding	0.746940174	8.88118E-16
SMU.677	NC_004350.2:641773-642165	protein_coding	0.512953028	0.193461545
SMU.678	NC_004350.2:642165-643007	protein_coding	-0.184706114	0.55578952
SMU.679	NC_004350.2:643017-643883	protein_coding	-0.320822378	0.218915086
SMU.680	NC_004350.2:643925-644263	protein_coding	-0.035008497	0.945260635
SMU.681	NC_004350.2:644674-644859	protein_coding	0.66320619	0.076081306
SMU.682	NC_004350.2:645162-647720	protein_coding	-0.962067336	2.55498E-06
SMU.683	NC_004350.2:647736-651149	protein_coding	-1.152611294	7.83597E-19
SMU.684	NC_004350.2:651153-651842	protein_coding	-0.71907327	0.000598165
SMU.685	NC_004350.2:651839-652240	protein_coding	-1.064824243	6.71213E-08

SMU.688	NC_004350.2:652915-653412	protein_coding	-1.329203533	1.30195E-08
SMU.689	NC_004350.2:653658-656597	protein_coding	0.254221594	0.0402428
SMU.690	NC_004350.2:656618-657205	protein_coding	0.447069869	0.004719275
SMU.691	NC_004350.2:657298-658518	protein_coding	-0.898796964	6.34751E-11
SMU.692	NC_004350.2:658574-659059	protein_coding	-0.839351597	3.13341E-12
SMU.694c	NC_004350.2:659051-659245	protein_coding	0.913517635	0.425936261
SMU.695	NC_004350.2:659291-659773	protein_coding	-0.566154163	1.35031E-05
SMU.696	NC_004350.2:659784-660467	protein_coding	-0.370822865	0.001148385
SMU.697	NC_004350.2:660634-661164	protein_coding	0.238189566	0.06644535
SMU.698	NC_004350.2:661199-661399	protein_coding	0.776062487	3.70852E-08
SMU.699	NC_004350.2:661444-661803	protein_coding	0.55397837	7.90721E-05
SMU.700c	NC_004350.2:662010-662636	protein_coding	0.633490092	3.18843E-06
SMU.701c	NC_004350.2:662644-663012	protein_coding	1.329679905	6.63953E-06
SMU.702c	NC_004350.2:663017-663490	protein_coding	1.358809042	3.78603E-18
SMU.703c	NC_004350.2:663487-664329	protein_coding	2.032758071	1.59762E-43
SMU.704c	NC_004350.2:664373-665356	protein_coding	2.188640759	7.65731E-37
SMU.706c	NC_004350.2:665446-666015	protein_coding	4.042080335	3.91479E-35
SMU.707c	NC_004350.2:666217-667065	protein_coding	1.14714044	1.39754E-15
SMU.709	NC_004350.2:667339-667863	protein_coding	0.257266868	0.309448342
SMU.711	NC_004350.2:667847-668236	protein_coding	1.526062502	0.000100577
SMU.712	NC_004350.2:668354-671077	protein_coding	-1.628919561	8.94623E-72
SMU.713	NC_004350.2:671372-672649	protein_coding	-0.268132044	0.042274383
SMU.714	NC_004350.2:672868-674064	protein_coding	-0.340425521	0.000198482
SMU.715	NC_004350.2:674360-675118	protein_coding	-1.354120774	1.54445E-33
SMU.716	NC_004350.2:675328-676560	protein_coding	-1.323983197	7.43298E-30
SMU.717	NC_004350.2:676563-677777	protein_coding	-1.270223914	1.57317E-31
SMU.718c	NC_004350.2:677777-678586	protein_coding	-1.308685995	4.94368E-17
SMU.719c	NC_004350.2:678834-680138	protein_coding	-1.405038376	1.00351E-27
SMU.720	NC_004350.2:680250-681128	protein_coding	0.23611375	0.298757873
SMU.721	NC_004350.2:681190-681582	protein_coding	-0.148497881	0.545464806
SMU.723	NC_004350.2:681984-684665	protein_coding	0.842117487	3.19077E-13
SMU.724	NC_004350.2:684778-685518	protein_coding	1.120321753	1.57584E-05
SMU.725c	NC_004350.2:685823-686680	protein_coding	0.660033612	0.009324032
SMU.727	NC_004350.2:686922-687305	protein_coding	0.713862371	0.065552195
SMU.728	NC_004350.2:687292-688176	protein_coding	-0.546242779	0.004282852
SMU.730	NC_004350.2:688445-688657	protein_coding	0.45708324	0.69443922
SMU.731	NC_004350.2:688659-689393	protein_coding	0.338160579	0.328042666
SMU.732	NC_004350.2:689393-690043	protein_coding	0.099511288	0.775784737
NA	NC_004350.2:690063-690260	protein_coding	0.540750204	0.452372577
SMU.734	NC_004350.2:690257-690682	protein_coding	0.452472282	0.576170448
SMU.735	NC_004350.2:690868-691317	protein_coding	-0.487690379	0.211567954
SMU.737	NC_004350.2:691383-692591	protein_coding	1.001848328	5.77705E-05
NA	NC_004350.2:692739-693046	pseudogene	0.403995842	0.421362721
SMU.739c	NC_004350.2:693190-694746	protein_coding	0.48516973	0.069156491
SMU.741	NC_004350.2:695056-696114	protein_coding	-0.278228256	0.143480422
SMU.742	NC_004350.2:696178-697008	protein_coding	-0.59544	0.000133533
SMU.743	NC_004350.2:697005-697826	protein_coding	-0.691721994	1.09097E-07
SMU.744	NC_004350.2:697828-699324	protein_coding	-0.546368786	2.48472E-07
SMU.745	NC_004350.2:699401-700765	protein_coding	0.728607058	9.80342E-08
SMU.746c	NC_004350.2:700875-701690	protein_coding	-0.099263786	0.410180174
SMU.747c	NC_004350.2:701687-702592	protein_coding	0.323750533	0.001421742
SMU.751	NC_004350.2:703952-706078	protein_coding	0.47053087	0.000107418
SMU.752	NC_004350.2:706065-706502	protein_coding	0.287616186	0.213800078
SMU.753	NC_004350.2:706590-706889	protein_coding	1.21805636	6.97247E-12
SMU.754	NC_004350.2:707087-708022	protein_coding	0.058339278	0.629944585
SMU.755	NC_004350.2:708015-708794	protein_coding	1.142645927	1.24527E-21
SMU.756	NC_004350.2:708823-709218	protein_coding	1.318018813	1.91329E-15
SMU.757	NC_004350.2:709218-709673	protein_coding	0.857590102	1.15889E-09
SMU.758c	NC_004350.2:709771-710058	protein_coding	-1.606681883	8.53408E-05

SMU.759	NC_004350.2:710298-711224	protein_coding	-0.415394548	0.000392253
SMU.761	NC_004350.2:711496-712782	protein_coding	0.454934426	0.000329935
SMU.764	NC_004350.2:712912-713472	protein_coding	-0.616200605	2.21722E-06
SMU.765	NC_004350.2:713482-715014	protein_coding	-0.399691389	4.48581E-05
NA	NC_004350.2:715085-716433	protein_coding	0.715820793	0.70847189
SMU.768c	NC_004350.2:716480-716797	protein_coding	-1.796517028	1.26094E-19
SMU.769	NC_004350.2:716931-717143	protein_coding	-3.953637678	9.366E-174
SMU.770c	NC_004350.2:717333-718673	protein_coding	-0.759298865	1.94993E-09
SMU.772	NC_004350.2:719789-721969	protein_coding	-3.309707081	5.6185E-178
SMU.773c	NC_004350.2:722354-723844	protein_coding	-0.157323054	0.164720052
SMU.774	NC_004350.2:724249-725151	protein_coding	-1.042417439	4.95862E-14
SMU.775c	NC_004350.2:725457-727610	protein_coding	-0.356058712	0.001818784
SMU.776	NC_004350.2:727743-728900	protein_coding	0.069663998	0.668961154
SMU.777	NC_004350.2:728907-729584	protein_coding	-0.0647926	0.660342648
SMU.778	NC_004350.2:729574-730443	protein_coding	-0.385941511	0.001116463
SMU.779	NC_004350.2:730457-731524	protein_coding	-0.603740334	4.69019E-08
SMU.780	NC_004350.2:731526-732692	protein_coding	-0.752327796	1.59561E-11
SMU.781	NC_004350.2:732756-733862	protein_coding	-0.448508982	1.14326E-06
SMU.782	NC_004350.2:733873-734214	protein_coding	0.184651336	0.27889517
SMU.784	NC_004350.2:734377-735660	protein_coding	-0.591864567	0.000145001
SMU.785	NC_004350.2:735653-736129	protein_coding	-0.618590865	0.004211749
SMU.786	NC_004350.2:736126-736950	protein_coding	-0.627300842	4.51948E-05
SMU.787	NC_004350.2:736995-738383	protein_coding	-0.666638222	5.44034E-09
SMU.788	NC_004350.2:738555-739910	protein_coding	0.876740167	3.48747E-06
SMU.789	NC_004350.2:740430-741065	protein_coding	-0.435076346	0.000827261
SMU.790	NC_004350.2:741646-741885	protein_coding	1.587051258	0.068381494
SMU.793	NC_004350.2:742327-742716	protein_coding	-1.119291602	4.39897E-06
SMU.794	NC_004350.2:742803-743180	protein_coding	-1.481035677	4.04605E-17
SMU.795	NC_004350.2:743214-743621	protein_coding	-1.414043256	1.44076E-15
SMU.796	NC_004350.2:743711-744544	protein_coding	-1.554575502	1.27215E-29
SMU.797	NC_004350.2:744579-744797	protein_coding	-1.916451053	3.64655E-15
SMU.799c	NC_004350.2:745261-745623	protein_coding	-0.753794895	0.00429236
NA	NC_004350.2:745948-746076	protein_coding	-1.685458148	1.22776E-09
SMU.801	NC_004350.2:746130-747440	protein_coding	-0.230073923	0.059685623
SMU.802	NC_004350.2:747742-747924	protein_coding	-0.298727941	0.193976579
SMU.803c	NC_004350.2:747961-749466	protein_coding	1.001168445	9.87016E-06
SMU.804	NC_004350.2:749833-751005	protein_coding	-2.049923738	9.18149E-42
SMU.805c	NC_004350.2:751261-752001	protein_coding	-0.274935941	0.01036737
SMU.806c	NC_004350.2:752001-754187	protein_coding	0.0149437	0.912055891
SMU.807	NC_004350.2:754378-755295	protein_coding	1.669319444	1.65028E-17
SMU.809	NC_004350.2:755492-757483	protein_coding	-1.000589339	2.22282E-19
SMU.811	NC_004350.2:757815-758147	protein_coding	-1.136961287	3.15979E-13
SMU.813	NC_004350.2:758451-759341	protein_coding	-1.379104793	5.13894E-25
SMU.814	NC_004350.2:759399-759863	protein_coding	-1.006302836	3.29479E-09
SMU.815	NC_004350.2:759971-760786	protein_coding	-0.639656332	9.25792E-09
SMU.816	NC_004350.2:761002-762138	protein_coding	0.315082741	0.054040968
SMU.817	NC_004350.2:762171-762986	protein_coding	-0.038127295	0.815155081
SMU.818	NC_004350.2:763198-763398	protein_coding	0.069315041	0.84526967
SMU.819	NC_004350.2:763556-763939	protein_coding	0.682052777	6.60729E-05
SMU.820	NC_004350.2:764099-765619	protein_coding	-0.125829928	0.255503586
SMU.821	NC_004350.2:765793-767550	protein_coding	0.352685706	0.000487107
SMU.822	NC_004350.2:767558-768673	protein_coding	0.533582055	9.87991E-06
SMU.823	NC_004350.2:768686-769021	protein_coding	0.560810447	8.62704E-06
SMU.824	NC_004350.2:769338-770192	protein_coding	0.237887184	0.071948828
SMU.825	NC_004350.2:770299-771453	protein_coding	0.852085472	3.76478E-13
SMU.826	NC_004350.2:771443-772378	protein_coding	0.516835604	3.46335E-06
SMU.827	NC_004350.2:772378-773187	protein_coding	0.736896043	1.30195E-08
SMU.828	NC_004350.2:773187-774404	protein_coding	0.657127607	1.02505E-09
SMU.829	NC_004350.2:774427-775824	protein_coding	0.388438708	0.000359585

SMU.830	NC_004350.2:775821-777572	protein_coding	0.249193856	0.030977976
SMU.831	NC_004350.2:777569-780109	protein_coding	-0.28647052	0.00361296
SMU.832	NC_004350.2:780146-781462	protein_coding	-0.48226076	0.000276823
SMU.833	NC_004350.2:781479-782405	protein_coding	-0.180322791	0.255078947
SMU.834	NC_004350.2:782537-783475	protein_coding	-0.253740779	0.023500114
SMU.835	NC_004350.2:783609-785420	protein_coding	-0.47713304	1.69675E-06
SMU.836	NC_004350.2:785626-787260	protein_coding	-4.939346773	0
SMU.837	NC_004350.2:787379-788221	protein_coding	-4.067397272	4.0854E-246
SMU.838	NC_004350.2:788354-789706	protein_coding	-0.930095469	2.89551E-12
SMU.839	NC_004350.2:789726-790982	protein_coding	0.230821914	0.338755033
SMU.840c	NC_004350.2:791322-791768	protein_coding	-3.024028111	2.52382E-89
SMU.841	NC_004350.2:791892-793034	protein_coding	1.127042414	2.02325E-11
SMU.842	NC_004350.2:793149-794366	protein_coding	1.124601612	9.554E-19
SMU.843	NC_004350.2:794504-795685	protein_coding	-0.980666589	4.01229E-16
SMU.844	NC_004350.2:795716-796246	protein_coding	-0.768030388	1.72818E-07
SMU.845	NC_004350.2:796274-796861	protein_coding	-0.943288622	2.06869E-10
SMU.846	NC_004350.2:797035-797349	protein_coding	-0.262618576	0.048962001
SMU.848	NC_004350.2:797361-797696	protein_coding	0.012995981	0.949347505
SMU.849	NC_004350.2:797718-798011	protein_coding	0.122386011	0.453103346
SMU.850	NC_004350.2:798325-799020	protein_coding	0.634290934	0.056860256
SMU.851	NC_004350.2:799021-799512	protein_coding	0.539872912	0.143611082
SMU.852	NC_004350.2:800165-801070	protein_coding	-0.270309629	0.172402294
SMU.853	NC_004350.2:801079-801540	protein_coding	-0.424337757	0.212213069
SMU.854	NC_004350.2:801530-802420	protein_coding	-0.399991483	0.07437707
SMU.855	NC_004350.2:802622-803194	protein_coding	-0.921874313	1.17425E-08
SMU.856	NC_004350.2:803417-803947	protein_coding	-0.440318348	0.058016489
SMU.857	NC_004350.2:803944-805209	protein_coding	-0.552658451	0.000412943
SMU.858	NC_004350.2:805214-806140	protein_coding	-0.262805839	0.034622871
SMU.859	NC_004350.2:806269-807357	protein_coding	-0.279889891	0.011432449
SMU.860	NC_004350.2:807537-810716	protein_coding	-0.553344783	1.21799E-09
SMU.862	NC_004350.2:811016-812317	protein_coding	0.353976675	0.002117839
SMU.863	NC_004350.2:812323-813030	protein_coding	0.227464742	0.051908147
SMU.864	NC_004350.2:813040-814284	protein_coding	-0.162278876	0.170700223
SMU.865	NC_004350.2:814687-814962	protein_coding	-0.292190582	0.05100379
SMU.866	NC_004350.2:814973-815212	protein_coding	-0.127307853	0.600519188
SMU.867	NC_004350.2:815301-815819	protein_coding	1.915518294	3.57545E-06
SMU.868	NC_004350.2:815809-816531	protein_coding	1.097570713	8.6688E-06
SMU.869	NC_004350.2:816615-817628	protein_coding	0.258155117	0.239910728
SMU.870	NC_004350.2:817781-818494	protein_coding	0.475963278	2.83115E-05
SMU.871	NC_004350.2:818491-819402	protein_coding	0.172756596	0.201616724
SMU.872	NC_004350.2:819399-821366	protein_coding	0.512392493	5.51245E-08
SMU.873	NC_004350.2:821698-823935	protein_coding	0.483252219	0.138191233
SMU.874	NC_004350.2:823953-825809	protein_coding	0.377254227	0.204835377
SMU.875c	NC_004350.2:825849-826640	protein_coding	0.595813462	0.54813616
NA	NC_004350.2:826713-827033	protein_coding	0.403448862	0.892958702
SMU.876	NC_004350.2:827411-828247	protein_coding	0.948658995	0.004888913
SMU.877	NC_004350.2:828355-830517	protein_coding	-4.387582529	0
SMU.878	NC_004350.2:830530-831792	protein_coding	-3.880569945	0
SMU.879	NC_004350.2:831805-832677	protein_coding	-3.57264208	2.0026E-255
SMU.880	NC_004350.2:832692-833525	protein_coding	-2.808717537	1.5441E-187
SMU.881	NC_004350.2:833680-835125	protein_coding	-3.088994205	0
SMU.882	NC_004350.2:835140-836273	protein_coding	-2.103270988	1.0611E-166
SMU.883	NC_004350.2:836364-837974	protein_coding	-2.165413354	2.24937E-97
SMU.885	NC_004350.2:838128-839126	protein_coding	0.567688779	2.40165E-05
SMU.886	NC_004350.2:839269-840441	protein_coding	-2.525791378	3.0061E-140
SMU.887	NC_004350.2:840446-841921	protein_coding	-1.856079382	1.01349E-77
SMU.888	NC_004350.2:842070-843071	protein_coding	-2.277515749	2.0277E-103
SMU.889	NC_004350.2:843339-844418	protein_coding	-2.482112888	1.5666E-146
SMU.890	NC_004350.2:844622-845164	protein_coding	-1.308772312	5.15719E-09

SMU.891	NC_004350.2:845296-846900	protein_coding	-1.148183678	3.5219E-12
SMU.892	NC_004350.2:846893-848704	protein_coding	-1.195504516	1.17427E-19
SMU.893	NC_004350.2:848735-849880	protein_coding	-0.519951185	3.06161E-05
SMU.895	NC_004350.2:849954-850226	protein_coding	0.438093714	0.584680127
SMU.896	NC_004350.2:850213-850491	protein_coding	0.273207924	0.583041704
SMU.897	NC_004350.2:850494-853541	protein_coding	-0.191364143	0.091677634
SMU.898	NC_004350.2:853755-854129	protein_coding	-0.333930926	0.05408176
SMU.899	NC_004350.2:854135-854980	protein_coding	0.092480608	0.465077655
SMU.900	NC_004350.2:854992-855759	protein_coding	0.531383168	0.000674714
SMU.901	NC_004350.2:855756-856961	protein_coding	0.466051092	0.000186582
SMU.902	NC_004350.2:856964-858832	protein_coding	0.113503661	0.327898727
SMU.905	NC_004350.2:859289-861025	protein_coding	0.13530963	0.346030098
SMU.906	NC_004350.2:861036-862808	protein_coding	-0.361148074	0.004888854
SMU.909	NC_004350.2:862924-863844	protein_coding	-0.098933563	0.632249635
SMU.910	NC_004350.2:864064-868452	protein_coding	1.231275522	9.16257E-31
SMU.911c	NC_004350.2:868627-869136	protein_coding	0.382461249	0.080847635
SMU.913	NC_004350.2:869772-871121	protein_coding	0.834252092	2.94385E-07
SMU.914c	NC_004350.2:871233-871616	protein_coding	0.916073572	3.69467E-05
SMU.915c	NC_004350.2:871725-872213	protein_coding	0.465489331	0.031711464
SMU.916c	NC_004350.2:872226-872942	protein_coding	-0.007845268	0.979641793
SMU.917c	NC_004350.2:872939-873385	protein_coding	-0.00399665	0.992444396
SMU.919c	NC_004350.2:873385-874038	protein_coding	0.462029887	0.173016123
SMU.921	NC_004350.2:874590-875024	protein_coding	2.568724066	4.0223E-34
SMU.922	NC_004350.2:875028-876830	protein_coding	2.257561813	4.18039E-68
SMU.923	NC_004350.2:876820-878574	protein_coding	1.14879024	5.41622E-23
SMU.924	NC_004350.2:878771-879256	protein_coding	2.553249589	1.83857E-35
SMU.925	NC_004350.2:879393-879863	protein_coding	-1.130954823	6.7556E-11
SMU.926	NC_004350.2:880091-880714	protein_coding	-0.581979261	0.000423712
SMU.927	NC_004350.2:880715-881401	protein_coding	-0.537263772	0.011951329
SMU.928	NC_004350.2:881407-882639	protein_coding	-0.81573809	8.6214E-08
SMU.929c	NC_004350.2:882695-883045	protein_coding	1.040373408	4.06888E-06
SMU.930c	NC_004350.2:883192-884103	protein_coding	0.522078179	0.10241273
SMU.932	NC_004350.2:884361-885368	protein_coding	-1.919354509	2.58835E-07
SMU.933	NC_004350.2:885392-886246	protein_coding	-1.776804263	1.27208E-11
SMU.934	NC_004350.2:886256-886948	protein_coding	-2.018858476	1.07305E-18
SMU.935	NC_004350.2:886957-887631	protein_coding	-2.008060085	4.91799E-18
SMU.936	NC_004350.2:887641-888405	protein_coding	-2.432455206	3.30486E-19
SMU.937	NC_004350.2:888612-889544	protein_coding	-1.292352235	2.39508E-08
SMU.938	NC_004350.2:889546-890544	protein_coding	-0.936442706	1.36788E-06
SMU.939	NC_004350.2:890554-891549	protein_coding	-1.149902869	1.03699E-09
SMU.940c	NC_004350.2:891632-892288	protein_coding	1.76296881	8.13465E-26
SMU.941c	NC_004350.2:892257-892727	protein_coding	1.930515586	2.22182E-40
SMU.942	NC_004350.2:892870-894153	protein_coding	0.131802609	0.449016401
SMU.943c	NC_004350.2:894131-895306	protein_coding	0.450976039	0.001302861
SMU.944	NC_004350.2:895514-896353	protein_coding	0.52007102	0.008868218
SMU.946	NC_004350.2:896470-897249	protein_coding	0.404261595	0.080875894
SMU.947	NC_004350.2:897334-897846	protein_coding	1.290262538	2.89879E-11
SMU.949	NC_004350.2:898059-899291	protein_coding	0.825669775	5.33577E-09
SMU.950	NC_004350.2:899307-899900	protein_coding	0.518329884	0.006110927
SMU.951	NC_004350.2:900229-901605	protein_coding	1.619850363	1.84344E-10
SMU.952	NC_004350.2:901615-902565	protein_coding	0.342221093	0.14388468
SMU.953c	NC_004350.2:902699-903961	protein_coding	2.637811843	1.96466E-57
SMU.954	NC_004350.2:904059-904910	protein_coding	-1.887712112	5.23243E-18
SMU.955	NC_004350.2:904907-905440	protein_coding	-1.728139567	3.63327E-08
SMU.956	NC_004350.2:905494-907599	protein_coding	2.386651162	5.72E-124
SMU.957	NC_004350.2:907994-908497	protein_coding	-0.318919993	0.034447724
SMU.960	NC_004350.2:908563-908931	protein_coding	-0.15194081	0.364880899
SMU.961	NC_004350.2:909198-909749	protein_coding	1.590167979	6.6726E-05
SMU.962	NC_004350.2:909770-910837	protein_coding	0.404357989	0.202122562

SMU.963c	NC_004350.2:911429-912328	protein_coding	-1.372756009	1.36348E-39
SMU.965	NC_004350.2:912434-913720	protein_coding	0.706830846	1.19714E-06
SMU.966	NC_004350.2:913722-914588	protein_coding	0.812237089	5.21636E-09
SMU.967	NC_004350.2:914758-916035	protein_coding	0.486579088	0.000178243
SMU.968	NC_004350.2:916056-916619	protein_coding	0.440517174	0.01973838
SMU.969	NC_004350.2:916629-917429	protein_coding	0.207487933	0.176440905
SMU.970	NC_004350.2:917437-917799	protein_coding	-0.064096391	0.764433552
SMU.971	NC_004350.2:917796-918275	protein_coding	-0.134773712	0.433709403
SMU.972	NC_004350.2:918410-919330	protein_coding	-0.374413855	0.008014318
SMU.973	NC_004350.2:919334-920488	protein_coding	-0.567456579	8.27186E-05
SMU.974	NC_004350.2:920472-921266	protein_coding	-0.861864647	2.54647E-09
SMU.975	NC_004350.2:921263-922039	protein_coding	-0.714995905	4.10732E-07
SMU.976	NC_004350.2:922032-923105	protein_coding	-0.658306204	3.3363E-06
SMU.977	NC_004350.2:923185-924027	pseudogene	-0.36055414	0.024961285
SMU.980	NC_004350.2:924428-926362	protein_coding	1.053479904	3.80186E-09
NA	NC_004350.2:926535-928017	pseudogene	1.664000846	1.84213E-08
SMU.983	NC_004350.2:928250-929155	protein_coding	-0.413048739	0.0187832
SMU.984	NC_004350.2:929340-929840	protein_coding	-2.99228967	9.74162E-97
SMU.985	NC_004350.2:929974-931410	protein_coding	-1.082311785	4.34541E-15
SMU.986c	NC_004350.2:931498-932013	protein_coding	-2.448814779	1.08943E-43
SMU.987	NC_004350.2:932362-933723	protein_coding	0.164161709	0.279072151
SMU.988	NC_004350.2:933887-935425	protein_coding	0.431857374	0.001010739
NA	NC_004350.2:935530-935658	protein_coding	1.678942885	0.002050178
SMU.989	NC_004350.2:935710-936786	protein_coding	0.378408528	0.000358858
SMU.990	NC_004350.2:936827-937708	protein_coding	-0.084510752	0.43917625
SMU.991	NC_004350.2:938016-938354	protein_coding	0.254331373	0.237465095
SMU.992	NC_004350.2:938639-939604	protein_coding	1.779290444	9.00509E-21
SMU.993	NC_004350.2:939801-940652	protein_coding	1.306480746	1.5105E-20
SMU.994	NC_004350.2:940639-941421	protein_coding	1.01646022	1.21614E-07
SMU.995	NC_004350.2:941595-942557	protein_coding	0.989034832	4.17403E-11
SMU.996	NC_004350.2:942554-943525	protein_coding	0.889194092	9.52225E-09
SMU.997	NC_004350.2:943522-944328	protein_coding	0.91498659	1.27287E-10
SMU.998	NC_004350.2:944376-945392	protein_coding	0.71067158	4.0344E-08
SMU.999	NC_004350.2:945464-945745	protein_coding	-0.927296852	0.007305708
SMU.1001	NC_004350.2:945912-946754	protein_coding	-5.195105396	6.1232E-157
SMU.1002	NC_004350.2:946849-948966	protein_coding	-3.393012136	2.6222E-135
SMU.1003	NC_004350.2:949332-950666	protein_coding	-3.075586379	7.6303E-114
SMU.1004	NC_004350.2:951112-955542	protein_coding	0.852745269	1.63329E-11
SMU.1005	NC_004350.2:955738-960105	protein_coding	0.429337824	0.004888913
SMU.1006	NC_004350.2:960370-961122	protein_coding	0.508184663	0.212213069
SMU.1007	NC_004350.2:961124-963127	protein_coding	0.265377141	0.374221726
SMU.1008	NC_004350.2:963169-963840	protein_coding	-0.317154859	0.244082286
SMU.1009	NC_004350.2:963837-964790	protein_coding	-0.108670596	0.637917916
SMU.1010	NC_004350.2:964941-965990	protein_coding	-1.732361365	2.08255E-31
SMU.1011	NC_004350.2:966166-967056	protein_coding	-1.877300829	1.37626E-28
SMU.1012c	NC_004350.2:967062-967751	protein_coding	-1.854104984	5.10708E-22
SMU.1013c	NC_004350.2:967981-969384	protein_coding	-1.922590883	3.5035E-49
SMU.1014	NC_004350.2:969678-970007	protein_coding	-1.685833136	1.04981E-24
SMU.1016	NC_004350.2:970026-970418	protein_coding	-2.166425125	6.19462E-28
SMU.1017	NC_004350.2:970441-971562	protein_coding	-2.180662895	2.01956E-76
SMU.1019	NC_004350.2:971809-972117	protein_coding	-2.306533508	6.00996E-45
SMU.1020	NC_004350.2:972105-973007	protein_coding	-1.883532845	1.27889E-59
SMU.1021	NC_004350.2:972994-974529	protein_coding	-2.243534957	5.12078E-96
SMU.1022	NC_004350.2:974522-975058	protein_coding	-2.121384447	1.09581E-53
SMU.1023	NC_004350.2:975071-976462	protein_coding	-2.270449242	4.67007E-76
SMU.1024c	NC_004350.2:976530-976667	pseudogene	3.190284595	0.186040485
SMU.1025	NC_004350.2:976838-977263	protein_coding	0.955207478	0.037584987
SMU.1026	NC_004350.2:977310-977690	protein_coding	1.29150423	0.001955959
SMU.1027	NC_004350.2:977808-978446	protein_coding	2.148216594	2.0066E-32

SMU.1028	NC_004350.2:978482-979516	protein_coding	2.472567708	4.8787E-81
NA	NC_004350.2:979522-979710	protein_coding	1.870026565	3.84249E-12
SMU.1029	NC_004350.2:980040-980479	pseudogene	3.583625466	0.107136793
SMU.1030	NC_004350.2:980476-980706	protein_coding	2.372502883	0.378828854
SMU.1031	NC_004350.2:981073-981276	protein_coding	1.950456055	0.615382071
SMU.1032	NC_004350.2:981357-982572	pseudogene	1.683184455	0.000178673
SMU.1034c	NC_004350.2:982973-984043	protein_coding	0.538916493	2.6292E-05
SMU.1035	NC_004350.2:984162-985043	protein_coding	2.062325787	1.72243E-06
SMU.1036	NC_004350.2:985064-985807	protein_coding	2.395157536	1.23355E-08
SMU.1037c	NC_004350.2:985855-986733	protein_coding	1.77429056	2.26887E-46
SMU.1038c	NC_004350.2:986733-987425	protein_coding	-0.933884999	1.49713E-14
SMU.1039c	NC_004350.2:987558-988391	protein_coding	-1.069090922	5.01678E-18
SMU.1040c	NC_004350.2:988407-989162	protein_coding	-1.088992108	9.94891E-13
SMU.1041	NC_004350.2:989323-990069	protein_coding	-2.018128688	6.37485E-31
SMU.1042	NC_004350.2:990066-991673	protein_coding	-2.743459398	5.37086E-84
SMU.1043c	NC_004350.2:991774-992769	protein_coding	-1.318364872	1.76665E-29
SMU.1044c	NC_004350.2:992786-993676	protein_coding	0.35424655	0.009894929
SMU.1045c	NC_004350.2:993673-994506	protein_coding	0.37757474	0.017301445
SMU.1046c	NC_004350.2:994481-995146	protein_coding	1.028023741	1.37781E-11
NA	NC_004350.2:995233-995412	protein_coding	0.453706553	0.137649656
SMU.1048	NC_004350.2:995645-996211	protein_coding	0.296543584	0.291422147
SMU.1050	NC_004350.2:996314-997294	protein_coding	-1.37416265	1.16017E-30
SMU.1051	NC_004350.2:997298-998416	protein_coding	-1.192388487	6.64386E-30
SMU.1052	NC_004350.2:998418-998765	protein_coding	-1.599352027	3.08075E-45
SMU.1053	NC_004350.2:999061-999702	protein_coding	-0.498903013	0.030417868
SMU.1054	NC_004350.2:999726-1000421	protein_coding	-0.255669583	0.307786804
SMU.1055	NC_004350.2:1000418-1001098	protein_coding	-3.208941489	1.3558E-116
SMU.1057	NC_004350.2:1001196-1001933	protein_coding	-0.329633299	0.109412096
SMU.1058	NC_004350.2:1001930-1002598	protein_coding	-0.670247884	6.30823E-08
SMU.1059	NC_004350.2:1002695-1003222	protein_coding	0.03088638	0.857423798
SMU.1060	NC_004350.2:1003242-1004792	protein_coding	0.138811046	0.20178875
SMU.1061	NC_004350.2:1004905-1005237	protein_coding	0.73368894	4.86791E-06
SMU.1062	NC_004350.2:1005347-1007077	protein_coding	3.485188423	3.303E-212
SMU.1063	NC_004350.2:1007080-1008294	protein_coding	4.146996426	1.8961E-179
SMU.1064c	NC_004350.2:1008567-1009199	protein_coding	-0.276873203	0.410180174
SMU.1065c	NC_004350.2:1009203-1009901	protein_coding	-0.849381595	0.000113464
SMU.1066	NC_004350.2:1010105-1011658	protein_coding	0.365514465	0.016988599
SMU.1067c	NC_004350.2:1011784-1012572	protein_coding	-2.156634439	9.4152E-104
SMU.1068c	NC_004350.2:1012553-1013413	protein_coding	-1.951352101	1.46632E-75
SMU.1069c	NC_004350.2:1013423-1013869	protein_coding	-2.01885808	1.27806E-46
SMU.1070c	NC_004350.2:1013859-1014386	protein_coding	-2.315839586	2.74899E-73
SMU.1071c	NC_004350.2:1014550-1015401	protein_coding	1.799557944	2.5562E-15
SMU.1072c	NC_004350.2:1015450-1015941	protein_coding	5.377618282	1.83346E-06
SMU.1073	NC_004350.2:1016005-1017675	protein_coding	1.377461424	8.68767E-41
SMU.1074	NC_004350.2:1017842-1018528	protein_coding	-1.628244447	4.57343E-17
SMU.1075	NC_004350.2:1018521-1019060	protein_coding	-1.267565702	5.7938E-12
SMU.1076	NC_004350.2:1019135-1019707	protein_coding	-0.649471647	0.000248552
SMU.1077	NC_004350.2:1019802-1021517	protein_coding	-1.850174003	7.32444E-76
SMU.1078c	NC_004350.2:1021648-1023393	protein_coding	-0.784207591	6.28624E-09
SMU.1079c	NC_004350.2:1023390-1025123	protein_coding	-0.648340477	1.6338E-06
SMU.1080c	NC_004350.2:1025133-1025747	protein_coding	-0.216625275	0.149979833
SMU.1081c	NC_004350.2:1025749-1026726	protein_coding	-0.538141118	2.37693E-05
SMU.1082	NC_004350.2:1026723-1027985	protein_coding	0.11510334	0.433709403
SMU.1083c	NC_004350.2:1027995-1028585	protein_coding	0.527996501	0.00434026
SMU.1084	NC_004350.2:1028578-1029414	protein_coding	0.080726069	0.601265684
SMU.1085	NC_004350.2:1029411-1030490	protein_coding	0.872652524	1.03018E-07
SMU.1086	NC_004350.2:1030550-1031125	protein_coding	-0.351399491	0.040701418
SMU.1087	NC_004350.2:1031284-1031469	protein_coding	-1.151833119	0.000242761
SMU.1088	NC_004350.2:1031551-1032486	protein_coding	-2.555525257	3.97533E-97

SMU.1089	NC_004350.2:1032511-1033113	protein_coding	-2.108182499	2.44033E-82
SMU.1090	NC_004350.2:1033133-1034392	protein_coding	-2.347141874	1.956E-133
SMU.1091	NC_004350.2:1034634-1036157	protein_coding	0.973365253	7.73887E-14
SMU.1093	NC_004350.2:1036487-1037995	protein_coding	0.265698499	0.521712043
SMU.1094	NC_004350.2:1038011-1038667	protein_coding	0.45435507	0.432019831
SMU.1095	NC_004350.2:1038786-1040306	protein_coding	-0.777030103	1.87903E-07
SMU.1096	NC_004350.2:1040306-1041028	protein_coding	-0.43065419	0.01120457
SMU.1097c	NC_004350.2:1041025-1041507	protein_coding	-0.210995545	0.33255999
SMU.1098c	NC_004350.2:1041507-1042469	protein_coding	0.195337421	0.324805573
SMU.1100c	NC_004350.2:1042742-1043866	protein_coding	1.219497628	4.76965E-13
SMU.1102	NC_004350.2:1044251-1045687	protein_coding	-0.00870673	0.959619536
SMU.1104c	NC_004350.2:1045700-1046293	pseudogene	0.302507805	0.255503586
SMU.1105c	NC_004350.2:1046290-1046904	protein_coding	0.011944943	0.963547068
SMU.1106c	NC_004350.2:1046916-1047512	protein_coding	-0.105397675	0.677719422
SMU.1107c	NC_004350.2:1048069-1048710	protein_coding	0.180481802	0.352088657
SMU.1108c	NC_004350.2:1048776-1049588	protein_coding	0.323121889	0.075408456
SMU.1109c	NC_004350.2:1049803-1051056	protein_coding	0.736646498	3.38476E-08
SMU.1111c	NC_004350.2:1051336-1052307	protein_coding	0.458383298	0.002538473
SMU.1112c	NC_004350.2:1052392-1052796	protein_coding	0.872136569	3.41135E-07
SMU.1113	NC_004350.2:1053014-1053754	protein_coding	0.435398738	0.000985267
SMU.1114	NC_004350.2:1053761-1056223	protein_coding	0.445609281	4.26378E-06
SMU.1115	NC_004350.2:1056409-1057395	protein_coding	-0.657248354	8.51537E-14
SMU.1116c	NC_004350.2:1057457-1058593	protein_coding	0.745203981	1.97271E-15
SMU.1117	NC_004350.2:1058760-1060133	protein_coding	2.010346785	2.37291E-63
SMU.1118c	NC_004350.2:1060373-1061329	protein_coding	-1.340591756	1.22428E-26
SMU.1119c	NC_004350.2:1061331-1062407	protein_coding	-0.970832849	2.77186E-08
SMU.1120	NC_004350.2:1062400-1063932	protein_coding	-1.00385258	4.92059E-19
SMU.1121c	NC_004350.2:1064050-1065099	protein_coding	-0.978282763	1.81581E-19
SMU.1122	NC_004350.2:1065189-1065575	protein_coding	-0.91534089	2.44285E-11
SMU.1123	NC_004350.2:1065565-1066227	protein_coding	-1.292947961	2.22123E-21
SMU.1124	NC_004350.2:1066246-1067523	protein_coding	-2.193237959	8.09109E-44
SMU.1125c	NC_004350.2:1067632-1068228	protein_coding	-2.313151978	6.32581E-30
SMU.1126	NC_004350.2:1068324-1069244	protein_coding	-2.205023376	3.12473E-42
SMU.1127	NC_004350.2:1069298-1069552	protein_coding	-1.374548863	0.001780857
SMU.1128	NC_004350.2:1069642-1070949	protein_coding	-1.006421237	1.7948E-14
SMU.1129	NC_004350.2:1070939-1071613	protein_coding	-0.615850145	0.005423761
SMU.1131c	NC_004350.2:1071664-1071927	protein_coding	0.366789786	0.533793986
SMU.1132	NC_004350.2:1072328-1074877	protein_coding	-0.289710029	0.004633157
SMU.1133	NC_004350.2:1074918-1075571	protein_coding	-0.978326604	1.24786E-07
SMU.1134c	NC_004350.2:1075603-1076361	protein_coding	-0.615843424	4.92709E-05
SMU.1135	NC_004350.2:1076373-1077176	protein_coding	-0.724017847	2.12852E-06
SMU.1136	NC_004350.2:1077192-1078079	protein_coding	-0.302049684	0.032652128
SMU.1137	NC_004350.2:1078069-1079004	protein_coding	-0.014715745	0.933614457
SMU.1138	NC_004350.2:1079077-1079940	protein_coding	0.081663829	0.69596838
SMU.1139c	NC_004350.2:1080037-1081347	protein_coding	0.189928451	0.26724421
SMU.1140c	NC_004350.2:1081411-1082169	protein_coding	0.857971838	0.000230377
SMU.1141c	NC_004350.2:1082159-1082437	protein_coding	1.251718312	0.11072408
SMU.1142c	NC_004350.2:1082430-1082843	protein_coding	-1.096441957	4.93263E-14
SMU.1143c	NC_004350.2:1082904-1083824	protein_coding	0.566069815	0.0013767
SMU.1144	NC_004350.2:1083855-1084736	protein_coding	-0.214679136	0.218713027
SMU.1145c	NC_004350.2:1084846-1086159	protein_coding	-1.091410397	3.01533E-08
SMU.1146c	NC_004350.2:1086160-1086849	protein_coding	-1.01756943	0.000752331
SMU.1148	NC_004350.2:1087024-1087932	protein_coding	3.276829171	1.12213E-10
SMU.1149	NC_004350.2:1087932-1088672	protein_coding	3.853472259	2.15964E-10
SMU.1150	NC_004350.2:1088669-1089397	protein_coding	3.520771718	2.27059E-10
SMU.1151c	NC_004350.2:1089516-1090880	protein_coding	-1.661443235	2.80131E-50
SMU.1152c	NC_004350.2:1090880-1091113	protein_coding	-2.319878162	6.48673E-16
SMU.1153c	NC_004350.2:1091286-1092197	protein_coding	-2.183189157	9.66886E-49
NA	NC_004350.2:1092296-1092487	pseudogene	-2.07882955	5.88107E-22

SMU.1154c	NC_004350.2:1092616-1092912	protein_coding	-1.730050428	4.49302E-08
SMU.1156c	NC_004350.2:1092916-1093350	protein_coding	-1.939393502	6.19968E-14
SMU.1157c	NC_004350.2:1093482-1096667	protein_coding	-1.999813546	3.08655E-42
SMU.1158c	NC_004350.2:1096679-1097935	protein_coding	-1.802546951	4.10971E-28
SMU.1160c	NC_004350.2:1098230-1099252	protein_coding	-2.321381093	6.60696E-47
SMU.1161c	NC_004350.2:1099325-1100071	protein_coding	-1.951813796	1.3482E-16
SMU.1163c	NC_004350.2:1100349-1102094	protein_coding	-0.031438517	0.872394781
SMU.1164c	NC_004350.2:1102087-1103859	protein_coding	0.071811791	0.760233023
SMU.1165c	NC_004350.2:1103870-1104481	protein_coding	-0.261166846	0.667079609
SMU.1166c	NC_004350.2:1104674-1107307	protein_coding	0.874565591	1.76397E-06
SMU.1167c	NC_004350.2:1107323-1108024	protein_coding	1.231310716	0.00869787
SMU.1168	NC_004350.2:1108137-1108685	protein_coding	1.155164682	0.000494474
SMU.1169c	NC_004350.2:1108809-1109372	protein_coding	0.115653369	0.600519188
SMU.1170	NC_004350.2:1109386-1110099	protein_coding	0.213934743	0.362423864
SMU.1171c	NC_004350.2:1110296-1111066	protein_coding	-0.645021012	0.003827511
SMU.1172c	NC_004350.2:1111163-1111903	protein_coding	-1.716184494	8.45985E-61
SMU.1173	NC_004350.2:1111917-1113197	protein_coding	-1.512802711	4.25432E-55
SMU.1174	NC_004350.2:1113511-1115787	protein_coding	-1.514665556	1.77337E-57
SMU.1175	NC_004350.2:1116158-1117522	protein_coding	2.817912703	2.71589E-20
SMU.1176	NC_004350.2:1117621-1118811	protein_coding	1.327657708	1.02707E-10
SMU.1177c	NC_004350.2:1118934-1119767	protein_coding	-0.529032526	0.000227292
SMU.1178c	NC_004350.2:1119789-1120418	protein_coding	-0.49248769	0.004211953
SMU.1179c	NC_004350.2:1120427-1121068	protein_coding	-0.361127789	0.021460435
SMU.1180	NC_004350.2:1121259-1121591	protein_coding	-0.856796482	2.26157E-06
SMU.1182	NC_004350.2:1121766-1122914	protein_coding	-0.625808457	3.29336E-05
SMU.1183	NC_004350.2:1122930-1123367	protein_coding	-0.539461507	0.036448463
SMU.1184c	NC_004350.2:1123369-1125321	protein_coding	-0.688471506	4.87232E-08
SMU.1185	NC_004350.2:1125351-1127120	protein_coding	-0.016256029	0.938292359
SMU.1187	NC_004350.2:1127608-1129422	protein_coding	0.978642385	1.34042E-15
SMU.1188	NC_004350.2:1129595-1130149	protein_coding	-0.246537938	0.194803323
SMU.1189c	NC_004350.2:1130228-1130470	protein_coding	0.666615367	0.034948515
SMU.1190	NC_004350.2:1130761-1132263	protein_coding	-1.023978253	4.36064E-26
SMU.1191	NC_004350.2:1132337-1133350	protein_coding	-0.920294986	1.45808E-14
SMU.1192	NC_004350.2:1133433-1136537	protein_coding	-1.016581247	4.18994E-32
SMU.1193	NC_004350.2:1136715-1137086	protein_coding	-1.216595228	4.10544E-12
SMU.1194	NC_004350.2:1137083-1137790	protein_coding	-0.85213016	1.18473E-08
SMU.1195	NC_004350.2:1137800-1138618	protein_coding	-0.452385207	0.002465317
SMU.1196c	NC_004350.2:1138671-1139291	protein_coding	0.169863527	0.439012324
NA	NC_004350.2:1139336-1139684	tmRNA	-4.791282257	0
SMU.1197	NC_004350.2:1139764-1140378	protein_coding	0.210860428	0.246919726
SMU_t37	NC_004350.2:1140675-1140746	tRNA	6.518131524	5.57096E-07
SMU.1200	NC_004350.2:1140884-1142083	protein_coding	1.573901535	2.8337E-41
SMU_t38	NC_004350.2:1142216-1142287	tRNA	2.644092835	8.0271E-07
SMU_t39	NC_004350.2:1142444-1142524	tRNA	4.092644755	0.007604078
SMU.1201c	NC_004350.2:1142584-1142814	protein_coding	-0.564991307	0.087915709
SMU.1203	NC_004350.2:1142879-1143934	protein_coding	-1.020250718	5.13643E-15
SMU.1204	NC_004350.2:1144147-1146603	protein_coding	-0.697267425	1.33051E-10
SMU.1205c	NC_004350.2:1146796-1147362	protein_coding	-0.357105838	0.039283756
SMU.1206c	NC_004350.2:1147387-1147944	protein_coding	-0.580256745	0.000250258
SMU.1207	NC_004350.2:1148062-1148667	protein_coding	-0.873387245	2.38366E-07
SMU.1208c	NC_004350.2:1148712-1149461	protein_coding	-0.492676925	0.001001112
SMU.1209c	NC_004350.2:1149743-1151083	protein_coding	-0.531471216	2.12544E-05
SMU.1210	NC_004350.2:1151099-1153048	protein_coding	-0.623909054	3.0364E-06
SMU.1211	NC_004350.2:1153161-1153799	protein_coding	0.187032257	0.346483774
SMU.1213c	NC_004350.2:1153986-1156097	protein_coding	-0.655030583	2.80593E-13
SMU.1214	NC_004350.2:1156286-1157554	protein_coding	-0.618803284	1.06843E-06
SMU.1215	NC_004350.2:1157564-1158217	protein_coding	-0.210043807	0.173222382
SMU.1216c	NC_004350.2:1158240-1158893	protein_coding	0.07880283	0.727444384
SMU.1217c	NC_004350.2:1159043-1159894	protein_coding	-0.278362133	0.079440726

SMU.1218	NC_004350.2:1160009-1161451	protein_coding	-0.143989542	0.350090521
SMU.1219c	NC_004350.2:1161511-1161852	protein_coding	0.031737628	0.908635706
SMU.1220c	NC_004350.2:1162026-1162526	protein_coding	-0.590251841	3.62863E-06
SMU.1221	NC_004350.2:1162858-1163487	protein_coding	-0.489613941	0.000326037
SMU.1222	NC_004350.2:1163588-1164280	protein_coding	0.132876274	0.468496459
SMU.1223	NC_004350.2:1164273-1165202	protein_coding	1.085854877	1.18023E-06
SMU.1224	NC_004350.2:1165212-1165988	protein_coding	1.162372552	2.87467E-07
SMU.1225	NC_004350.2:1166241-1167149	protein_coding	0.199429169	0.230047062
SMU.1226c	NC_004350.2:1167215-1167982	protein_coding	-0.693975822	1.14524E-07
SMU.1227	NC_004350.2:1167984-1168688	protein_coding	-0.309634329	0.009103735
SMU.1228c	NC_004350.2:1168902-1169612	protein_coding	-0.462927976	0.000126709
SMU.1229	NC_004350.2:1169622-1170431	protein_coding	-0.138160328	0.350514548
SMU.1230c	NC_004350.2:1170442-1170918	protein_coding	-0.097184291	0.443823419
SMU.1232c	NC_004350.2:1171241-1171456	protein_coding	-0.331009973	0.069085013
SMU.1233	NC_004350.2:1171458-1172669	protein_coding	0.02747235	0.81130033
SMU.1234	NC_004350.2:1172724-1173401	protein_coding	0.165357505	0.28272399
SMU.1235	NC_004350.2:1173530-1174897	protein_coding	-0.476426192	0.000216616
SMU.1236c	NC_004350.2:1175045-1175848	protein_coding	2.209793541	4.07523E-11
SMU.1237c	NC_004350.2:1175866-1176216	protein_coding	2.750038801	3.79158E-07
NA	NC_004350.2:1176307-1176609	protein_coding	-2.625610322	2.05246E-38
SMU.1238c	NC_004350.2:1176737-1177324	protein_coding	-1.476810015	1.20112E-43
SMU.1239	NC_004350.2:1177397-1178809	protein_coding	-1.082848167	9.96068E-29
SMU.1240c	NC_004350.2:1178824-1179426	protein_coding	-0.84796334	1.85477E-11
SMU.1241	NC_004350.2:1179573-1181360	protein_coding	-0.290919843	0.014027945
SMU.1243	NC_004350.2:1181478-1182621	pseudogene	0.240140771	0.292235049
SMU.1245c	NC_004350.2:1182665-1183360	protein_coding	0.169399371	0.338478616
SMU.1246c	NC_004350.2:1183574-1183870	protein_coding	0.905573463	5.43756E-06
SMU.1247	NC_004350.2:1183934-1185232	protein_coding	-0.659464582	2.82339E-13
SMU.1249c	NC_004350.2:1185629-1186270	protein_coding	0.542249645	0.005224543
SMU.1250c	NC_004350.2:1186308-1186577	protein_coding	0.746909218	0.01522281
SMU.1251	NC_004350.2:1186726-1187178	protein_coding	-1.129155629	1.38143E-05
SMU.1252	NC_004350.2:1187255-1188367	protein_coding	-0.467375077	0.002471248
SMU.1253c	NC_004350.2:1188378-1188803	protein_coding	-1.052255816	2.39295E-05
SMU.1254	NC_004350.2:1188892-1189536	protein_coding	-0.89394057	1.14989E-05
SMU.1255c	NC_004350.2:1189531-1189830	protein_coding	-0.746290129	1.37056E-06
SMU.1257c	NC_004350.2:1190156-1190827	protein_coding	0.055787386	0.679818246
SMU.1258c	NC_004350.2:1190950-1191612	protein_coding	0.421868303	0.297233061
SMU.1259	NC_004350.2:1191675-1192001	protein_coding	2.538103067	0.009598545
SMU.1260c	NC_004350.2:1192355-1192534	protein_coding	-1.184849778	0.000744173
NA	NC_004350.2:1192547-1192861	protein_coding	-1.163394913	1.78662E-06
SMU.1262c	NC_004350.2:1192812-1194152	protein_coding	-0.716548631	0.001559753
SMU.1263	NC_004350.2:1194266-1194595	protein_coding	-0.300330645	0.315795548
SMU.1264	NC_004350.2:1194595-1195350	protein_coding	-0.126149541	0.600519188
SMU.1265	NC_004350.2:1195353-1196072	protein_coding	-0.437998333	0.018346707
SMU.1266	NC_004350.2:1196512-1197117	protein_coding	-0.500979147	0.019101965
SMU.1267c	NC_004350.2:1197114-1197467	protein_coding	0.134094111	0.667079609
SMU.1268	NC_004350.2:1197483-1198067	protein_coding	-0.43775247	0.052823834
SMU.1269	NC_004350.2:1198064-1198711	protein_coding	-0.225338914	0.273316855
SMU.1270	NC_004350.2:1198698-1199981	protein_coding	0.037945249	0.830857405
SMU.1271	NC_004350.2:1199978-1200625	protein_coding	0.340933885	0.341304458
SMU.1272	NC_004350.2:1200635-1201597	protein_coding	0.473788897	0.036288414
SMU.1273	NC_004350.2:1201594-1202643	protein_coding	1.074254631	4.0063E-06
SMU.1276c	NC_004350.2:1203817-1205541	protein_coding	0.197065238	0.039813916
SMU.1277	NC_004350.2:1205637-1207589	protein_coding	0.543931517	9.67853E-09
SMU.1278c	NC_004350.2:1207589-1208173	protein_coding	0.678609112	2.45646E-05
SMU.1279c	NC_004350.2:1208595-1209821	protein_coding	0.384366951	0.007679818
SMU.1280c	NC_004350.2:1210028-1211146	protein_coding	2.257812881	5.64927E-06
SMU.1282	NC_004350.2:1211285-1211851	protein_coding	-0.146370032	0.530014312
SMU_t40	NC_004350.2:1211962-1212033	tRNA	2.394516904	0.141593559

SMU.1284c	NC_004350.2:1212113-1212772	protein_coding	3.389405008	4.05819E-37
SMU.1286c	NC_004350.2:1212774-1213952	protein_coding	3.092063707	4.95307E-36
SMU.1287	NC_004350.2:1214103-1214453	protein_coding	-1.736545089	1.42999E-08
SMU.1288	NC_004350.2:1214632-1214979	protein_coding	1.257801346	2.28395E-26
SMU.1289c	NC_004350.2:1215168-1216388	protein_coding	-0.188165514	0.185187875
SMU.1290c	NC_004350.2:1216474-1217694	protein_coding	-0.670722088	1.21626E-06
SMU.1291c	NC_004350.2:1217684-1217956	protein_coding	-1.295906396	0.000645303
SMU.1292c	NC_004350.2:1218002-1218679	protein_coding	-1.492743505	3.68352E-33
SMU.1293c	NC_004350.2:1219101-1220483	protein_coding	-2.269682602	1.6096E-104
SMU.1294	NC_004350.2:1220643-1221086	protein_coding	0.372946722	0.374145822
SMU.1295	NC_004350.2:1221145-1222194	protein_coding	1.486247083	1.28885E-07
SMU.1296	NC_004350.2:1222269-1223060	protein_coding	1.080521089	2.17264E-10
SMU.1297	NC_004350.2:1223132-1224064	protein_coding	3.33344958	1.31196E-79
SMU.1298	NC_004350.2:1224137-1224403	protein_coding	1.506908431	1.82963E-25
SMU.1299c	NC_004350.2:1224443-1224766	protein_coding	-1.294872715	1.40605E-06
SMU.1300c	NC_004350.2:1224759-1225106	protein_coding	-0.674383273	0.05120079
SMU.1301c	NC_004350.2:1225160-1225915	protein_coding	-0.508995432	0.028627668
SMU.1302	NC_004350.2:1226076-1227596	protein_coding	-0.581663341	9.18565E-05
SMU.1303c	NC_004350.2:1227823-1229220	protein_coding	-1.306197614	5.13244E-41
SMU.1304c	NC_004350.2:1229586-1230497	protein_coding	-1.669107655	1.47298E-36
SMU.1305c	NC_004350.2:1230494-1231471	protein_coding	-1.4187811	2.96107E-26
SMU.1306c	NC_004350.2:1231468-1232358	protein_coding	-1.357474635	4.3414E-26
SMU.1307c	NC_004350.2:1232351-1233109	protein_coding	-1.412012839	1.27985E-28
SMU.1308	NC_004350.2:1233240-1233617	protein_coding	-1.254427787	9.89321E-14
SMU.1309c	NC_004350.2:1233647-1234738	protein_coding	-0.976705537	1.25462E-10
SMU.1311	NC_004350.2:1235250-1236596	protein_coding	-0.421500214	0.000130688
SMU.1312	NC_004350.2:1236643-1237824	protein_coding	-0.148164682	0.292930339
SMU.1313c	NC_004350.2:1237890-1240418	protein_coding	0.311710997	0.015012926
SMU.1314	NC_004350.2:1240611-1241444	protein_coding	0.550096829	0.000905797
SMU.1315c	NC_004350.2:1241656-1242309	protein_coding	0.900597167	6.33372E-06
SMU.1316c	NC_004350.2:1242311-1243042	protein_coding	0.290837367	0.403505621
SMU.1317c	NC_004350.2:1243080-1243382	protein_coding	-0.113263614	0.801946148
NA	NC_004350.2:1243388-1243573	protein_coding	-0.671163701	0.114151655
SMU.1319c	NC_004350.2:1244138-1244890	protein_coding	0.068080842	0.722522017
SMU.1321c	NC_004350.2:1245032-1246210	protein_coding	-0.126878094	0.470528673
SMU.1322	NC_004350.2:1246354-1247118	protein_coding	-1.662898236	5.97941E-35
SMU.1323	NC_004350.2:1247243-1247881	protein_coding	0.075379037	0.73483265
SMU.1324	NC_004350.2:1248004-1248939	protein_coding	0.675457718	1.48379E-07
SMU.1325	NC_004350.2:1248932-1249624	protein_coding	1.084241449	6.04293E-17
SMU.1326	NC_004350.2:1249646-1250741	protein_coding	1.299071836	1.50294E-24
SMU.1327c	NC_004350.2:1250791-1251915	protein_coding	-0.34860421	0.16970744
SMU.1329c	NC_004350.2:1252575-1253157	pseudogene	0.908779935	0.007394752
NA	NC_004350.2:1253191-1254436	pseudogene	1.07637832	0.004309139
NA	NC_004350.2:1254558-1254848	pseudogene	-2.863398613	2.0757E-109
SMU.1334	NC_004350.2:1255084-1255776	protein_coding	-3.557629272	5.6942E-200
SMU.1335c	NC_004350.2:1255804-1256787	protein_coding	-3.050624227	3.7888E-117
SMU.1336	NC_004350.2:1256800-1257738	protein_coding	-3.412781879	1.558E-153
SMU.1337c	NC_004350.2:1257767-1258516	protein_coding	-3.132189722	4.29741E-82
SMU.1338c	NC_004350.2:1258545-1259762	protein_coding	-3.066958728	1.55998E-84
SMU.1339	NC_004350.2:1259790-1264157	protein_coding	-3.0587467	4.8385E-117
SMU.1340	NC_004350.2:1264255-1269141	protein_coding	-3.11117308	4.5433E-135
SMU.1341c	NC_004350.2:1269141-1272830	protein_coding	-2.50986339	1.61842E-88
SMU.1342	NC_004350.2:1272849-1281023	protein_coding	-2.312698342	2.41349E-59
SMU.1343c	NC_004350.2:1281069-1284422	protein_coding	-2.259674224	1.11547E-49
SMU.1344c	NC_004350.2:1284428-1285645	protein_coding	-2.151338882	1.23319E-22
SMU.1345c	NC_004350.2:1285660-1287561	protein_coding	-1.502195516	8.84641E-15
SMU.1346	NC_004350.2:1287573-1288292	protein_coding	-1.570125184	8.4947E-15
SMU.1347c	NC_004350.2:1288285-1290627	protein_coding	-4.168869761	0.034817646
SMU.1348c	NC_004350.2:1290632-1291333	protein_coding	-1.835023839	0.126315218

SMU.1349	NC_004350.2:1291686-1292261	protein_coding	-0.470935422	0.000793011
NA	NC_004350.2:1292447-1293377	pseudogene	-0.046582603	0.842601979
NA	NC_004350.2:1293395-1294552	pseudogene	-2.357487368	3.54985E-53
SMU.1357	NC_004350.2:1294643-1294970	pseudogene	3.441854458	0.036767785
SMU.1361c	NC_004350.2:1295378-1295959	protein_coding	-2.63798597	2.767E-208
NA	NC_004350.2:1296600-1297064	pseudogene	-3.025608076	2.9082E-215
SMU.1365c	NC_004350.2:1297315-1299657	protein_coding	-3.176190913	3.185E-100
SMU.1366c	NC_004350.2:1299662-1300363	protein_coding	NA	NA
SMU.1367c	NC_004350.2:1300376-1300975	protein_coding	-2.259777998	9.46843E-53
SMU.1370c	NC_004350.2:1302181-1302972	protein_coding	1.59445482	0.142321036
NA	NC_004350.2:1303045-1303365	protein_coding	1.224375417	0.601265684
SMU.1375c	NC_004350.2:1305078-1305683	protein_coding	0.653006896	0.000652961
SMU.1377c	NC_004350.2:1305801-1306586	protein_coding	-0.233038438	0.11278793
SMU.1378	NC_004350.2:1306651-1307187	protein_coding	0.118361315	0.813253329
NA	NC_004350.2:1307343-1308691	protein_coding	-1.27369269	0.39049225
SMU.1381	NC_004350.2:1308800-1309390	protein_coding	-0.673427251	1.15132E-07
SMU.1382	NC_004350.2:1309392-1310777	protein_coding	-0.551326623	4.13683E-07
SMU.1383	NC_004350.2:1310903-1311937	protein_coding	-1.037764118	1.12229E-16
SMU.1384	NC_004350.2:1311947-1313512	protein_coding	-0.526973544	2.27661E-06
SMU.1386	NC_004350.2:1313838-1314467	protein_coding	-0.575626676	0.001054342
SMU.1387	NC_004350.2:1314686-1315666	protein_coding	-0.459546879	0.001631398
SMU.1388	NC_004350.2:1315663-1316745	protein_coding	-0.533389704	0.000202466
SMU.1389	NC_004350.2:1316938-1318578	protein_coding	-1.563207418	5.38675E-49
SMU.1390	NC_004350.2:1318897-1319553	protein_coding	-0.023691597	0.949495232
SMU.1391c	NC_004350.2:1319618-1320226	protein_coding	-0.540401519	8.66148E-05
SMU.1392c	NC_004350.2:1320306-1320782	protein_coding	0.963449013	1.52684E-11
SMU.1393c	NC_004350.2:1320861-1321688	protein_coding	0.679301959	8.95184E-07
SMU.1394	NC_004350.2:1321717-1323552	protein_coding	1.267334658	4.16527E-29
SMU.1396	NC_004350.2:1324324-1326075	protein_coding	5.144248994	0
SMU.1397c	NC_004350.2:1326238-1326523	pseudogene	-1.308307037	2.42112E-13
SMU.1398	NC_004350.2:1326838-1327731	protein_coding	-2.992044173	2.3432E-113
SMU.1400c	NC_004350.2:1328400-1328777	protein_coding	-0.593781583	0.005754718
NA	NC_004350.2:1328795-1328986	protein_coding	-1.195627559	0.000523265
SMU.1402c	NC_004350.2:1329083-1329745	protein_coding	-4.308632718	1.8857E-198
SMU.1403c	NC_004350.2:1329735-1330079	protein_coding	-3.765664087	1.31601E-38
SMU.1404c	NC_004350.2:1330076-1330942	protein_coding	-4.073075173	7.0767E-188
SMU.1405c	NC_004350.2:1330942-1334979	protein_coding	-3.995543608	0
SMU.1406c	NC_004350.2:1335360-1336226	protein_coding	-1.993575627	2.17638E-20
NA	NC_004350.2:1336342-1337690	protein_coding	-0.466586551	0.922599346
SMU.1409c	NC_004350.2:1337785-1338651	protein_coding	-1.709670589	6.39692E-26
SMU.1410	NC_004350.2:1338776-1340209	protein_coding	1.332771427	4.3324E-06
SMU.1411	NC_004350.2:1340276-1341451	protein_coding	1.134867425	0.000279139
SMU.1412c	NC_004350.2:1341627-1344155	protein_coding	-1.599052199	2.24773E-47
SMU.1414c	NC_004350.2:1344250-1344909	protein_coding	-1.011411677	8.07639E-11
SMU.1415c	NC_004350.2:1344899-1345672	protein_coding	-1.038889847	1.13563E-14
SMU.1416c	NC_004350.2:1345687-1346304	protein_coding	-1.217767584	1.64216E-13
SMU.1417c	NC_004350.2:1346325-1347053	protein_coding	-0.927902194	6.0023E-08
SMU.1418	NC_004350.2:1347056-1348198	protein_coding	-0.595759108	0.000863525
SMU.1419	NC_004350.2:1348733-1349329	protein_coding	3.080313234	7.56237E-63
SMU.1420	NC_004350.2:1349326-1349892	protein_coding	3.166599427	1.36098E-61
SMU.1421	NC_004350.2:1349952-1351205	protein_coding	-1.997900888	2.3558E-81
SMU.1422	NC_004350.2:1351219-1352250	protein_coding	-1.97375916	8.32854E-74
SMU.1423	NC_004350.2:1352263-1353336	protein_coding	-2.152120051	9.01059E-58
SMU.1424	NC_004350.2:1353362-1354699	protein_coding	-2.468521887	2.64386E-92
SMU.1425	NC_004350.2:1354928-1357510	protein_coding	-1.750404035	9.00372E-35
SMU.1426c	NC_004350.2:1357703-1359052	protein_coding	-0.373777063	0.000261165
SMU.1427c	NC_004350.2:1359103-1360059	protein_coding	0.036741152	0.763979825
SMU.1428c	NC_004350.2:1360052-1360906	protein_coding	0.243529228	0.106283861
SMU.1429	NC_004350.2:1361015-1362358	protein_coding	0.642513724	4.40169E-05

SMU.1430	NC_004350.2:1362358-1363146	protein_coding	0.671872064	0.000854568
SMU.1431c	NC_004350.2:1363390-1365303	protein_coding	1.089595065	6.35368E-17
SMU.1432c	NC_004350.2:1365385-1366494	protein_coding	2.009007569	1.15184E-45
SMU.1434c	NC_004350.2:1366506-1367816	protein_coding	2.131641768	3.56766E-45
SMU.1436c	NC_004350.2:1367963-1368751	protein_coding	2.564495261	1.07584E-47
SMU.1437	NC_004350.2:1368759-1369907	protein_coding	2.843415957	2.83571E-63
SMU.1438c	NC_004350.2:1370234-1370932	protein_coding	2.094652966	1.05132E-16
SMU.1442c	NC_004350.2:1371533-1372063	protein_coding	-0.600660994	7.08488E-05
SMU.1443c	NC_004350.2:1372155-1372946	protein_coding	-0.252975464	0.038110075
NA	NC_004350.2:1373026-1373340	protein_coding	0.048381719	0.922599346
SMU.1444c	NC_004350.2:1373434-1375098	protein_coding	1.140438297	6.31316E-21
SMU.1445c	NC_004350.2:1375345-1376103	protein_coding	0.336385923	0.134223648
SMU.1446c	NC_004350.2:1376100-1376969	protein_coding	0.029970981	0.908635706
SMU.1447c	NC_004350.2:1377080-1378081	protein_coding	0.291537947	0.069773602
SMU.1449	NC_004350.2:1378343-1379992	protein_coding	-0.39709361	0.005655463
SMU.1450	NC_004350.2:1380297-1381694	protein_coding	0.128004297	0.365267943
SMU.1451	NC_004350.2:1381765-1382481	protein_coding	-0.113416047	0.464447654
SMU.1452	NC_004350.2:1382495-1384174	protein_coding	0.214215767	0.08974228
SMU.1453c	NC_004350.2:1384307-1385539	protein_coding	-0.872599601	4.62356E-11
SMU.1454c	NC_004350.2:1385529-1386698	protein_coding	-0.661889572	1.8137E-05
SMU.1455	NC_004350.2:1386767-1387246	protein_coding	-0.848989837	0.000167671
SMU.1457	NC_004350.2:1387541-1388587	protein_coding	-0.582594218	2.70609E-07
SMU.1460	NC_004350.2:1389227-1389823	protein_coding	-0.252183378	0.11111428
SMU.1461	NC_004350.2:1389825-1390694	protein_coding	-0.628484317	6.33517E-08
SMU.1462c	NC_004350.2:1390759-1391862	protein_coding	-0.612335635	2.21375E-05
SMU.1463c	NC_004350.2:1391889-1392677	protein_coding	-0.313632373	0.114101564
SMU.1464c	NC_004350.2:1392667-1393365	protein_coding	-0.143571792	0.537153843
SMU.1465c	NC_004350.2:1393355-1394050	protein_coding	0.023062186	0.938292359
SMU.1466	NC_004350.2:1394060-1395004	protein_coding	-0.202344561	0.169529624
SMU.1467	NC_004350.2:1395108-1395626	protein_coding	0.375589734	0.061457914
SMU.1470c	NC_004350.2:1396299-1396931	protein_coding	-0.004070392	0.987447985
SMU.1471c	NC_004350.2:1397184-1398083	protein_coding	-0.091943022	0.722522017
SMU.1472	NC_004350.2:1398203-1400407	protein_coding	-0.081257706	0.501631422
SMU.1473c	NC_004350.2:1400404-1401168	protein_coding	0.238784334	0.087659483
SMU.1474c	NC_004350.2:1401165-1402094	protein_coding	0.985609571	4.39796E-08
SMU.1475c	NC_004350.2:1402122-1402757	protein_coding	1.187304771	2.51572E-17
SMU.1476c	NC_004350.2:1402744-1403991	protein_coding	0.910479728	2.99996E-12
SMU.1477	NC_004350.2:1404180-1405064	protein_coding	1.322994436	1.02482E-05
SMU.1479	NC_004350.2:1405139-1405315	protein_coding	-0.530434858	0.050603852
SMU.1480	NC_004350.2:1405637-1406278	protein_coding	1.481929407	2.22856E-09
SMU.1482c	NC_004350.2:1406343-1406969	protein_coding	-0.256272982	0.147050889
SMU.1483c	NC_004350.2:1406966-1407508	protein_coding	-0.140641481	0.576132096
SMU.1484c	NC_004350.2:1407505-1408824	protein_coding	-0.164603799	0.27889517
SMU.1485c	NC_004350.2:1408811-1410511	protein_coding	0.0090647	0.957945292
SMU.1486c	NC_004350.2:1410521-1411255	protein_coding	1.036543949	2.34761E-05
SMU.1487	NC_004350.2:1411476-1412621	protein_coding	0.128070465	0.600519188
SMU.1488c	NC_004350.2:1412816-1413085	protein_coding	4.489426504	1.39595E-91
SMU.1489	NC_004350.2:1413105-1414001	protein_coding	4.895241728	2.9845E-164
SMU.1490	NC_004350.2:1414248-1415654	protein_coding	4.05065388	1.52181E-48
SMU.1491	NC_004350.2:1415666-1417372	protein_coding	4.075159753	9.2155E-46
SMU.1492	NC_004350.2:1417379-1417693	protein_coding	3.916237913	3.62463E-95
SMU.1493	NC_004350.2:1417709-1418686	protein_coding	3.952911267	4.4196E-131
SMU.1494	NC_004350.2:1418691-1419623	protein_coding	4.116899737	1.34693E-43
SMU.1495	NC_004350.2:1419642-1420157	protein_coding	3.875733217	1.0145E-124
SMU.1496	NC_004350.2:1420183-1420611	protein_coding	3.374526321	7.05725E-52
SMU.1498	NC_004350.2:1420918-1421673	protein_coding	0.701370687	0.00039905
SMU.1499	NC_004350.2:1421898-1425536	protein_coding	-0.649354363	4.97602E-09
SMU.1500	NC_004350.2:1425526-1428768	protein_coding	-0.24753896	0.037993225
SMU.1502c	NC_004350.2:1429640-1429885	protein_coding	0.041426006	0.957855277

SMU.1504c	NC_004350.2:1430060-1430386	protein_coding	0.803115799	0.150897824
SMU.1505c	NC_004350.2:1430750-1430920	pseudogene	-0.077560396	0.921628598
SMU.1506c	NC_004350.2:1431235-1432515	protein_coding	-0.562775091	0.076013393
SMU.1507c	NC_004350.2:1432512-1432835	protein_coding	-0.853466888	0.054932145
SMU.1508c	NC_004350.2:1432832-1433947	protein_coding	-0.97773328	0.000334449
SMU.1509	NC_004350.2:1434153-1435010	protein_coding	-2.063694655	3.97765E-36
SMU.1510	NC_004350.2:1435221-1437626	protein_coding	-0.097551115	0.511622112
SMU.1511c	NC_004350.2:1437640-1438161	protein_coding	0.099215706	0.683959756
SMU.1512	NC_004350.2:1438234-1439277	protein_coding	0.104843417	0.670028918
SMU.1513	NC_004350.2:1439718-1443254	protein_coding	-0.481231231	2.26037E-06
SMU.1514	NC_004350.2:1443254-1443949	protein_coding	0.166412604	0.420753817
SMU.1515	NC_004350.2:1444056-1444859	protein_coding	2.218813982	4.14787E-84
SMU.1516	NC_004350.2:1444856-1446208	protein_coding	2.007134685	1.62228E-69
SMU.1517	NC_004350.2:1446201-1446908	protein_coding	2.129174712	1.4149E-37
SMU.1519	NC_004350.2:1447261-1448043	protein_coding	2.252693619	1.39198E-76
SMU.1520	NC_004350.2:1448040-1448852	protein_coding	1.777182795	9.24041E-37
SMU.1521	NC_004350.2:1448870-1449574	protein_coding	1.470471373	6.36816E-18
SMU.1522	NC_004350.2:1449589-1450239	protein_coding	0.676524472	8.58015E-06
SMU.1523	NC_004350.2:1450307-1451188	protein_coding	-0.033946389	0.843439241
SMU.1524c	NC_004350.2:1451230-1451418	protein_coding	-0.029663663	0.957855277
SMU.1525	NC_004350.2:1451421-1452692	protein_coding	0.226333699	0.11072408
SMU.1526c	NC_004350.2:1452758-1452967	protein_coding	-1.176116988	0.004049089
SMU.1527	NC_004350.2:1453264-1453680	protein_coding	-2.005808351	3.67223E-98
SMU.1528	NC_004350.2:1453694-1455100	protein_coding	-2.55512402	2.2637E-161
SMU.1529	NC_004350.2:1455126-1456004	protein_coding	-2.764578608	1.8826E-161
SMU.1530	NC_004350.2:1456023-1457528	protein_coding	-2.4079909	1.9558E-183
SMU.1531	NC_004350.2:1457544-1458080	protein_coding	-2.544036708	5.6495E-81
SMU.1532	NC_004350.2:1458080-1458577	protein_coding	-2.311766322	7.51151E-70
SMU.1533	NC_004350.2:1458595-1459314	protein_coding	-2.43494966	1.4398E-99
SMU.1534	NC_004350.2:1459344-1459547	protein_coding	-2.625890758	2.35244E-57
SMU.1535	NC_004350.2:1459786-1462182	protein_coding	-1.579719616	1.54786E-59
SMU.1536	NC_004350.2:1462213-1463643	protein_coding	-1.821742559	1.33847E-76
SMU.1537	NC_004350.2:1463640-1464773	protein_coding	-1.846065558	4.48145E-76
SMU.1538	NC_004350.2:1464763-1465902	protein_coding	-2.024646168	2.9944E-103
SMU.1539	NC_004350.2:1465928-1467814	protein_coding	-1.927240468	3.73711E-69
SMU.1541	NC_004350.2:1468031-1470346	protein_coding	-0.791156769	1.41871E-17
SMU.1542c	NC_004350.2:1470361-1471404	protein_coding	-0.485533999	1.23423E-05
SMU.1543	NC_004350.2:1471417-1473375	protein_coding	-0.236708434	0.013710493
SMU.1545c	NC_004350.2:1473585-1474097	protein_coding	1.507934514	1.15954E-08
SMU.1546	NC_004350.2:1474220-1474678	protein_coding	-0.661520508	0.031754055
SMU.1547c	NC_004350.2:1474761-1475357	protein_coding	-0.092337658	0.817431717
SMU.1548c	NC_004350.2:1475354-1476460	protein_coding	0.268238839	0.171695835
SMU.1550c	NC_004350.2:1476453-1477190	protein_coding	-0.002976671	0.996820397
SMU.1551c	NC_004350.2:1477187-1478068	protein_coding	0.634271965	0.077380724
SMU.1552c	NC_004350.2:1478105-1478470	protein_coding	1.298311387	0.075657258
SMU.1553c	NC_004350.2:1478460-1478654	protein_coding	2.65385569	0.018870296
SMU.1554c	NC_004350.2:1478663-1478860	protein_coding	2.316994793	0.002250737
SMU.1555c	NC_004350.2:1479016-1479948	protein_coding	-0.086897876	0.492239438
SMU.1556	NC_004350.2:1479960-1480820	protein_coding	0.018299317	0.884161823
SMU.1557c	NC_004350.2:1480838-1482121	protein_coding	0.115021022	0.320200076
SMU.1558c	NC_004350.2:1482114-1482668	protein_coding	0.589304352	0.001445068
SMU.1560	NC_004350.2:1483157-1483570	protein_coding	0.665786155	0.139377008
SMU.1561	NC_004350.2:1483658-1484317	protein_coding	-1.991887645	2.99884E-48
SMU.1562	NC_004350.2:1484331-1484984	protein_coding	-2.080417429	4.9752E-59
SMU.1563	NC_004350.2:1485021-1487813	protein_coding	-1.877347386	1.1804E-102
SMU.1564	NC_004350.2:1488085-1490361	protein_coding	-2.212468918	8.6835E-133
SMU.1565	NC_004350.2:1490333-1491862	protein_coding	-1.728685947	2.53492E-54
SMU.1566	NC_004350.2:1491977-1492996	protein_coding	0.6372789	0.000706484
SMU.1568	NC_004350.2:1493243-1494490	protein_coding	-3.53901569	1.1083E-277

SMU.1569	NC_004350.2:1494567-1495928	protein_coding	-2.253901396	2.69167E-74
SMU.1570	NC_004350.2:1495928-1496764	protein_coding	-2.214707053	5.25045E-84
SMU.1571	NC_004350.2:1496789-1497922	protein_coding	-1.888529149	4.4473E-73
SMU.1572	NC_004350.2:1498053-1499312	protein_coding	1.627128661	3.52354E-37
SMU.1573	NC_004350.2:1499456-1500649	protein_coding	0.848731144	9.07399E-07
SMU.1574c	NC_004350.2:1501433-1502632	protein_coding	-1.514619259	1.72167E-29
SMU.1575c	NC_004350.2:1502821-1503105	protein_coding	-2.350439718	8.22535E-19
SMU.1576c	NC_004350.2:1503086-1505134	protein_coding	-1.640091124	5.66433E-20
SMU.1577c	NC_004350.2:1505191-1508940	protein_coding	-1.204791788	4.09364E-18
SMU.1578	NC_004350.2:1509217-1510152	protein_coding	-1.007636452	1.25969E-11
NA	NC_004350.2:1510133-1510327	protein_coding	0.259739427	0.255503586
SMU.1581	NC_004350.2:1510427-1512103	protein_coding	-0.447971004	2.78154E-06
SMU.1582c	NC_004350.2:1512103-1512600	protein_coding	-0.474919829	0.075408456
SMU.1584c	NC_004350.2:1512917-1514686	protein_coding	-0.779628012	0.003415549
SMU.1585c	NC_004350.2:1514805-1515329	protein_coding	-0.293200795	0.411054504
SMU.1586	NC_004350.2:1515633-1517582	protein_coding	-0.896035584	8.21484E-19
SMU.1587c	NC_004350.2:1517579-1518217	protein_coding	-0.068859637	0.798325419
SMU.1588c	NC_004350.2:1518482-1519816	protein_coding	0.319599365	0.010137077
SMU.1589c	NC_004350.2:1519818-1520816	protein_coding	0.236907791	0.037999991
SMU.1590	NC_004350.2:1520936-1522396	protein_coding	0.674062298	4.74183E-09
SMU.1591	NC_004350.2:1522544-1523545	protein_coding	-1.240348964	4.84877E-47
SMU.1592	NC_004350.2:1523708-1524787	protein_coding	-0.851505318	9.32684E-08
SMU.1593c	NC_004350.2:1524878-1525459	protein_coding	-0.250411428	0.157239989
SMU.1595	NC_004350.2:1525586-1526356	protein_coding	-0.019301375	0.944233317
SMU.1596	NC_004350.2:1526622-1527980	protein_coding	-1.384544404	8.47621E-25
SMU.1597c	NC_004350.2:1527996-1528514	protein_coding	-1.566169694	2.8265E-08
SMU.1598	NC_004350.2:1528534-1528845	protein_coding	-1.045413367	0.019816448
SMU.1599	NC_004350.2:1528845-1530836	protein_coding	-1.923185794	1.59285E-28
SMU.1600	NC_004350.2:1530881-1531198	protein_coding	-1.202457966	0.004435235
SMU.1601	NC_004350.2:1531430-1532863	protein_coding	-1.405551128	1.86915E-18
NA	NC_004350.2:1532868-1533101	pseudogene	0.628171687	0.819089501
SMU.1602	NC_004350.2:1533196-1533861	protein_coding	-1.255340204	1.79689E-23
SMU.1603	NC_004350.2:1533897-1534289	protein_coding	-1.351171802	7.81301E-31
SMU.1604c	NC_004350.2:1534623-1535135	protein_coding	0.648768388	0.228626306
SMU.1605	NC_004350.2:1535259-1536944	protein_coding	0.719884619	0.018047066
SMU.1606	NC_004350.2:1536975-1537442	protein_coding	0.60197974	0.003520331
SMU.1607	NC_004350.2:1537405-1539741	protein_coding	0.775230058	1.84789E-08
SMU.1609c	NC_004350.2:1540088-1540324	protein_coding	-0.648718214	0.003326005
SMU.1610	NC_004350.2:1540373-1540519	protein_coding	-0.666275595	0.210190873
SMU.1611c	NC_004350.2:1540519-1541688	protein_coding	-0.705033299	2.81574E-08
SMU.1612c	NC_004350.2:1541810-1542646	protein_coding	-0.887418884	9.07659E-18
SMU.1613c	NC_004350.2:1542639-1543235	protein_coding	-0.926629524	9.22288E-11
SMU.1614	NC_004350.2:1543232-1544053	protein_coding	-0.985988928	1.23267E-16
SMU.1615c	NC_004350.2:1544104-1544628	protein_coding	-0.345025382	0.027086136
SMU.1616c	NC_004350.2:1544728-1545198	protein_coding	-0.359121711	0.076251941
SMU.1617	NC_004350.2:1545243-1546142	protein_coding	-0.350488686	0.033893626
SMU.1618	NC_004350.2:1546163-1546570	protein_coding	-0.175191988	0.606586834
SMU.1619c	NC_004350.2:1546554-1547048	protein_coding	-0.495882454	0.013798624
SMU.1620	NC_004350.2:1547452-1548417	protein_coding	0.476981444	0.001783563
SMU.1621c	NC_004350.2:1548484-1548699	protein_coding	2.245064959	0.006897248
SMU.1622	NC_004350.2:1548696-1549205	protein_coding	1.116707262	4.5564E-06
SMU.1623c	NC_004350.2:1549274-1550134	protein_coding	1.05605447	3.87608E-20
SMU.1624	NC_004350.2:1550379-1550936	protein_coding	0.66767476	5.652E-07
SMU.1625	NC_004350.2:1551287-1552024	protein_coding	0.506366488	6.0631E-05
SMU.1626	NC_004350.2:1552198-1552887	protein_coding	0.549747266	1.71487E-07
SMU.1627	NC_004350.2:1552985-1553410	protein_coding	0.74232479	6.11911E-11
SMU.1628	NC_004350.2:1553598-1553951	protein_coding	-1.313560329	0.00504607
SMU.1629c	NC_004350.2:1554073-1556436	protein_coding	1.008173488	1.76691E-19
SMU.1631	NC_004350.2:1556533-1557309	protein_coding	-0.3652838	0.179378817

SMU.1632	NC_004350.2:1557370-1558065	protein_coding	0.859903662	7.29115E-11
SMU.1633c	NC_004350.2:1558076-1558399	protein_coding	1.390258494	4.5014E-12
SMU.1634c	NC_004350.2:1558411-1558962	protein_coding	1.27646294	7.65072E-22
SMU.1635	NC_004350.2:1558972-1560351	protein_coding	0.811987672	2.38296E-12
SMU.1636c	NC_004350.2:1560390-1560863	protein_coding	0.837066348	2.63747E-06
SMU.1637c	NC_004350.2:1561076-1561282	protein_coding	0.562794987	0.000210052
SMU.1639	NC_004350.2:1561622-1563631	protein_coding	1.144784689	2.82788E-26
SMU.1641c	NC_004350.2:1564209-1564409	protein_coding	0.910846959	9.68084E-05
SMU.1642c	NC_004350.2:1564482-1565114	protein_coding	0.50965433	0.015218281
SMU.1643c	NC_004350.2:1565111-1565564	pseudogene	1.165383847	0.00124816
SMU.1644c	NC_004350.2:1565939-1566742	protein_coding	-0.348690676	0.017851789
SMU.1645	NC_004350.2:1566884-1567765	protein_coding	1.498358366	1.07674E-25
SMU.1646c	NC_004350.2:1567891-1568529	protein_coding	-0.659802824	0.001748579
SMU.1647c	NC_004350.2:1568519-1568797	protein_coding	-1.287286162	4.61298E-10
SMU.1648c	NC_004350.2:1568916-1569161	protein_coding	0.294886083	0.738822022
SMU.1649	NC_004350.2:1569394-1570221	protein_coding	2.702465842	2.6309E-111
SMU.1650	NC_004350.2:1570286-1570909	protein_coding	-0.101891639	0.813683272
SMU.1651	NC_004350.2:1570974-1571327	protein_coding	-1.775518477	3.22973E-26
SMU.1652	NC_004350.2:1571364-1571858	protein_coding	-2.023154207	1.11539E-29
SMU.1653	NC_004350.2:1571947-1573128	protein_coding	-1.615305496	1.99361E-57
SMU.1654c	NC_004350.2:1573214-1573768	protein_coding	-1.320628733	1.70886E-23
SMU.1656	NC_004350.2:1574177-1575268	protein_coding	-1.532628632	5.01751E-34
SMU.1657c	NC_004350.2:1575488-1575829	protein_coding	2.03196616	9.71918E-07
SMU.1658	NC_004350.2:1575845-1577080	protein_coding	1.9604964	3.36149E-10
SMU.1659c	NC_004350.2:1577385-1578251	protein_coding	-0.697516302	4.35096E-05
SMU.1660c	NC_004350.2:1578257-1578589	protein_coding	-0.753213387	0.002935991
SMU.1661c	NC_004350.2:1578582-1579388	protein_coding	-0.639320064	0.000389394
SMU.1662	NC_004350.2:1579385-1580260	protein_coding	-0.696788664	0.000117699
SMU.1663	NC_004350.2:1580273-1580911	protein_coding	-0.95383788	8.64861E-06
SMU.1664c	NC_004350.2:1580988-1581647	protein_coding	0.340458911	0.046679721
SMU.1665	NC_004350.2:1581763-1582473	protein_coding	0.165080892	0.196036492
SMU.1666	NC_004350.2:1582473-1583237	protein_coding	0.123510041	0.290589137
SMU.1667	NC_004350.2:1583237-1584193	protein_coding	0.560194472	1.14071E-05
SMU.1668	NC_004350.2:1584197-1585066	protein_coding	0.520332467	0.000423712
SMU.1669	NC_004350.2:1585150-1586322	protein_coding	0.417443303	1.25249E-05
SMU.1670c	NC_004350.2:1586425-1586673	protein_coding	1.260744574	6.42842E-09
SMU.1671c	NC_004350.2:1586680-1587120	protein_coding	1.213508115	3.5718E-09
SMU.1672	NC_004350.2:1587236-1587826	protein_coding	0.24364779	0.033949038
SMU.1673	NC_004350.2:1588358-1588987	protein_coding	-1.351024381	2.71125E-21
SMU.1674	NC_004350.2:1589300-1590463	protein_coding	-0.304010956	0.144102914
SMU.1675	NC_004350.2:1590478-1591572	protein_coding	0.173039853	0.421236928
SMU.1676c	NC_004350.2:1591799-1593433	protein_coding	-0.646465512	9.08716E-08
SMU.1677	NC_004350.2:1593522-1594973	protein_coding	-1.782622109	1.159E-27
SMU.1678	NC_004350.2:1595207-1596226	protein_coding	-0.752495002	3.21372E-07
SMU.1679c	NC_004350.2:1596404-1596913	protein_coding	-1.251253847	1.02809E-26
SMU.1680c	NC_004350.2:1596939-1597556	protein_coding	-1.512093142	4.13435E-43
SMU.1681c	NC_004350.2:1597560-1597934	protein_coding	-1.561034628	2.87638E-23
SMU.1682c	NC_004350.2:1597995-1598570	protein_coding	0.83910828	0.162907165
SMU.1683c	NC_004350.2:1598634-1599557	protein_coding	-0.616351223	0.003130463
SMU.1685c	NC_004350.2:1599615-1600265	protein_coding	-0.929663657	3.3415E-06
SMU.1687	NC_004350.2:1600472-1601404	protein_coding	-0.726670258	2.3593E-11
SMU.1688	NC_004350.2:1601466-1602731	protein_coding	-0.322517642	0.00411588
SMU.1689	NC_004350.2:1602724-1602963	protein_coding	-0.100499633	0.586720464
SMU.1690	NC_004350.2:1602978-1604240	protein_coding	-0.334545639	0.013766242
SMU.1691	NC_004350.2:1604237-1605787	protein_coding	0.324417281	0.007489737
NA	NC_004350.2:1605797-1605928	protein_coding	0.016552979	0.960795008
SMU.1692	NC_004350.2:1606605-1607396	protein_coding	-0.881639992	4.25319E-10
SMU.1693	NC_004350.2:1607471-1608808	protein_coding	1.452610862	3.43668E-26
SMU.1694c	NC_004350.2:1608844-1609938	protein_coding	-0.158215418	0.449016401

SMU.1695	NC_004350.2:1610015-1610797	protein_coding	-0.089530069	0.582770925
SMU.1697c	NC_004350.2:1610931-1611476	protein_coding	0.068410536	0.786093103
SMU.1699c	NC_004350.2:1611473-1612405	protein_coding	0.54513124	0.001912726
SMU.1700c	NC_004350.2:1612510-1613205	protein_coding	0.545475964	0.014044961
SMU.1701c	NC_004350.2:1613202-1613564	protein_coding	0.603028401	0.144102914
SMU.1702c	NC_004350.2:1613730-1614380	protein_coding	1.386205401	1.56818E-14
SMU.1703c	NC_004350.2:1614370-1614942	protein_coding	2.005299929	2.46545E-22
SMU.1704	NC_004350.2:1615455-1615781	protein_coding	1.274703656	3.02286E-05
SMU.1705	NC_004350.2:1615768-1616364	protein_coding	1.334752055	4.8756E-09
SMU.1706	NC_004350.2:1616361-1617188	protein_coding	0.92372913	3.1352E-08
SMU.1707c	NC_004350.2:1617367-1617915	protein_coding	1.226681708	0.000654957
SMU.1708	NC_004350.2:1618381-1619736	protein_coding	-1.011831679	6.75012E-23
SMU.1709	NC_004350.2:1619743-1621182	protein_coding	-0.526630873	1.99465E-07
SMU.1710c	NC_004350.2:1621249-1621503	protein_coding	0.360028058	0.186597733
SMU.1711	NC_004350.2:1621503-1622225	protein_coding	0.338874077	0.00514905
SMU.1712c	NC_004350.2:1622215-1622811	protein_coding	0.164358428	0.183199888
SMU.1713c	NC_004350.2:1622808-1623515	protein_coding	0.197353186	0.105714708
SMU.1714c	NC_004350.2:1623515-1624252	protein_coding	0.008504003	0.957945292
SMU.1715c	NC_004350.2:1624249-1624710	protein_coding	0.285551195	0.105491777
SMU.1716c	NC_004350.2:1624707-1625225	protein_coding	0.386490536	0.005003693
SMU.1717c	NC_004350.2:1625207-1626184	protein_coding	0.448408947	0.00043999
SMU.1718	NC_004350.2:1626181-1626975	protein_coding	0.032000472	0.808615087
SMU.1719c	NC_004350.2:1627028-1627276	protein_coding	-0.17327281	0.323368127
SMU.1721c	NC_004350.2:1627649-1628899	protein_coding	0.24526079	0.07638417
SMU.1722c	NC_004350.2:1629088-1629777	protein_coding	-0.618988628	1.32471E-06
SMU.1723c	NC_004350.2:1629797-1630300	protein_coding	-0.327550326	0.005538687
SMU.1724c	NC_004350.2:1630335-1631075	protein_coding	-0.497956834	0.04188378
SMU.1725	NC_004350.2:1631108-1631386	protein_coding	-2.066854014	4.33252E-13
SMU.1727	NC_004350.2:1631478-1632410	protein_coding	-0.2595757	0.029547502
SMU.1728	NC_004350.2:1632825-1633307	protein_coding	0.091160928	0.507290632
SMU.1729c	NC_004350.2:1633443-1635365	protein_coding	-0.673039694	2.39135E-09
SMU.1730c	NC_004350.2:1635461-1635952	protein_coding	0.079002838	0.766145256
SMU.1731	NC_004350.2:1636005-1637336	protein_coding	-1.536695721	7.84164E-37
SMU.1732c	NC_004350.2:1637353-1637943	protein_coding	-1.658840056	7.0056E-47
SMU.1733c	NC_004350.2:1638005-1641097	protein_coding	-0.387452385	0.000385076
SMU.1734	NC_004350.2:1641213-1641983	protein_coding	-1.301700321	1.10098E-16
SMU.1735	NC_004350.2:1641980-1642846	protein_coding	-1.448533063	1.47413E-47
SMU.1736	NC_004350.2:1642855-1644225	protein_coding	-1.459383751	3.33827E-56
SMU.1737	NC_004350.2:1644392-1644814	protein_coding	-1.466380375	2.2308E-36
SMU.1738	NC_004350.2:1644811-1645299	protein_coding	-1.389746244	3.55024E-24
SMU.1739	NC_004350.2:1645302-1646534	protein_coding	-1.430566875	1.42665E-49
SMU.1740	NC_004350.2:1646583-1647317	protein_coding	-1.357146839	5.94315E-32
SMU.1741	NC_004350.2:1647332-1648252	protein_coding	-1.244227736	1.14239E-32
SMU.1742c	NC_004350.2:1648255-1649220	protein_coding	-0.724952604	1.20195E-10
SMU.1743	NC_004350.2:1649323-1649547	protein_coding	-1.026884411	0.017177439
SMU.1744	NC_004350.2:1649607-1650584	protein_coding	-0.500577265	0.00032048
SMU.1745c	NC_004350.2:1650584-1651018	protein_coding	-0.691289925	0.00013888
SMU.1746c	NC_004350.2:1651347-1652138	protein_coding	-0.199309658	0.102424619
SMU.1747c	NC_004350.2:1652300-1652944	protein_coding	1.269200724	2.96336E-14
SMU.1748	NC_004350.2:1653043-1654401	protein_coding	0.059614836	0.723762092
SMU_t41	NC_004350.2:1654785-1654858	tRNA	2.499350894	0.128253796
SMU_t42	NC_004350.2:1654911-1654984	tRNA	1.993954725	0.469502634
SMU_t43	NC_004350.2:1654998-1655083	tRNA	-2.437395417	0.001192603
SMU_t44	NC_004350.2:1655091-1655162	tRNA	NA	NA
SMU_t45	NC_004350.2:1655165-1655237	tRNA	0.495202664	0.922599346
SMU_t46	NC_004350.2:1655252-1655333	tRNA	NA	NA
SMU_t47	NC_004350.2:1655343-1655415	tRNA	NA	NA
SMU_t48	NC_004350.2:1655443-1655515	tRNA	NA	NA
SMU_t49	NC_004350.2:1655545-1655617	tRNA	NA	NA

SMU_r07	NC_004350.2:1655622-1655737	rRNA	NA	NA
SMU_r08	NC_004350.2:1655920-1658820	rRNA	NA	NA
SMU_t50	NC_004350.2:1659077-1659149	tRNA	NA	NA
SMU_r09	NC_004350.2:1659209-1660765	rRNA	-1.428379326	0.6965518
SMU.1753c	NC_004350.2:1662432-1662725	protein_coding	2.077163644	9.03226E-40
NA	NC_004350.2:1662736-1663759	pseudogene	2.09293047	5.09566E-79
SMU.1758c	NC_004350.2:1663756-1664427	protein_coding	1.598263843	5.13508E-45
SMU.1760c	NC_004350.2:1664429-1665304	protein_coding	1.474362238	5.1122E-45
SMU.1761c	NC_004350.2:1665308-1666795	protein_coding	2.117056055	5.28077E-46
SMU.1763c	NC_004350.2:1667240-1667983	protein_coding	1.772039172	4.0208E-38
SMU.1764c	NC_004350.2:1668084-1670492	protein_coding	1.71933178	1.36265E-68
SMU.1765c	NC_004350.2:1670737-1671711	protein_coding	0.239270363	0.169623871
NA	NC_004350.2:1672007-1672252	protein_coding	-0.379502881	0.005279126
SMU.1768c	NC_004350.2:1672391-1672696	protein_coding	-0.456286759	0.000101908
SMU.1770	NC_004350.2:1673267-1675918	protein_coding	-0.46323891	5.51061E-08
SMU.1771c	NC_004350.2:1675930-1676238	protein_coding	-0.459663014	0.617208004
SMU.1772c	NC_004350.2:1676210-1676620	protein_coding	-0.784447325	1.83987E-06
SMU.1773c	NC_004350.2:1676613-1677548	protein_coding	-0.55469211	0.003986826
SMU.1774c	NC_004350.2:1677838-1678092	protein_coding	-0.392712449	0.142390595
SMU_t51	NC_004350.2:1678247-1678317	tRNA	4.19775913	3.98453E-06
SMU.1775c	NC_004350.2:1678588-1678767	protein_coding	0.015219952	0.951979836
SMU.1776c	NC_004350.2:1678998-1679243	protein_coding	-0.707450082	0.000202466
SMU.1777	NC_004350.2:1679497-1679958	protein_coding	0.107882754	0.646909268
SMU.1779c	NC_004350.2:1679968-1681347	protein_coding	0.191224486	0.186040485
SMU.1780	NC_004350.2:1681383-1682159	protein_coding	-2.965820911	6.17283E-54
SMU.1781	NC_004350.2:1682245-1682778	protein_coding	-0.66901881	1.20083E-05
SMU.1782	NC_004350.2:1682838-1683158	protein_coding	1.468871627	1.33247E-05
SMU_t52	NC_004350.2:1683397-1683460	tRNA	1.1300617	0.438234701
SMU_t53	NC_004350.2:1683467-1683539	tRNA	0.495205187	0.871609965
SMU_t54	NC_004350.2:1683554-1683635	tRNA	NA	NA
SMU_t55	NC_004350.2:1683645-1683717	tRNA	0	1
SMU_t56	NC_004350.2:1683745-1683817	tRNA	NA	NA
SMU_t57	NC_004350.2:1683847-1683919	tRNA	NA	NA
SMU_r10	NC_004350.2:1683924-1684039	rRNA	-0.466589037	0.838490514
SMU_r11	NC_004350.2:1684222-1687122	rRNA	2.288634806	0.355372755
SMU_t58	NC_004350.2:1687379-1687451	tRNA	NA	NA
SMU_r12	NC_004350.2:1687511-1689067	rRNA	0.429757466	0.843509373
SMU.1783	NC_004350.2:1689405-1691255	protein_coding	0.477557531	3.96485E-05
SMU.1784c	NC_004350.2:1691282-1692541	protein_coding	0.799503299	1.0216E-11
SMU.1785	NC_004350.2:1692557-1693351	protein_coding	1.010331574	6.45935E-12
SMU.1786	NC_004350.2:1693366-1694115	protein_coding	1.361324994	9.11486E-15
SMU.1787c	NC_004350.2:1694227-1694610	protein_coding	0.475361549	0.042823784
SMU.1788c	NC_004350.2:1694785-1695132	protein_coding	1.04724684	6.1444E-10
SMU.1789c	NC_004350.2:1695325-1696041	protein_coding	0.998801651	2.53473E-14
SMU.1790c	NC_004350.2:1696187-1696903	protein_coding	2.20012372	1.85092E-51
SMU.1791c	NC_004350.2:1697007-1698107	protein_coding	2.181119949	1.02455E-41
SMU.1794c	NC_004350.2:1698501-1698731	protein_coding	2.093584327	1.97151E-15
SMU.1795c	NC_004350.2:1698792-1699535	protein_coding	2.334988441	1.6761E-54
SMU.1797c	NC_004350.2:1699809-1700162	protein_coding	1.882150097	2.34763E-13
SMU.1798c	NC_004350.2:1700163-1700756	protein_coding	1.15168176	1.72925E-21
SMU.1799	NC_004350.2:1700753-1701385	protein_coding	1.283465811	3.62492E-28
SMU.1800c	NC_004350.2:1701412-1701720	protein_coding	1.66064225	5.24842E-17
SMU.1801c	NC_004350.2:1701777-1702883	protein_coding	1.390640902	2.54708E-38
SMU.1802c	NC_004350.2:1702884-1703411	protein_coding	1.448560748	1.26094E-19
SMU.1803c	NC_004350.2:1703516-1704133	protein_coding	-0.663940224	1.15447E-07
SMU.1805	NC_004350.2:1704488-1705138	protein_coding	-0.329037927	0.418782429
SMU.1806	NC_004350.2:1705116-1706285	protein_coding	-0.126309265	0.598115271
SMU.1807c	NC_004350.2:1706316-1707224	protein_coding	0.531733668	0.005204216
SMU.1808c	NC_004350.2:1707319-1707414	protein_coding	0.402726973	0.452148789

SMU.1809	NC_004350.2:1707788-1708519	protein_coding	0.341310538	0.115718245
SMU.1810	NC_004350.2:1708519-1709271	protein_coding	0.249658823	0.456574583
SMU.1811	NC_004350.2:1709273-1710181	protein_coding	0.967168451	0.000338653
SMU.1812	NC_004350.2:1710468-1711718	protein_coding	1.671161065	0.014952338
SMU.1814	NC_004350.2:1712304-1713683	protein_coding	0.975662996	4.23765E-16
SMU.1815	NC_004350.2:1713701-1714399	protein_coding	0.793055014	2.46395E-09
NA	NC_004350.2:1714504-1715457	pseudogene	1.262096574	3.98453E-05
SMU.1818c	NC_004350.2:1715576-1715749	protein_coding	-0.681756915	0.096181149
SMU.1819	NC_004350.2:1716002-1717441	protein_coding	-0.781052882	1.2488E-14
SMU.1820c	NC_004350.2:1717441-1718907	protein_coding	-0.881972723	6.31501E-18
SMU.1821c	NC_004350.2:1718907-1719209	protein_coding	-0.547218844	0.008576714
SMU.1822	NC_004350.2:1719336-1721087	protein_coding	-0.761458974	1.58858E-09
SMU.1823	NC_004350.2:1721345-1721896	protein_coding	0.072718825	0.660666533
SMU.1824c	NC_004350.2:1721898-1722683	protein_coding	-0.117961145	0.383799136
SMU.1826	NC_004350.2:1722792-1724006	protein_coding	-1.04634568	1.4068E-21
SMU.1827	NC_004350.2:1724183-1724719	protein_coding	5.207475857	1.48107E-24
SMU.1828	NC_004350.2:1724817-1725266	protein_coding	-0.761239959	2.34603E-09
SMU.1830c	NC_004350.2:1725544-1726944	protein_coding	0.641514071	1.52426E-08
SMU.1831	NC_004350.2:1727002-1727961	protein_coding	-0.025216901	0.894757061
SMU.1833	NC_004350.2:1728295-1730310	protein_coding	0.724340743	4.61594E-09
SMU.1834	NC_004350.2:1730483-1731598	protein_coding	0.006431353	0.960795008
SMU.1835	NC_004350.2:1731595-1731954	protein_coding	-0.082416255	0.535973865
SMU.1836	NC_004350.2:1732224-1733258	protein_coding	-0.251939706	0.009933808
SMU.1837	NC_004350.2:1733260-1734291	protein_coding	0.042706093	0.842601979
SMU.1838	NC_004350.2:1734311-1736830	protein_coding	0.344599859	3.27976E-05
SMU.1839	NC_004350.2:1737008-1737958	protein_coding	-0.009043576	0.931014955
SMU.1840	NC_004350.2:1738127-1739008	protein_coding	0.281134365	0.004987047
SMU.1841	NC_004350.2:1739208-1741202	protein_coding	-1.334982419	1.96873E-37
SMU.1843	NC_004350.2:1741398-1742837	protein_coding	-2.109913036	2.70536E-59
SMU.1844	NC_004350.2:1742840-1743802	protein_coding	-1.748071936	9.72102E-46
SMU.1845	NC_004350.2:1743996-1744424	protein_coding	0.671255034	0.000307208
SMU.1846c	NC_004350.2:1744417-1744806	protein_coding	0.548083757	5.07549E-05
SMU.1847	NC_004350.2:1744849-1745409	protein_coding	0.690409109	1.07482E-08
SMU.1848	NC_004350.2:1745549-1746253	protein_coding	-0.289321846	0.158209347
SMU.1849	NC_004350.2:1746301-1746753	protein_coding	1.205138292	1.14253E-17
SMU.1850	NC_004350.2:1746923-1747987	protein_coding	0.42363803	0.000698729
SMU.1851	NC_004350.2:1747977-1750808	protein_coding	1.090902877	1.7897E-25
NA	NC_004350.2:1750858-1751079	protein_coding	-0.027562993	0.963547068
SMU.1852	NC_004350.2:1751479-1752423	protein_coding	0.201627223	0.158272886
SMU.1853	NC_004350.2:1752705-1753367	protein_coding	0.015204943	0.923450668
SMU.1854	NC_004350.2:1753506-1753862	protein_coding	-1.501261088	1.67751E-12
SMU.1855	NC_004350.2:1753859-1754545	protein_coding	-1.598755342	5.2011E-26
SMU.1856c	NC_004350.2:1754553-1755272	protein_coding	1.733397358	5.39716E-19
SMU.1858	NC_004350.2:1755591-1755830	protein_coding	0.836541374	5.08339E-08
SMU.1859	NC_004350.2:1755859-1756353	protein_coding	0.657475995	6.2539E-10
SMU.1860	NC_004350.2:1756371-1756661	protein_coding	0.410796524	0.005196579
SMU.1861c	NC_004350.2:1756817-1757062	protein_coding	-0.25449434	0.097796952
SMU.1862	NC_004350.2:1757367-1757570	protein_coding	-1.775676418	8.02102E-06
SMU.1865	NC_004350.2:1758607-1759752	protein_coding	2.002527763	3.51516E-37
SMU.1867c	NC_004350.2:1759940-1760989	protein_coding	1.429883381	5.36509E-28
SMU.1869	NC_004350.2:1761087-1761401	protein_coding	0.91102306	2.01836E-07
SMU.1870	NC_004350.2:1761476-1763806	protein_coding	0.899142694	4.24734E-14
SMU.1871c	NC_004350.2:1763875-1764420	protein_coding	0.370473574	0.086677159
SMU.1872c	NC_004350.2:1764417-1764722	protein_coding	-0.344383218	0.113051439
SMU.1873	NC_004350.2:1764918-1765829	protein_coding	0.861063861	7.01877E-10
SMU.1874	NC_004350.2:1765849-1766436	protein_coding	0.736039321	7.30711E-06
SMU.1875	NC_004350.2:1766522-1768885	protein_coding	0.779380973	1.02922E-08
SMU.1876	NC_004350.2:1768984-1769454	protein_coding	0.419411676	0.049775388
SMU.1877	NC_004350.2:1770331-1771323	protein_coding	-1.128081006	1.25662E-23

SMU.1878	NC_004350.2:1771358-1772176	protein_coding	-0.044242862	0.760379724
SMU.1879	NC_004350.2:1772191-1773117	protein_coding	-1.243619663	6.64354E-39
SMU.1881c	NC_004350.2:1773449-1775740	protein_coding	1.952121012	1.4068E-21
SMU.1882c	NC_004350.2:1776290-1776643	protein_coding	1.53745643	5.30414E-06
SMU.1883	NC_004350.2:1776962-1777330	protein_coding	0.470953736	0.008041648
SMU.1884c	NC_004350.2:1777567-1778640	protein_coding	4.084619348	6.80372E-05
SMU.1886	NC_004350.2:1779164-1780444	protein_coding	1.617829948	3.95576E-45
SMU.1889c	NC_004350.2:1781127-1781390	protein_coding	-0.39092124	0.390247549
SMU.1892c	NC_004350.2:1781694-1781879	protein_coding	0.119300217	0.893910586
NA	NC_004350.2:1781999-1783347	protein_coding	1.428683101	0.521307877
SMU.1895c	NC_004350.2:1783456-1783617	protein_coding	1.059078137	7.92091E-09
SMU.1896c	NC_004350.2:1783662-1783916	protein_coding	0.330992692	0.349080913
SMU.1898	NC_004350.2:1784304-1786489	pseudogene	-1.132448091	2.96548E-08
SMU.1900	NC_004350.2:1786502-1787515	protein_coding	-2.527116431	8.8067E-06
SMU.1904c	NC_004350.2:1788212-1789234	protein_coding	-4.286674085	0
SMU.1905c	NC_004350.2:1789720-1789908	protein_coding	-4.633404921	0
SMU.1906c	NC_004350.2:1790072-1790284	protein_coding	-4.645164448	1.3712E-285
SMU.1909c	NC_004350.2:1791628-1792032	protein_coding	-4.57258922	0
SMU.1910c	NC_004350.2:1792179-1792598	protein_coding	-4.289814593	1.1975E-257
SMU.1913c	NC_004350.2:1793979-1794380	protein_coding	-4.049477657	8.5156E-227
SMU.1914c	NC_004350.2:1794511-1794741	protein_coding	-2.628147221	3.26295E-81
SMU.1915	NC_004350.2:1795008-1795148	protein_coding	2.255006077	0.049328469
SMU.1916	NC_004350.2:1795290-1796615	protein_coding	-1.463178715	2.73973E-29
SMU.1917	NC_004350.2:1796612-1797364	protein_coding	-1.753404069	7.29289E-41
SMU.1918	NC_004350.2:1797836-1798474	protein_coding	0.309049209	0.010945798
SMU.1919	NC_004350.2:1798521-1799147	protein_coding	-0.229956719	0.04044761
SMU.1920	NC_004350.2:1799222-1800532	protein_coding	0.085784144	0.537125962
SMU.1921	NC_004350.2:1800545-1801444	protein_coding	0.217034404	0.099612382
SMU.1922	NC_004350.2:1801445-1802614	protein_coding	-0.047221918	0.683959756
SMU.1923c	NC_004350.2:1802601-1803092	protein_coding	-0.011886582	0.95866659
SMU.1924	NC_004350.2:1803462-1804154	protein_coding	-0.892841609	9.54808E-11
SMU.1925c	NC_004350.2:1804511-1805047	protein_coding	0.636835709	0.013551141
SMU.1926	NC_004350.2:1805040-1805615	protein_coding	0.303378409	0.149820218
SMU.1927	NC_004350.2:1805723-1806430	protein_coding	0.005529727	0.989549132
SMU.1928	NC_004350.2:1806432-1809044	protein_coding	-0.482420252	0.011543766
SMU.1929	NC_004350.2:1809181-1810080	protein_coding	0.470876856	0.000153921
SMU.1930	NC_004350.2:1810090-1810650	protein_coding	0.696092779	9.18565E-05
SMU.1931	NC_004350.2:1810738-1811451	protein_coding	-0.983260157	1.28305E-07
SMU.1933c	NC_004350.2:1811675-1812508	protein_coding	-0.205294976	0.179325288
SMU.1934c	NC_004350.2:1812501-1814177	protein_coding	-0.912534701	9.59844E-15
SMU.1935c	NC_004350.2:1814206-1814751	protein_coding	-0.583223855	0.006760985
SMU.1936c	NC_004350.2:1814761-1815606	protein_coding	-0.838448729	1.89165E-05
SMU.1937	NC_004350.2:1815730-1816506	protein_coding	-1.229868773	1.24973E-12
SMU.1938c	NC_004350.2:1816574-1817263	protein_coding	-0.962265946	1.39807E-05
SMU.1939c	NC_004350.2:1817265-1818329	protein_coding	-0.644781949	1.02507E-05
SMU.1940c	NC_004350.2:1818322-1819695	protein_coding	-0.649122435	0.000124144
SMU.1941	NC_004350.2:1819890-1820732	protein_coding	-0.847826288	1.0631E-10
SMU.1942c	NC_004350.2:1820899-1821702	protein_coding	-0.627967221	4.49841E-06
SMU.1943	NC_004350.2:1822003-1824504	protein_coding	0.187055077	0.076904846
SMU.1945	NC_004350.2:1824905-1825669	protein_coding	0.001326582	0.996017407
SMU.1946	NC_004350.2:1825662-1826351	protein_coding	0.231284512	0.251600387
SMU.1947	NC_004350.2:1826568-1827104	protein_coding	1.002854781	4.13212E-06
SMU.1948	NC_004350.2:1827260-1827436	protein_coding	1.55964986	5.91196E-08
NA	NC_004350.2:1827448-1827600	protein_coding	0.453750835	0.05267418
SMU.1949	NC_004350.2:1827651-1829927	protein_coding	0.334943152	0.00100048
SMU.1950	NC_004350.2:1829971-1830837	protein_coding	0.21889109	0.391137205
SMU.1951c	NC_004350.2:1830857-1832560	protein_coding	0.872015678	1.12771E-12
SMU.1954	NC_004350.2:1833204-1834832	protein_coding	0.004514057	0.960427324
SMU.1955	NC_004350.2:1834944-1835231	protein_coding	0.410993975	0.00066588

SMU.1956c	NC_004350.2:1835353-1835649	protein_coding	-4.991212337	3.4653E-192
SMU.1957	NC_004350.2:1835673-1836509	protein_coding	-4.540281233	5.0733E-245
SMU.1958c	NC_004350.2:1836511-1837362	protein_coding	-4.571908821	0
SMU.1960c	NC_004350.2:1837736-1838230	protein_coding	-4.960594233	7.6209E-305
SMU.1961c	NC_004350.2:1838248-1838679	protein_coding	-4.67946379	1.6312E-155
NA	NC_004350.2:1838712-1838915	protein_coding	-0.98784427	0.246976081
SMU.1963c	NC_004350.2:1838982-1840289	protein_coding	-1.117196024	1.17427E-19
SMU.1964c	NC_004350.2:1840282-1840962	protein_coding	-0.967307991	1.19445E-06
SMU.1965c	NC_004350.2:1840962-1842284	protein_coding	-1.144600398	2.47E-16
SMU.1966c	NC_004350.2:1842284-1843246	protein_coding	-1.385741589	6.028E-14
SMU.1967	NC_004350.2:1843662-1844057	protein_coding	-4.741974791	1.8826E-161
SMU.1968c	NC_004350.2:1844144-1844500	protein_coding	1.303058617	0.00429236
SMU.1969c	NC_004350.2:1844500-1844922	protein_coding	2.37713513	2.85206E-06
SMU.1970c	NC_004350.2:1845020-1845646	protein_coding	0.828512853	0.001133185
SMU.1971c	NC_004350.2:1845643-1845972	protein_coding	0.961491909	0.00711556
SMU.1972c	NC_004350.2:1845950-1846258	protein_coding	0.919469669	0.020723888
SMU.1973	NC_004350.2:1846318-1847385	protein_coding	-0.03364644	0.84476877
SMU.1974	NC_004350.2:1847441-1848211	protein_coding	-0.31175445	0.050303026
SMU.1975c	NC_004350.2:1848296-1848958	protein_coding	-1.425716669	1.69024E-30
SMU.1976c	NC_004350.2:1849011-1849451	protein_coding	-0.738567986	8.47879E-09
SMU.1977c	NC_004350.2:1849420-1849665	protein_coding	-0.820001453	5.07549E-05
SMU.1978	NC_004350.2:1850234-1851433	protein_coding	-3.443524143	7.3482E-133
SMU.1979c	NC_004350.2:1851492-1852445	protein_coding	-4.335908474	1.5685E-165
SMU.1980c	NC_004350.2:1852500-1852889	protein_coding	-4.424863131	3.8588E-128
SMU.1981c	NC_004350.2:1852867-1853301	protein_coding	-4.526001305	4.9731E-136
SMU.1982c	NC_004350.2:1853288-1853581	protein_coding	-4.378330992	3.83874E-55
SMU.1983	NC_004350.2:1853553-1853984	protein_coding	-4.792254079	6.7715E-186
SMU.1984	NC_004350.2:1853944-1854258	protein_coding	-4.641097861	2.4393E-177
SMU.1985	NC_004350.2:1854258-1855235	protein_coding	-4.321559231	3.6171E-140
SMU.1987	NC_004350.2:1855225-1856166	protein_coding	-3.935991004	7.2736E-120
SMU.1988c	NC_004350.2:1856296-1856679	protein_coding	1.343331229	3.20033E-07
SMU.1989	NC_004350.2:1856807-1860463	protein_coding	-0.004616063	0.969429677
SMU.1990	NC_004350.2:1860672-1864235	protein_coding	0.808179109	9.74168E-13
SMU.1991	NC_004350.2:1864496-1866850	protein_coding	0.479199176	7.53471E-07
SMU.1992	NC_004350.2:1867018-1868274	protein_coding	0.76145005	2.69444E-07
SMU.1993	NC_004350.2:1868430-1869242	protein_coding	1.123351624	2.65813E-06
SMU.1994	NC_004350.2:1869232-1869942	protein_coding	1.660629194	3.14336E-12
SMU.1995c	NC_004350.2:1869945-1870391	protein_coding	1.73485081	1.34286E-06
SMU.1996	NC_004350.2:1871026-1871874	protein_coding	0.345767976	0.06676442
SMU.1997	NC_004350.2:1872021-1872503	protein_coding	1.509869766	4.11191E-11
SMU_t59	NC_004350.2:1873122-1873195	tRNA	5.736593671	4.9366E-05
SMU_r13	NC_004350.2:1873201-1873316	rRNA	0.940873296	0.000702914
SMU_r14	NC_004350.2:1873499-1876399	rRNA	-1.42837471	0.762676718
SMU_t60	NC_004350.2:1876656-1876728	tRNA	NA	NA
SMU_r15	NC_004350.2:1876788-1878344	rRNA	NA	NA
SMU.1999c	NC_004350.2:1878521-1879807	protein_coding	1.352449478	3.76051E-15
SMU.2000	NC_004350.2:1880033-1880419	protein_coding	0.670816753	4.86794E-07
SMU.2001	NC_004350.2:1880438-1881376	protein_coding	0.503033638	6.16682E-07
SMU.2002	NC_004350.2:1881422-1881805	protein_coding	0.466930874	0.000589129
SMU.2003	NC_004350.2:1881823-1882188	protein_coding	0.419958433	0.002221357
SMU.2003a	NC_004350.2:1882208-1882324	protein_coding	0.874695523	6.36439E-08
SMU.2004	NC_004350.2:1882350-1882568	protein_coding	0.308052311	0.041120773
SMU.2005	NC_004350.2:1882687-1883325	protein_coding	-1.079828913	1.91737E-14
SMU.2006	NC_004350.2:1883555-1884859	protein_coding	-0.489451559	4.26378E-06
SMU.2007	NC_004350.2:1884859-1885299	protein_coding	-1.085533965	1.2227E-36
SMU.2008	NC_004350.2:1885592-1885774	protein_coding	-0.732601042	7.33393E-10
SMU.2009	NC_004350.2:1885788-1886282	protein_coding	-0.906342165	4.74888E-21
SMU.2010	NC_004350.2:1886301-1886657	protein_coding	-1.064090602	4.49473E-26
SMU.2011	NC_004350.2:1886747-1887283	protein_coding	-0.909450552	5.87287E-27

SMU.2012	NC_004350.2:1887696-1888094	protein_coding	-1.190037069	2.15476E-36
SMU.2014	NC_004350.2:1888351-1888536	protein_coding	-0.974062993	1.24879E-25
SMU.2015	NC_004350.2:1888551-1889093	protein_coding	-1.099561487	3.67697E-32
SMU.2016	NC_004350.2:1889117-1889422	protein_coding	-1.02740646	8.30462E-17
SMU.2017	NC_004350.2:1889588-1889956	protein_coding	-1.164319735	4.69154E-27
SMU.2018	NC_004350.2:1889983-1890243	protein_coding	-0.835148614	9.30266E-15
SMU.2019	NC_004350.2:1890264-1890473	protein_coding	-0.94288239	1.32152E-11
SMU.2020	NC_004350.2:1890483-1890896	protein_coding	-0.829973083	1.27224E-15
SMU.2021	NC_004350.2:1890900-1891553	protein_coding	-0.581275782	1.01832E-09
SMU.2022	NC_004350.2:1891566-1891910	protein_coding	-0.361968363	0.000585607
SMU.2023c	NC_004350.2:1891927-1892205	protein_coding	-0.186354009	0.115718245
SMU_2167	NC_004350.2:1892307-1893146	protein_coding	-0.29384541	0.001851397
SMU_2166	NC_004350.2:1893164-1893463	protein_coding	-0.042953937	0.744768352
SMU.2024c	NC_004350.2:1893515-1894138	protein_coding	0.268108187	0.023500114
SMU.2025	NC_004350.2:1894163-1894789	protein_coding	0.71202253	3.38227E-16
NA	NC_004350.2:1895024-1895332	protein_coding	1.097897513	3.51469E-19
SMU.2027	NC_004350.2:1895652-1896338	protein_coding	-1.142295397	2.6038E-27
SMU.2028	NC_004350.2:1896529-1898916	protein_coding	1.316472702	2.55832E-29
SMU.2029	NC_004350.2:1899203-1901644	protein_coding	0.219544112	0.033712411
SMU.2030	NC_004350.2:1901641-1902105	protein_coding	0.522696824	0.000527277
SMU.2031	NC_004350.2:1902386-1903432	protein_coding	-0.061285904	0.619391209
SMU.2032	NC_004350.2:1903499-1904284	protein_coding	0.312469048	0.000987535
SMU.2033c	NC_004350.2:1904475-1906328	protein_coding	0.758403565	3.53792E-08
SMU_t61	NC_004350.2:1906607-1906677	tRNA	4.398964755	0.001181945
SMU.2035	NC_004350.2:1907122-1908078	protein_coding	-0.932375683	7.16147E-19
SMU.2036	NC_004350.2:1908223-1910118	protein_coding	0.678697862	9.87487E-12
SMU.2037	NC_004350.2:1910627-1912255	protein_coding	-0.135641898	0.374221726
SMU.2038	NC_004350.2:1912281-1914248	protein_coding	0.251534379	0.080875894
SMU.2040	NC_004350.2:1914422-1915135	protein_coding	1.061483702	4.8745E-07
SMU.2042	NC_004350.2:1915481-1918033	protein_coding	0.861178029	9.19593E-21
SMU.2043c	NC_004350.2:1918518-1918964	protein_coding	0.638494765	0.00056851
SMU.2044	NC_004350.2:1918971-1921193	protein_coding	1.400946887	1.65326E-32
SMU.2046c	NC_004350.2:1921341-1922162	protein_coding	-0.724318308	2.30311E-06
SMU.2047	NC_004350.2:1922253-1924442	protein_coding	-1.632569285	1.29441E-78
NA	NC_004350.2:1924701-1924895	pseudogene	2.23270658	0.022738603
SMU.2049c	NC_004350.2:1925281-1926024	protein_coding	-0.670499984	6.20557E-07
SMU.2050c	NC_004350.2:1926025-1926978	protein_coding	-0.694039184	3.47928E-10
SMU.2052c	NC_004350.2:1927206-1927503	pseudogene	-1.066708342	4.68045E-07
SMU.2054c	NC_004350.2:1927516-1927986	protein_coding	-0.667534158	0.001851397
SMU.2055	NC_004350.2:1928134-1928625	protein_coding	0.134124818	0.521307877
SMU.2056	NC_004350.2:1928615-1929880	protein_coding	0.165250894	0.193461545
NA	NC_004350.2:1929950-1930143	ncRNA	-2.885781679	1.91595E-69
SMU_t62	NC_004350.2:1930143-1930215	tRNA	2.702711589	0.090115768
SMU.2057c	NC_004350.2:1930287-1932202	pseudogene	0.166452986	0.123792056
SMU.2058	NC_004350.2:1932301-1933095	protein_coding	1.116165491	2.30488E-08
SMU.2059c	NC_004350.2:1933280-1934305	protein_coding	0.563276655	0.059687431
SMU.2060	NC_004350.2:1934410-1935285	protein_coding	0.246587761	0.044167369
SMU.2061	NC_004350.2:1935354-1936007	protein_coding	-0.427756211	0.00100048
SMU.2063	NC_004350.2:1936213-1937172	protein_coding	-0.230951114	0.069773602
SMU.2064c	NC_004350.2:1937248-1940046	protein_coding	-0.051888678	0.712459436
SMU.2065	NC_004350.2:1940179-1941195	protein_coding	-0.004368459	0.976076093
SMU.2066c	NC_004350.2:1941230-1944154	protein_coding	0.140969083	0.191297444
SMU.2067	NC_004350.2:1944151-1945110	protein_coding	0.245041955	0.104170072
SMU.2069	NC_004350.2:1945717-1946511	protein_coding	0.672747044	0.010728473
SMU.2070	NC_004350.2:1946538-1947266	protein_coding	0.071053227	0.831524114
SMU.2071	NC_004350.2:1947352-1947957	protein_coding	1.499843595	1.7387E-12
SMU.2072c	NC_004350.2:1947917-1948474	protein_coding	2.247605983	5.91067E-51
SMU.2074	NC_004350.2:1948736-1950940	protein_coding	0.890872301	1.23003E-18
SMU.2075c	NC_004350.2:1951039-1952610	protein_coding	0.486305347	2.97022E-05

SMU.2077c	NC_004350.2:1952844-1953143	protein_coding	1.270465943	8.30498E-13
SMU.2078c	NC_004350.2:1953224-1953643	protein_coding	1.123099409	3.16056E-21
SMU.2079c	NC_004350.2:1953640-1953909	protein_coding	0.730967045	1.74688E-05
SMU.2080	NC_004350.2:1954077-1954511	protein_coding	1.097501845	0.062670564
SMU.2081	NC_004350.2:1954524-1954970	protein_coding	1.354115781	0.004841364
NA	NC_004350.2:1954961-1955173	protein_coding	0.083033877	0.51990689
SMU.2083c	NC_004350.2:1955300-1955725	protein_coding	0.663640305	3.44714E-07
SMU.2084c	NC_004350.2:1955976-1956374	protein_coding	-0.702667751	3.71357E-09
SMU.2085	NC_004350.2:1956459-1957610	protein_coding	-1.2017561	1.89273E-30
SMU.2086	NC_004350.2:1957650-1958906	protein_coding	-2.111202011	9.54004E-64
SMU.2087	NC_004350.2:1959055-1959615	protein_coding	0.45149728	0.011699147
SMU.2088	NC_004350.2:1959697-1960290	protein_coding	0.620156282	6.57704E-05
SMU.2089	NC_004350.2:1960473-1962428	protein_coding	0.924429531	1.36042E-10
NA	NC_004350.2:1962653-1962931	pseudogene	3.738679921	7.86958E-08
SMU.2091c	NC_004350.2:1962926-1965475	protein_coding	0.421148744	0.000253085
SMU.2092c	NC_004350.2:1965468-1965815	protein_coding	1.151281766	1.66549E-08
SMU.2093	NC_004350.2:1965812-1966249	protein_coding	1.313057421	6.35402E-09
SMU.2094c	NC_004350.2:1966672-1967169	protein_coding	0.570374368	0.041520978
NA	NC_004350.2:1968106-1968546	pseudogene	0.078615122	0.594918872
SMU.2098	NC_004350.2:1968647-1970338	protein_coding	0.251126591	0.027293362
SMU.2099c	NC_004350.2:1970386-1971264	protein_coding	-0.742225202	2.26075E-08
SMU.2100c	NC_004350.2:1971295-1972233	protein_coding	-1.095804768	2.90322E-20
SMU.2101	NC_004350.2:1972220-1973989	protein_coding	-0.883799552	6.88437E-17
SMU.2102	NC_004350.2:1974220-1975509	protein_coding	0.564425855	9.20905E-07
SMU.2104	NC_004350.2:1975802-1977646	protein_coding	-0.030695293	0.840266892
SMU.2104a	NC_004350.2:1977775-1977957	protein_coding	-0.327534429	0.055022912
NA	NC_004350.2:1977973-1978122	protein_coding	-0.369934594	0.007920112
SMU.2106c	NC_004350.2:1978397-1978654	protein_coding	-0.585592405	0.207222662
SMU.2107c	NC_004350.2:1978676-1978945	protein_coding	0.537945379	0.294069659
SMU.2108c	NC_004350.2:1978971-1980068	protein_coding	0.501399132	0.034447724
SMU.2109	NC_004350.2:1980163-1981524	protein_coding	2.463405917	2.77501E-31
SMU.2111c	NC_004350.2:1981648-1982040	protein_coding	-0.419475995	0.249907393
SMU.2112	NC_004350.2:1982239-1983933	protein_coding	-0.719069055	6.65788E-13
SMU.2113c	NC_004350.2:1984090-1984635	protein_coding	0.409288038	0.158272886
SMU.2114c	NC_004350.2:1984701-1985075	protein_coding	0.091727436	0.798325419
SMU.2115	NC_004350.2:1985154-1985909	protein_coding	0.937249171	0.034848361
SMU.2116	NC_004350.2:1986361-1987512	protein_coding	3.311404205	5.9246E-115
SMU.2117	NC_004350.2:1987509-1988141	protein_coding	2.905105646	1.44656E-62
SMU.2118	NC_004350.2:1988143-1989078	protein_coding	2.164220271	1.05567E-59
SMU.2119	NC_004350.2:1989078-1989743	protein_coding	1.320452229	7.99433E-14
SMU.2120c	NC_004350.2:1989826-1990404	protein_coding	-0.390270934	0.037916837
SMU.2121c	NC_004350.2:1990430-1990717	protein_coding	0.363045071	0.429823666
SMU.2123	NC_004350.2:1990921-1991544	protein_coding	0.452713575	0.114101564
SMU.2125	NC_004350.2:1991626-1991910	protein_coding	-0.282867662	0.368366834
SMU.2126c	NC_004350.2:1991940-1992701	protein_coding	0.496992206	0.076902564
SMU.2127	NC_004350.2:1992887-1994263	protein_coding	-2.328342649	2.71977E-92
SMU.2128	NC_004350.2:1994475-1996190	protein_coding	-1.314082759	1.15046E-34
SMU.2129c	NC_004350.2:1996343-1996684	protein_coding	1.639107164	0.000350268
SMU.2130	NC_004350.2:1996873-1997817	protein_coding	-0.171374863	0.481394745
SMU.2133c	NC_004350.2:1998008-2000512	protein_coding	3.147400857	3.01742E-75
SMU.2134	NC_004350.2:2000644-2001198	protein_coding	-0.527001728	0.072463917
SMU.2135c	NC_004350.2:2001240-2001851	protein_coding	0.442529693	0.002612335
SMU.2137c	NC_004350.2:2002151-2002423	protein_coding	0.197125552	0.355372755
SMU.2138	NC_004350.2:2002437-2003801	protein_coding	0.65289467	2.24731E-08
SMU.2139c	NC_004350.2:2003822-2004274	protein_coding	0.408457146	0.017851789
SMU.2140c	NC_004350.2:2004276-2006249	protein_coding	1.254557045	2.02921E-25
SMU.2141	NC_004350.2:2006329-2008224	protein_coding	1.544725151	1.17783E-29
SMU.2142	NC_004350.2:2008351-2008983	protein_coding	0.433420047	0.007378497
SMU.2143c	NC_004350.2:2009045-2010166	protein_coding	0.860096614	7.0756E-14

SMU.2146c	NC_004350.2:2010535-2011140	protein_coding	2.919037967	5.43218E-48
SMU.2147c	NC_004350.2:2011302-2012168	protein_coding	2.6825392	6.9999E-102
SMU.2148c	NC_004350.2:2012357-2013151	protein_coding	0.21573229	0.247293538
SMU.2149c	NC_004350.2:2013144-2013986	protein_coding	0.298925394	0.094673128
SMU.2150c	NC_004350.2:2013962-2014804	protein_coding	-0.27483102	0.025212223
SMU.2151	NC_004350.2:2014808-2015350	protein_coding	-0.548254584	0.004116895
SMU.2152c	NC_004350.2:2015362-2016339	protein_coding	-0.22749376	0.094673128
SMU.2153c	NC_004350.2:2016412-2017704	protein_coding	-0.172672805	0.397032086
SMU.2154c	NC_004350.2:2017706-2018953	protein_coding	0.15422267	0.404110456
SMU.2155	NC_004350.2:2019032-2019397	protein_coding	-1.582218304	2.67445E-10
SMU.2156	NC_004350.2:2019400-2020491	protein_coding	-1.16549695	4.75849E-20
SMU.2157	NC_004350.2:2020654-2022135	protein_coding	1.140172897	8.88891E-25
SMU.2158c	NC_004350.2:2022292-2023314	protein_coding	1.122183462	9.90839E-11
SMU.2159	NC_004350.2:2023747-2025366	protein_coding	0.098394331	0.394638309
SMU.2160	NC_004350.2:2025406-2027979	protein_coding	1.155134711	8.53812E-11
SMU_t63	NC_004350.2:2028186-2028259	tRNA	0.495202664	0.922599346
SMU_t64	NC_004350.2:2028265-2028336	tRNA	-0.488984952	0.7132393
SMU_t65	NC_004350.2:2028677-2028750	tRNA	4.211717692	4.71684E-06
SMU.2161c	NC_004350.2:2028771-2029433	protein_coding	0.018654207	0.949495232
SMU.2162c	NC_004350.2:2029776-2030255	protein_coding	-0.250968736	0.38279334
SMU.2164	NC_004350.2:2030461-2031669	protein_coding	0.697294359	1.62075E-10
SMU.2165	NC_004350.2:2032144-2032917	protein_coding	1.106991136	2.34245E-08
NA	NC_004350.2:99732-99869	protein_coding	1.107561061	0.048348468
SMU.379	NC_004350.2:359137-359274	protein_coding	2.735883931	0.178394789
SMU.594	NC_004350.2:552595-552750	protein_coding	0.783805505	0.351087736
SMU.948	NC_004350.2:897864-898034	protein_coding	1.158936505	2.71367E-05
SMU.1256c	NC_004350.2:1189999-1190163	protein_coding	-0.299413828	0.178394789
SMU.1435c	NC_004350.2:1367788-1367949	protein_coding	2.043372496	0.003567483
SMU.1459c	NC_004350.2:1388991-1389164	protein_coding	-0.342263164	0.05181304
SMU.1655c	NC_004350.2:1573919-1574059	protein_coding	-1.307890149	3.69104E-12
NA	NC_004350.2:1661309-1662115	pseudogene	3.135671092	2.8617E-138
SMU.1832	NC_004350.2:1727988-1728152	protein_coding	0.143095396	0.667690501
SMU.1902c	NC_004350.2:1787777-1787920	protein_coding	-4.421343901	1.4543E-220
SMU.1908c	NC_004350.2:1790689-1790853	protein_coding	-4.610315151	5.2011E-301
SMU.2136c	NC_004350.2:2001955-2002131	protein_coding	0.539669166	0.022007151
NA	NC_004350.2:1518211-1518403	pseudogene	-0.291931718	0.365376357
NA	NC_004350.2:1563924-1564108	pseudogene	2.22990247	0.040859871
NA	NC_004350.2:1924923-1925219	protein_coding	-0.697693504	2.03273E-05
NA	NC_004350.2:95724-96461	pseudogene	1.09232404	0.113903172
SMU.108	NC_004350.2:109280-109417	protein_coding	-2.810136337	7.28139E-17
NA	NC_004350.2:213755-214100	pseudogene	0.256089873	0.164720052
NA	NC_004350.2:214140-214355	pseudogene	0.123127475	0.577896779
NA	NC_004350.2:293306-293469	pseudogene	1.037674105	0.006148156
NA	NC_004350.2:596329-596652	protein_coding	0.176245071	0.700509033
SMU.687c	NC_004350.2:652698-652832	protein_coding	0.059813676	0.963547068
NA	NC_004350.2:1192125-1192241	pseudogene	1.964419299	0.099181565
NA	NC_004350.2:1301468-1302033	pseudogene	1.825247907	0.000412943
NA	NC_004350.2:1303605-1304160	pseudogene	1.104420326	0.0029106
NA	NC_004350.2:1304343-1304747	pseudogene	-0.19894998	0.692686296
NA	NC_004350.2:1323964-1324194	protein_coding	5.358136071	3.24517E-49
NA	NC_004350.2:1395758-1396185	pseudogene	0.090572083	0.830946301
SMU.1762c	NC_004350.2:1666677-1667240	protein_coding	1.643860553	3.9613E-16
NA	NC_004350.2:1791293-1791505	pseudogene	1.149821298	0.26701726
NA	NC_004350.2:1910203-1910541	pseudogene	-0.02663584	0.922599346
